# Effectiveness of Iodophor vs Chlorhexidine Solutions for Surgical Site Infections and Unplanned Reoperations for Patients Who Underwent Fracture Repair: The PREP-IT Master Protocol

**DOI:** 10.1001/jamanetworkopen.2020.2215

**Published:** 2020-04-07

**Authors:** The Program of Randomized Trials to Evaluate Pre-operative Antiseptic Skin Solutions in Orthopaedic Trauma (PREP-IT) Investigators, Gerard P. Slobogean, Sheila Sprague, Jeffrey Wells, Mohit Bhandari, Alejandra Rojas, Alisha Garibaldi, Amber Wood, Andrea Howe, Anthony D. Harris, Bradley A. Petrisor, Daniel C. Mullins, David Pogorzelski, Debra Marvel, Diane Heels-Ansdell, Franca Mossuto, Frances Grissom, Gina Del Fabbro, Gordon H. Guyatt, Gregory J. Della Rocca, Haley K. Demyanovich, I. Leah Gitajn, Jana Palmer, Jean-Claude D’Alleyrand, Jeff Friedrich, Jessica Rivera, Joan Hebden, Joshua Rudnicki, Justin Fowler, Kyle J. Jeray, Lehana Thabane, Lucas Marchand, Lyndsay M. O’Hara, Manjari G. Joshi, Max Talbot, Megan Camara, Olivia Paige Szasz, Nathan N. O’Hara, Paula McKay, P. J. Devereaux, Robert V. O’Toole, Robert Zura, Saam Morshed, Shannon Dodds, Silvia Li, Stephanie L. Tanner, Taryn Scott, Uyen Nguyen

**Affiliations:** 1 Department of Orthopaedics, University of Maryland School of Medicine, Baltimore; 2 Division of Orthopaedic Surgery, Department of Surgery, McMaster University, Hamilton, Ontario, Canada; 3 Trauma Survivors Network, Falls Church, Virginia; 4 Association of periOperative Registered Nurses, Denver, Colorado; 5 Department of Epidemiology and Public Health, University of Maryland School of Medicine, Baltimore; 6 Department of Pharmaceutical Health Services Research, University of Maryland School of Pharmacy, Baltimore; 7 Patient Advisor, Baltimore, Maryland; 8 Department of Health Research Methods, Evidence, and Impact, McMaster University, Hamilton, Ontario, Canada; 9 Hamilton Health Science, Hamilton, Ontario, Canada; 10 Trauma Survivor Network, Baltimore, Maryland; 11 Department of Orthopaedic Surgery, University of Missouri, Columbia; 12 Department of Orthopaedics, Dartmouth University, Hanover, New Hampshire; 13 Department of Orthopaedic Surgery, Walter Reed National Military Medical Center, Bethesda, Maryland; 14 Washington, DC; 15 Department of Orthopaedic Surgery, San Antonio Military Medical Center, San Antonio, Texas; 16 Department of Orthopaedic Surgery, Greenville Health System, Greenville, South Carolina; 17 Department of Orthopaedic Surgery, University of Utah, Salt Lake City; 18 Department of Medicine, University of Maryland School of Medicine, Baltimore; 19 Canadian Armed Forces, Montreal, Qubec, Canada; 20 Department of Medicine, McMaster University, Hamilton, Ontario, Canada; 21 Department of Orthopaedics, Louisiana State University Health, New Orleans; 22 Department of Orthopaedic Surgery, University of California, San Francisco

## Abstract

**Importance:**

The risk of developing a surgical site infection after extremity fracture repair is nearly 5 times greater than in most elective orthopedic surgical procedures. For all surgical procedures, it is standard practice to prepare the operative site with an antiseptic solution; however, there is limited evidence to guide the choice of solution used for orthopedic fracture repair.

**Objective:**

To compare the effectiveness of iodophor vs chlorhexidine solutions to reduce surgical site infections and unplanned fracture-related reoperations for patients who underwent fracture repair.

**Design, Setting, and Participants:**

The PREP-IT (Program of Randomized Trials to Evaluate Pre-operative Antiseptic Skin Solutions in Orthopaedic Trauma) master protocol will be followed to conduct 2 multicenter pragmatic cluster randomized crossover trials, Aqueous-PREP (Pragmatic Randomized Trial Evaluating Pre-Operative Aqueous Antiseptic Skin Solution in Open Fractures) and PREPARE (Pragmatic Randomized Trial Evaluating Pre-Operative Alcohol Skin Solutions in Fractured Extremities). The Aqueous-PREP trial will compare 4% aqueous chlorhexidine vs 10% povidone-iodine for patients with open extremity fractures. The PREPARE trial will compare 2% chlorhexidine in 70% isopropyl alcohol vs 0.7% iodine povacrylex in 74% isopropyl alcohol for patients with open extremity fractures and patients with closed lower extremity or pelvic fractures. Both trials will share key aspects of study design and trial infrastructure. The studies will follow a pragmatic cluster randomized crossover design with alternating treatment periods of approximately 2 months. The primary outcome will be surgical site infection and the secondary outcome will be unplanned fracture-related reoperations within 12 months. The Aqueous-PREP trial will enroll a minimum of 1540 patients with open extremity fractures from at least 12 hospitals; PREPARE will enroll a minimum of 1540 patients with open extremity fractures and 6280 patients with closed lower extremity and pelvic fractures from at least 18 hospitals. The primary analyses will adhere to the intention-to-treat principle and account for potential between-cluster and between-period variability. The patient-centered design, implementation, and dissemination of results are guided by a multidisciplinary team that includes 3 patients and other relevant stakeholders.

**Discussion:**

The PREP-IT master protocol increases efficiency through shared trial infrastructure and study design components. Because prophylactic skin antisepsis is used prior to all surgical procedures and the application, cost, and availability of all study solutions are similar, the results of the PREP-IT trials are poised to inform clinical guidelines and bring about an immediate change in clinical practice.

**Trial Registration:**

ClinicalTrials.gov Identifiers: NCT03385304 and NCT03523962

## Key Points

**Question:**

What is the effectiveness of surgical skin preparation with iodophor solutions vs chlorhexidine solutions at reducing 90-day surgical site infections and unplanned fracture-related reoperations within 1 year of injury?

**Findings:**

This trial master protocol describes 2 multicenter pragmatic cluster randomized crossover trials, Aqueous-PREP (Pragmatic Randomized Trial Evaluating Pre-Operative Aqueous and Antiseptic Skin Solution in Open Fractures) and PREPARE (Pragmatic Randomized Trial Evaluating Pre-Operative Alcohol Skin Solutions in Fractured Extremities), which seek to compare the effectiveness of iodophor and chlorhexidine surgical skin preparation solutions at reducing surgical site infections and unplanned fracture-related reoperations.

**Meaning:**

Because prophylactic skin antisepsis is used prior to all surgical procedures and the application, cost, and availability of all study solutions are similar, the results are poised to inform clinical guidelines and bring about change in clinical practice.

## Teaser

This trial protocol describes 2 multicenter pragmatic cluster randomized crossover trials comparing the effectiveness of iodophor and chlorhexidine surgical skin preparation solutions at reducing surgical site infections (SSIs) and unplanned fracture-related reoperations.

## Introduction

More than 1 million US individuals sustain an extremity fracture (broken bone in the arm, leg, or pelvis) that requires surgery each year.^1,2^ Approximately 5% of patients with surgical extremity fractures (approximately 50 000 patients) develop a surgical site infection (SSI),^3,4^ which is twice the rate among most surgical patients and nearly 5 times the rate among patients undergoing elective orthopedic surgery (eg, joint replacement).^4^ The prevention of SSIs is an important goal of perioperative care of patients with extremity fractures. Standard operating room practice in the management of extremity fractures includes prophylactic antibiotics, sterile technique, and skin preparation with an antiseptic solution. The available solutions kill bacteria and decrease the quantity of native skin flora, thereby decreasing SSIs.^5,6,7,8^ The most common skin preparation solutions include either an iodophor-based or chlorhexidine-based active ingredient and are delivered in an alcohol-based or aqueous-based solution.

The evidence guiding choices for surgical site skin antisepsis prior to fracture surgery is largely extrapolated from other disciplines. There are at least 2 large randomized clinical trials comparing iodophor and chlorhexidine solutions in other surgical populations^5,6^; however, heterogeneity in treatment comparisons, populations restricted to abdominal and genitourinary surgery, and the use of 30-day SSI outcomes suggest that extrapolation of the existing evidence from other surgical disciplines to fracture surgery could be problematic. Although the superiority of chlorhexidine or iodophors for orthopedic surgery remains unclear, the implications of a fracture’s associated soft-tissue trauma, local vascular disruption, and the routine implantation of metal fixation suggest that the differential effectiveness between patients with fractures and the previous study populations is plausible. Moreover, previous microbiological analyses suggest that bacterial biofilms (often present with implant-associated infections) may be associated with reduced chlorhexidine susceptibility, and iodophors may have a wider spectrum of antimicrobial activity. Although chlorhexidine resistance has not emerged as a major clinical problem, these previous findings suggest a potential mechanism for improved outcomes with iodophor antiseptic solutions for orthopedic fracture repair.

Currently, to our knowledge, the only data available on the effectiveness of surgical skin preparations for extremity fracture repair come from secondary analysis of the Fluid Lavage in Open Wounds (FLOW) trial.^3^ Specifically, multivariable analyses of 2447 patients with open fractures found that, compared with chlorhexidine solutions, iodophor-based skin antiseptic preparation solutions may be protective against complications (adjusted hazard ratio, 0.88; 95% CI, 0.69-1.12). However, the wide 95% CI suggests that iodophor solutions may reduce the odds of infection by as much as 31% or increase it by as much as 12%, leaving its superiority to prevent SSIs unresolved.

Furthermore, while the primary rationale for using antiseptic skin preparation solutions is to reduce the risk of SSIs, many complications of fracture healing are associated with indolent infections. These low-grade infections typically do not exhibit clinical signs consistent with SSIs; instead, they present several months after fracture fixation and are only detected from deep-tissue samples collected during secondary surgery to treat fractures that fail to heal (nonunion). Previous fracture nonunion studies have identified an infectious cause in 31% to 38% of cases.^9,10^ Therefore, because iodophors may be more effective in preventing SSIs, it is clinically plausible that the use of iodophors may also reduce unplanned fracture-related reoperations. Given the lack of directly applicable evidence and the inadequate duration of follow-up in previous trials, there is a need for large, rigorous clinical trials comparing antiseptic skin solutions used in fracture care surgery.

The PREP-IT trials (Program of Randomized Trials to Evaluate Pre-operative Antiseptic Skin Solutions in Orthopaedic Trauma) represent the use of a master protocol to conduct 2 trials poised to address these gaps in the literature. To our knowledge, a master protocol has not been used to conduct comparative effectiveness fracture care research, and its use will provide many efficiencies in study design, data collection, and trial infrastructure.

## Methods

### Master Protocol Overview

The PREP-IT master protocol includes 2 multicenter pragmatic cluster randomized crossover trials that will study 4 antiseptic solutions in 3 independent populations of patients surgically treated for fracture (Table 1): the Aqueous-PREP trial (A Pragmatic Randomized Trial Evaluating Pre-operative Aqueous Antiseptic Skin Solutions in Open Fractures) (trial protocol in Supplement 1) and the PREPARE trial (A Pragmatic Randomized Trial Evaluating Pre-operative Alcohol Skin Solutions in Fractured Extremities) (trial protocol in Supplement 2).

**Table 1.** Summary of Study Solutions and Fracture Populations

Trial name	Study population	Solution	Comparator	Minimum sample size, No. of patients
Aqueous-PREP	Patients with open appendicular fractures	10% Povidone-iodine in purified water	4% Chlorhexidine in purified water	1540
PREPARE^a^	Patients with open appendicular fractures	Iodine povacrylex 0.7% free iodine in 74% isopropyl alcohol	2% Chlorhexidine in 70% isopropyl alcohol	1540
	Patients with closed lower extremity and pelvic fractures			6280

Abbreviations: Aqueous-PREP, Pragmatic Randomized Trial Evaluating Pre-Operative Aqueous Antiseptic Skin Solution in Open Fractures; PREPARE, Pragmatic Randomized Trial Evaluating Pre-Operative Alcohol Skin Solutions in Fractured Extremities.

^a^ Two separate study populations will be recruited and analyzed independently.

The cluster randomized crossover trial design is a novel research method that is gaining popularity for studying infection control interventions and several other medical treatments. The primary benefit of this study design is that all patients with fractures treated at the recruiting hospitals will receive the predetermined study intervention prior to patient enrollment. This efficiency maximizes recruitment feasibility because study consent does not need to occur prior to the patient’s urgent surgery, and it minimizes selection bias to improve internal and external study validity. Similarly, the crossover method ensures that each participating hospital uses both treatments and acts as an internal control to minimize between-cluster variability. In each trial, the unit of randomization will be the orthopedic practices within the clinical sites (clusters), with individual participants being the unit of analysis.

The PREP-IT trials are registered at ClinicalTrials.gov (Aqueous-PREP, NCT03385304; PREPARE, NCT03523962). Ethics approval has been obtained from the Hamilton Integrated Research Ethics Board for the Methods Center, the Adverra Central Institutional Review Board, and each clinical site’s local institutional review board or research ethics board, if they are not using the central institutional review board. Written informed consent will be obtained for study participation. Because all potential participants will receive the current antiseptic solution being used at the cluster, the consent process focuses on the participant’s willingness to be assessed for the duration of the 1-year study period.

The following details described in this master protocol article are a summary of the study specific protocols: Aqueous-PREP, version 2.1, and PREPARE, version 2.1. The protocols were developed in accordance with recommendations from the Standard Protocol Items: Recommendations for Interventional Trials (SPIRIT) 2013 Statement: Defining Standard Protocol Items for Clinical Trials.^11^

### Overarching Objectives and Hypotheses

The overarching objective of this master protocol is to compare the effectiveness of common iodophor and chlorhexidine antiseptic surgical skin preparation solutions for extremity fracture surgery. We hypothesize that iodophor solutions are a more effective surgical skin preparation solution than chlorhexidine to reduce 90-day SSIs and unplanned fracture-related reoperations within 1 year of injury.

### Study Populations With Open or Closed Fractures 

As described in Table 1, participants with open or closed fractures will be enrolled separately to compare the independent effectiveness of the study solutions in each population. Patients with open fractures and those with closed fractures represent 2 distinct populations within extremity fracture surgery. Open fractures are associated with an SSI incidence that is approximately 4 times greater than that of closed fractures.^3,4^ The increased baseline risk, differing fracture treatment principles, and the difference between having (open) and not having (closed) deep tissue exposed to microorganisms at the time of injury provides a biological rationale for maintaining separate populations with open and closed fractures. This rationale is further strengthened by data collected from our surgeon survey that suggests that many surgeons use different antiseptic skin preparations for open and closed fracture surgical procedures. Therefore, definitively comparing the effectiveness of the study solutions in each fracture population addresses distinctly different treatment decisions for surgeons. Similarly, if a difference in the effectiveness between the 2 study solutions were detected in only 1 of the fracture populations, it would be an independently important clinical finding that would most likely have an immediate effect on clinical practice. Finally, the aqueous solutions will not be compared among patients with closed fractures because they are rarely used in this population.

### Subgroup Objectives and Hypotheses

The PREP-IT trials will also explore the possibility of differential treatment effects of the preoperative antiseptic skin solutions among clinically important subgroups within each independent fracture population. The credibility of all subgroup analyses will be assessed in accordance with criteria outlined by Sun et al.^12^

Our subgroup hypotheses are guided by previous observations that several patient and injury factors are frequently associated with worse patient outcomes after extremity fractures, and a differential treatment effect of the solutions is biologically plausible.^13,14^ These factors include known differences in patients’ skin flora based on anatomical region of injury, and previous trial data identifying high-grade soft-tissue injury (Gustilo-Anderson type III), lower extremity open fractures, and moderate or severe wound contamination are factors associated with SSIs and reoperations among patients with open fractures (I.L. Gitajn, MD, et al, written communication, 2020). Because of its broader spectrum of antimicrobial activity, the increased effectiveness observed by Swenson et al,^7^ the possible benefits observed in the FLOW trial (open fracture populations),^3^ and increased antiseptic longevity of iodine povacrylex (PREPARE populations),^15^ we hypothesize that the iodophor antiseptic skin solutions will be associated with a larger reduction in odds for SSIs and reoperation among patients with more severe soft-tissue injury, increased comorbidities, more severely contaminated wounds, and lower extremity fractures.

The open fracture subgroups will be defined by (1) the severity of open fracture (Gustilo-Anderson type I or II vs type III),^16^ (2) upper extremity vs lower extremity open fractures, and (3) the severity of wound contamination. The PREPARE trial will also include the presence or absence of comorbidities that affect wound healing as a subgroup analysis. The closed fracture subgroups (PREPARE) will be defined by (1) severity of soft-tissue injury (Tscherne grade 3 vs grades 0-2) and (2) presence or absence of comorbidities that affect wound healing.

### Pragmatic-Explanatory Continuum

In accordance with recommended methodological standards, we have used the Pragmatic-Explanatory Continuum Indicator Summary (PRECIS-2)^17^ toolkit to evaluate the design decisions of the PREP-IT trials to determine whether these decisions will lead to a study that answers, “Does this intervention work under usual conditions?” (pragmatic) vs “Can this intervention work under ideal conditions?” (explanatory). Table 2 outlines the investigators’ assessment of the trial design and the rationale for each assessed score.

**Table 2.** PRECIS-2 Score

Domain	Score	Rationale
Eligibility	5	Eligibility criteria are very broad and include all patients with fractures who would be treated in all hospital environments.
Recruitment	5	Recruitment of all consenting patients with fractures treated at each participating hospital will be performed.
Setting	4	Recruitment is occurring at multiple sites across the US and Canada; however, since most of the recruiting hospitals are regional referral centers the setting is mostly pragmatic.
Organization	5	The interventions do not need an increase in clinicians or care delivery compared with the usual antiseptic care provided. For each antiseptic solution, a brief in-service training session will be provided to the clinical sites, as per any new product or procedure that is being introduced into an operating room.
Flexibility (delivery)	5	The interventions will be delivered in the usual care manner with no advice on allowed cointerventions or strict protocols to ensure compliance.
Flexibility (adherence)		This section is left blank according to PRECIS-2 guidance because the intervention is provided prior to patient consent and individual patient adherence is not an issue. If clinician compliance is considered, the study design is rather pragmatic because there will be limited encouragement to follow the manufacturer’s directions for use, other than periodic newsletters, investigator meetings, and possible clinician survey during the recruitment period.
Follow-up	5	All study follow-up is consistent with usual care.
Primary outcome	5	The outcome has been validated by patients as being very relevant to the study participants and it does not require specialized expertise beyond the treating physician for diagnosis.
Primary analysis	5	All available study data will be used for analysis following the intention-to-treat principle.

Abbreviation: PRECIS-2, Pragmatic-Explanatory Continuum Indicator Summary.

### Study Setting, Cluster Eligibility, and Selection of Clusters

This study will be coordinated by the Methods Center at the Centre for Evidence-Based Orthopaedics, McMaster University, Hamilton, Ontario, Canada, and by the Administrative Center within the Department of Orthopaedics at the University of Maryland School of Medicine, R Adams Cowley Shock Trauma Center, Baltimore, Maryland. Patients will be enrolled from approximately 25 clinical sites in North America. Clusters (orthopedic practices at trauma centers) will be carefully screened prior to participation in Aqueous-PREP and PREPARE. Cluster inclusion criteria are as follows: (1) adequate research personnel infrastructure to manage the study, (2) adequate fracture volume to complete enrollment within the study timeline, (3) commitment from all or most orthopedic surgeons to participate in the trial, and (4) ability to use the 2 skin preparation solutions being compared. The exclusion criteria are as follows: (1) lack of interest in the trial; (2) anticipated challenges with complying with the protocol; (3) conflicting studies, in the judgment of the principal investigators, that would inhibit patient participation; and (4) budgeting or contract constraints. Study personnel will carefully assess potential clinical sites and document reasons for clinical site ineligibility. Based on the site eligibility criteria, participating sites will most likely be regional referral centers; however, additional efforts to include sites of varying geographical location and size will be made. On selection, clinical sites will be asked to complete a questionnaire that will detail current surgeon preferences and practices for preoperative surgical preparation techniques and cointerventions known to be associated with the incidence of SSIs.

### Eligibility Criteria

Broad eligibility criteria will be used to increase the generalizability of the trials. The inclusion criteria are as follows:

1. Patients 18 years of age or older
2. Eligible fracture within the Aqueous-PREP or PREPARE populations:
  - Open fracture of the appendicular skeleton (Aqueous-PREP)
  - Open fracture of the appendicular skeleton, excluding hand fractures (PREPARE open fracture cohort)
  - Closed fracture of the lower extremity or pelvis (PREPARE closed fracture cohort)
3. Received or will receive definitive fracture treatment with a surgical implant(s) (eg, internal fixation, external fixation, or joint prosthesis)
4. Open fracture wound management that includes formal surgical debridement within 72 hours of the injury (open fractures) or fracture management that requires a surgical incision (closed fractures)
5. Will have all planned fracture care surgical procedures performed by a participating surgeon or delegate
6. Informed consent obtained
7. Patient enrolled within 3 weeks of the fracture (Aqueous-PREP and PREPARE open fracture cohort) or within 6 weeks of the fracture (PREPARE closed fracture cohort)

The exclusion criteria are as follows:

1. Fracture of the hand (PREPARE open fracture cohort)
2. Patients who did not or will not receive the allocated preoperative surgical preparation solution owing to a medical contraindication
3. Receipt of previous surgical debridement (Aqueous-PREP and PREPARE open fracture cohort) or surgical management of the fracture at a nonparticipating hospital or clinic
4. Fracture managed outside of the participating orthopedic service
5. Chronic or acute infection at or near the fracture site at the time of initial fracture surgery
6. Burns at the fracture site
7. Incarceration
8. Expected injury survival of less than 90 days
9. Terminal illness with expected survival less than 90 days
10. Currently enrolled in a study that does not permit coenrollment
11. Unable to obtain informed consent owing to language barriers
12. Likely problems, in the judgment of study personnel, with maintaining follow-up with the patient
13. Prior or current enrollment in a PREP-IT trial
14. Excluded owing to a sampling strategy

### Patient Screening and Consent

For the Aqueous-PREP trial and the PREPARE open fracture cohort, patients 18 years of age or older who present to a participating clinical site for treatment of an eligible fracture will be screened for participation. For the PREPARE closed fracture cohort, only patients who require surgical treatment will be screened for participation. Study participants must be enrolled within 3 weeks of their fracture(s) for the Aqueous-PREP trial and the PREPARE open fracture cohort and within 6 weeks of their fracture for the PREPARE closed fracture cohort. Potentially eligible patients, or their proxy health care decision maker, will be approached to participate in the trial, even if they did not receive the correct preoperative antiseptic skin solution. This procedure is consistent with the intention-to-treat (ITT) principle and is necessary to maintain the prognostic balance achieved during the cluster randomization.

### Interventions

The interventions for the Aqueous-PREP trial are aqueous preoperative antiseptic skin preparations with 10% povidone-iodine vs 4% chlorhexidine. Products that list other inactive ingredients will be permitted. The manufacturer of each product may vary across the selected clinical sites. Methods Center personnel will review the label of each potential product and confirm that it is acceptable for use for the trial.

For the PREPARE trial, the iodine-based treatment intervention will be an antiseptic solution composed of iodine povacrylex (0.7% free iodine) in 74% isopropyl alcohol. DuraPrep (3M Health Care) will be the commercial product used. The chlorhexidine solution will contain 2% chlorhexidine in 70% isopropyl alcohol as the only active ingredients. ChloraPrep (CareFusion Inc) will be the commercial product used.

Patients will receive the initially allocated treatment solution for all their fracture management surgical procedures, including repeated planned surgical procedures, even if a planned subsequent surgery occurs during a recruitment period using the nonallocated solution. If a fracture requires multiple surgical procedures and the correct solution is not applied at each procedure, the patient will remain in the study and be analyzed in the treatment group in which they were enrolled (ITT principle).

### Blinding

The orthopedic team cannot be blinded to the treatment allocation because the antiseptic solutions are visually distinguishable and these individuals need to lead the implementation of the cluster-crossover protocol at their clinical site. The adjudication committee and data analysts will be blinded to the study treatment. All interpretations of results for each trial will initially be performed in a blinded manner by developing 2 interpretations of the results. Once the data interpretations for each assumption are finalized, the data will be unblinded, and the correct interpretation will be accepted.^18^

### Randomization and Initial Run-in Phase

**Figure 1.**
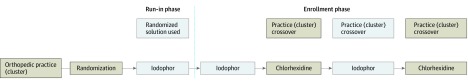
Randomized Treatment Allocation, Cluster Crossover, and Recruitment

Prior to initiating patient recruitment, each clinical site will be randomized to determine which study solution to begin using (Figure 1). A run-in period will be initiated to ensure that acceptable compliance is met before initiating participant enrollment. The run-in phase will occur simultaneously in the open and closed fracture populations for clinical sites participating in PREPARE. Acceptable compliance during the run-in phase will be defined as at least 15 eligible patients with fractures per study population, with more than 90% of eligible patients receiving the allocated antiseptic solution, or a minimum of 1 month.

### Subsequent Intervention Phases

Once the first intervention phase is complete, the cluster will crossover to the opposite study solution. The process of alternating treatment periods (crossovers) will occur approximately every 2 months. The 2-month period was selected to balance seasonal variability in incidence of SSIs and their associated infectious organisms.^19,20^ In addition, for clinical sites enrolling beyond 12 months, the distribution of recruitment periods for each solution may be seasonally matched by reversing the order of the alternating allocation after 12 months of recruitment. The 2-month crossover periods will maintain interest in the trials at the participating clinical sites and allow for the stopping of clinical sites after 4-month intervals if there are issues with performance or overenrollment.

### Application of Preoperative Antiseptic Skin Solutions

Each solution will be applied to the skin by operating room personnel and will be allowed to dry according to the product’s directions for use. Local study personnel will provide in-service training for orthopedic surgeons, operating room technicians, and nurses at each participating clinical site prior to the initial run-in phase. The study protocol mandates the antiseptic skin solution to be used in each intervention phase; however, the protocol will remain pragmatic to variability in the actual application of the solutions and other cointervention steps performed during the entire preoperative skin preparation process performed in the operating room. Based on individual surgeon preference, this variability often includes mechanically removing visible dirt or debris with a scrub brush and/or cleaning the limb with isopropyl alcohol or an antiseptic scrub solution. These additional skin preparation steps will be permitted, provided that (1) the final skin preparation step prior to surgical incision is the application of the allocated antiseptic solution to the skin of the operative extremity and (2) participating surgeons continue to use the same skin preparation cointerventions in both intervention phases. Cointerventions that contain the opposite active ingredient from the current intervention phase should be avoided; however, deviations from this recommendation will be permitted, to maintain pragmatic flexibility of delivery and reflect real-world clinical practice. The details of all operating room antiseptic cointerventions will be documented.

### Perioperative Cointerventions

To optimize the internal validity of the trial findings, key details of cointerventions known to be associated with the incidence of SSIs will be documented. Cointerventions include operating room procedures, such as prescrubbing the limb with an antiseptic brush or placing topical antibiotics into the surgical wound, and non–operating room interventions, such as preoperative chlorhexidine baths. As a result, cointerventions may be implemented by the surgeon or hospital. For example, hospitals typically implement standard procedures to achieve quality process benchmarks designed to minimize SSIs.^1,2,16^ Although these guidelines mandate core benchmark processes to minimize SSIs, it is not practical or generalizable for the trial protocol to standardize the steps taken or cointerventions performed to achieve these core measures because each participating hospital will already have its own implemented procedures. This is the primary rationale for the cluster-crossover design, in which each participating hospital will act as its own control for the effect of cointerventions. Therefore, the following 4 key approaches to account for and limit the potential differential application of cointerventions during the study periods will be performed: (1) study periods for each intervention are kept relatively short to improve the likelihood that newly implemented cointerventions will be equally distributed across both treatment solutions, and (2) participating surgeons will be encouraged to not make changes to their existing infection prevention interventions during the study periods, (3) to document the cointerventions being used in the hospitals throughout the study periods, and (4) to record any changes in cointerventions that do occur if mandated by a participating hospital’s administration. To this end, a monitoring tool containing a list of commonly applied prophylactic cointerventions being used at the participating clinical sites will be completed approximately every 4 months to document any changes to their infection prevention strategies during the study period.

### Primary Outcome

The primary outcome is SSI, guided by the 2017 Centers for Disease Control and Prevention (CDC) National Healthcare Safety Network reporting criteria,^21^ which include superficial incisional SSIs within 30 days and deep incisional or organ or space SSIs within 90 days of fracture surgery. Because the management of some fractures may require more than 1 operative procedure as part of an intentionally staged surgical plan (eg, multiple irrigation and debridements, wound closures, temporary stabilization surgical procedures, or definitive fixation surgery), the primary outcome will include any SSI event from the date of fracture to the end of the 90-day postoperative surveillance periods from the definitive fracture management surgery. For participants with multiple fracture regions, the date of the definitive fracture management surgery will be matched to the fracture region with the SSI.

The sensitivity and robustness of the primary outcome will also be assessed using 2 exploratory definitions of SSI: (1) fracture-related infection, defined by the confirmatory criteria outlined in a consensus definition,^22^ and (2) the CDC criteria expanded to include all SSIs that occur within 12 months of fracture. These outcomes are considered exploratory because larger validation studies of their performance do not currently exist, to our knowledge; however, these alternative SSI definitions were included because the standard CDC criteria have been criticized for failing to adequately account for the complexities of infections in traumatic fractures.^22,23^ All reported SSIs will be reviewed by a blinded adjudication committee.

### Secondary Outcome

The secondary outcome is unplanned fracture-related reoperation within 12 months of the fracture(s). This outcome has been used in previous fracture trials and is defined as any unplanned surgery that occurred from the time of injury to 12 months after fracture that is associated with an infection at the operative site or contiguous to it, a wound healing problem, or a fracture healing problem (ie, delayed union or nonunion). Common examples include any unplanned (1) irrigation and debridement of surgical incisions or open fracture wounds due to infections or wound healing problems, (2) revision wound closure for dehiscence, (3) soft-tissue coverage procedure for infected or necrotic wounds, (4) union or nonunion surgery (such as bone grafting or implant exchange) delayed by fracture, and (5) reoperation for hardware or prosthesis failure due to infection or bone healing problems. The members of the adjudication committee will review all reported reoperations.

### Data Collection and Participant Follow-up

Surgical site infections and unplanned fracture-related reoperations will be identified at the time of diagnosis or occurrence and/or during each participant’s clinical assessment and medical record review that will occur during his or her initial hospital stay or routine outpatient clinic visit. Detailed information on reported SSIs and reoperations will be collected. In cases in which the participant does not return to the clinic, study personnel will contact the participant by telephone, text, email, or standard mail at 6 weeks, 3 months, 6 months, 9 months, and 12 months after the fracture. Outside medical records will be obtained as necessary. Participants will not be withdrawn from the study if they do not adhere to the study protocol.

### Data Management and Data Monitoring

Clinical site personnel will enter all data into the REDCap Cloud electronic data capture system (nPhase Inc). The REDCap Cloud system uses several mechanisms for checking data at the time of entry including skip logic, range checks, and data-type checks. Personnel at the Methods Center will query all missing, implausible, or inconsistent data. Methods Center personnel will also perform in-person and remote monitoring of each participating clinical site.

### Safety Monitoring

A data and safety monitoring committee will oversee the safety of the trial participants and the overall conduct of the trial. The members of the data and safety monitoring committee will include a minimum of 2 orthopedic surgeons, an infectious diseases expert, and a biostatistician. One orthopedic surgeon will act as the chair of the committee. The data and safety monitoring committee will be responsible for safeguarding the interests of study participants, assessing the safety and efficacy of study procedures, and monitoring the overall conduct of the study. They will advise the principal investigators of any concerns related to participant safety and trial conduct and may make recommendations regarding the trial.

### Statistical Plan

#### Sample Size Determination

In each fracture cohort, the analyses will compare the effectiveness of the iodophor solution with the effectiveness of the chlorhexidine solution. Separate sample size estimates for each fracture population were calculated to facilitate the primary comparison between proportions of patients with SSIs in each treatment group. It is expected that this estimate will also provide adequate power for the secondary outcome (unplanned fracture-related reoperation) because a meaningful effect size for the reoperation outcome is expected to be similar to the SSI estimates. In addition, the baseline risk of unplanned reoperations in both fracture populations is expected to be greater than the risk of SSIs.^3^

Assuming an ITT principle for the analysis, we calculated the sample size based on a cluster-crossover design with the cluster as the unit of randomization and the patient as the unit of analysis. Simulation methods were used to obtain empirical power calculations based on a feasible number of recruiting clusters and the expected number of participants within the open and closed fracture populations.^24^ The simulation estimates are designed to detect a difference between the treatment groups, accounting for between-hospital variability inherent in a cluster-crossover trial design.

We have estimated that the chlorhexidine group will experience an SSI incidence of 12.5% in the open fracture population and a 3.5% incidence within the closed fracture population.^2,3^ Compared with the chlorhexidine solution, we have assumed the iodophor solutions will achieve a 0.65 risk ratio for SSIs and unplanned fracture-related reoperations in each fracture population.^8^ This effect was deemed more conservative than the data reported by Swenson et al^7^ and was consistent with feasible recruitment goals.

We have based our sample size assumptions on a single crossover, 2-period design to ensure the most conservative sample size estimate. Recent simulation data suggest that increasing the number of period crossovers can increase the statistical power of a given sample size.^25^ The initial power estimate assumed 10 recruiting clusters, a 10% loss to follow-up rate,^3^ no between-period variance, and between-cluster variance of 0.095 observed in the FLOW trial. Based on enrollment of a minimum of 1540 patients with open fractures (for the Aqueous-PREP trial and the PREPARE open fracture cohort) and 6280 patients with closed lower extremity and pelvic fractures (PREPARE closed fracture cohort), greater than 80% power would be achieved for each fracture population. Subsequent to the initial power calculations, the early trial experience demonstrated a need to increase the number of clusters to obtain a feasible recruitment pace. As a result, a minimum of 12 Aqueous-PREP clusters and 18 PREPARE clusters will enroll participants. The increase in clusters results in a marginal increase in power (approximately 2%). Table 3 outlines the summary of the initial sample size assumptions that yield 80% or more power to detect differences between the treatments within each fracture cohort.

**Table 3.** Sample Size Estimates

Iodine risk ratio	Iodine odds ratio	Sample size	Sample size increased by 10%
**Aqueous-PREP and the PREPARE open fracture cohort**^a^			
10.0% Baseline SSI risk			
0.62	0.59	1600	1760
0.65	0.63	1960	2100
0.67	0.65	2200	2420
0.70	0.68	2600	2860
12.5% Baseline SSI risk			
0.62	0.59	1300	1440
0.65	0.62	1400	1540
0.67	0.64	1600	1760
0.70	0.67	1800	1980
14.0% Baseline SSI risk			
0.62	0.58	1200	1320
0.65	0.61	1300	1440
0.67	0.64	1500	1660
0.70	0.67	1800	1980
**PREPARE closed fracture cohort**^**a**^			
2.0% Baseline SSI risk			
0.62	0.62	8200	9020
0.65	0.65	10 000	11 000
0.67	0.67	11 400	12 540
3.5% Baseline SSI risk			
0.62	0.61	4700	5170
0.65	0.64	5700	6280
0.67	0.66	6600	7260
5.0% Baseline SSI risk			
0.62	0.61	3300	3640
0.65	0.64	4100	4520
0.67	0.67	4300	4740

Abbreviations: Aqueous-PREP, Pragmatic Randomized Trial Evaluating Pre-Operative Aqueous and Antiseptic Skin Solution in Open Fractures; PREPARE, Pragmatic Randomized Trial Evaluating Pre-Operative Alcohol Skin Solutions in Fractured Extremities; SSI, surgical site infection.

^a^ Initial assumptions for sample size estimates: between-cluster intraclass correlation coefficient = 0.028; between-cluster variance = 0.095; between-period variance = 0; 10 clusters; 2 periods; α = .05.

### Overview of the Statistical Analysis Plan

A detailed statistical analysis plan will be published prior to the completion of the trials. In brief, the analyses will be conducted independently for each of the 3 fracture populations (see statistical analysis plan in Supplement 3). For each population, the analyses and reporting of the results will follow the CONSORT guidelines for reporting of both pragmatic trials^26^ and cluster randomized trials.^27^ The process of participant enrollment and flow throughout the study will be summarized using a flow diagram. Participant demographic characteristics and baseline outcome variables will be summarized using descriptive summary measures expressed as mean (SD) or median (interquartile range) values for continuous variables depending on the distribution and as number and percentage for categorical variables.^28^

An ITT principle will be adopted to analyze all outcomes. The primary analysis will compare the treatment groups using the SSI outcome. This analysis will be repeated using the exploratory outcomes (ie, fracture-related infection and CDC definition within 12 months of injury) to determine whether the study conclusions are sensitive to alternative definitions of SSI. The secondary analysis will compare the outcomes of unplanned fracture-related reoperations. The secondary comparison will be conducted in accordance with best practice guidelines for secondary analyses. Each subgroup analysis will be performed by comparing the effect estimates in both groups (interaction effect). The subgroup analyses will be approached and reported in accordance with best practices and guidelines for subgroup analyses.^12,29,30,31^ For all analyses, the results will be expressed as a relative measure of effect (odds ratios, risk ratios, or hazard ratios) and the corresponding 2-sided 95% CI. All hierarchical models will consider potential between-cluster and between-period variance. Missing data will be assumed to be missing at random and will be handled using multiple imputation.^32,33^ Additional sensitivity analyses are also planned to assesss the sensitivity or robustness of the findings to key assumptions. These analyses may explore the effects of alternative analysis models, alternative missing data approaches, balancing prognostic imbalance, as-treated analyses, variability in cointerventions, and alternative definitions of SSI.

## Discussion

Master protocols describe a single overarching protocol designed to answer several research questions by involving 1 or more interventions in multiple diseases or a single disease with multiple interventions. The PREP-IT master protocol allows for increased efficiency through shared trial infrastructure and study design components. To our knowledge, a master protocol has not been used to conduct comparative effectiveness research in orthopedic surgery.

The decision to establish a master protocol and conduct separate trials for the aqueous-based and alcohol-based solutions was made to accommodate multiple funders and allow small, but clinically important, protocol differences in the target populations. Specifically, the investigators were able to secure separate funding for Aqueous-PREP and PREPARE from different funding agencies by proposing the trial that best fit within the funder’s mandate and budget. Clinically, by using a master protocol to guide multiple trials, it allowed the study solutions to be compared in the most clinically relevant study populations. For example, the investigators had strong equipoise for comparing the effectiveness of iodophors with the effectivenss of chlorhexidine, as well as aqueous-based and alcohol-based solutions, for an open fracture; however, they believed that only alcohol-based solutions should be used for closed fracture repair. Therefore, a 4-group trial could have been possible for patients with open fractures but not for patients with closed fractures. Similarly, the incidence of SSIs among patients with closed fractures is much lower than among patients with open fractures and is further lower among patients with closed upper extremity fractures compared with patients with closed lower extremity fractures. As a result, the decision was made to enroll patients with only lower extremity and pelvic fractures within the closed fracture population to observe a higher incidence of study events and be more likely to be statistically powered to detect clinically important differences within a feasible study sample size.

**Figure 2.**
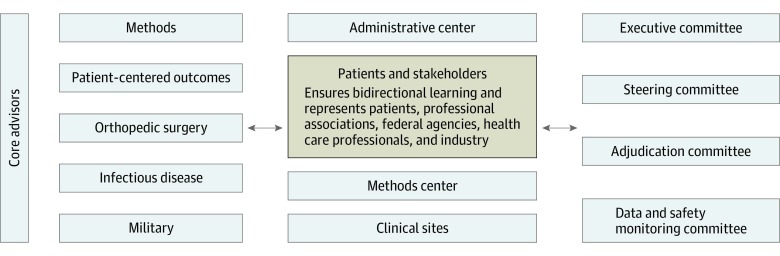
PREP-IT (Program of Randomized Trials to Evaluate Pre-operative Antiseptic Skin Solutions in Orthopaedic Trauma) Organization

In addition to the efficiencies of developing nearly identical protocol documents, study materials, and databases, the PREP-IT trials have benefited from a single trial infrastructure (Figure 2). The organizational structure is built with a strong emphasis on patient and stakeholder engagement, and nearly all trial decisions and activities involve bidirectional learning with these partners. The remainder of the trial governance is led by an executive committee composed of the principal investigators and a patient partner, a steering committee, a research methododology core, and multiple specialty cores.

## Conclusions

The PREP-IT master protocol provides a single framework for the conduct of 2 trials that will compare the effectiveness of the most common iodophor and chlorhexidine skin antiseptic solutions used in orthopedic fracture repair. Because prophylactic skin antisepsis is used prior to all surgical procedures and the application, cost, and availability of all study solutions are similar, the results of the PREP-IT trials are poised to bring about an immediate change in clinical practice.

## Notes

Supplement 1. **Aqueous-PREP Trial Protocol**

Supplement 2. **PREPARE Trial Protocol**

Supplement 3. **Statistical Analysis Plan**
