# Error in Article Information Section

**DOI:** 10.1001/jamanetworkopen.2020.21571

**Published:** 2020-09-08

In the Original Investigation titled “Effectiveness of Iodophor vs Chlorhexidine Solutions for Surgical Site Infections and Unplanned Reoperations for Patients Who Underwent Fracture Repair: The PREP-IT Master Protocol,”^1^ published April 7, 2020, the first name of a group member listed in the Article Information section was incorrectly given. The name “Mary Brownrigg” should have appeared as “Maggie Brownrigg.” This article has been corrected.^1^
