# Nonauthor Collaborator Names Added to a Supplement

**DOI:** 10.1001/jamanetworkopen.2021.17240

**Published:** 2021-06-16

The Original Investigation titled “Effectiveness of Iodophor vs Chlorhexidine Solutions for Surgical Site Infections and Unplanned Reoperations for Patients Who Underwent Fracture Repair: The PREP-IT Master Protocol”^1^ that was published on April 7, 2020, has been corrected to include the nonauthor collaborator names in a supplement.
