# Supplementary material for: Effectiveness of Iodophor vs Chlorhexidine Solutions for Surgical Site Infections and Unplanned Reoperations for Patients Who Underwent Fracture Repair: The PREP-IT Master Protocol
Source: JAMA Netw Open. 2020 Apr 7;3(4):e202215. doi: 10.1001/jamanetworkopen.2020.2215 (PMC7139274; doi:10.1001/jamanetworkopen.2020.2215)
Supplement: Supplement 1. — Aqueous-PREP Trial Protocol [file jamanetwopen-3-e202215-s001.pdf]

PREP-IT
A Program of Randomized trials to Evaluate Pre-operative antiseptic skin solutions In orthopaedic Trauma

Aqueous-PREP: A Pragmatic Randomized trial Evaluating Pre-operative aqueous antiseptic skin solutions in open fractures

Aqueous-PREP PROTOCOL

Version: 2.1

Date: 04-Nov-2019

This study is funded by the US Department of Defense, Physician Services Incorporated, and McMaster University Surgical Associates.

The Aqueous-PREP trial is part of the PREP-IT research program. The protocol is the confidential intellectual property of the Principal Investigators and PREP-IT Steering Committee, and the protocol cannot be used in any form without the expressed written permission of the Principal Investigators.

Reviewed and Approved by:		
Dr. Gerard Slobogean (Principal Investigator)	***Signature:***	***Date:*** 04-Nov-2019
Dr. Sheila Sprague (Principal Investigator)	***Signature:***	***Date:*** 04-Nov-2019
Dr. Mohit Bhandari (Principal Investigator)	***Signature:***	***Date:*** 04-Nov-2019

TABLE OF CONTENTS

LIST OF ABBREVIATIONS 4

STUDY SUMMARY 5

1.0 INTRODUCTION 7

1.1 Open Fractures 7
1.2 Prevention of Infection 7
1.3 Rationale for Pre-Operative Antiseptic Skin Solution Prophylaxis 7
1.4 Extrapolating Evidence from Other Surgical Disciplines to Fracture Surgery is Problematic 8
1.5 Why Iodophor Skin Preparations May Reduce Open Fracture SSI 9
1.6 Why Iodophor Skin Preparations May Reduce Open Fracture Reoperations 9
1.7 Lack of Surgeon Consensus 10

2.0 STUDY OBJECTIVES AND HYPOTHESES 10

2.1 Study Objectives and Hypotheses 10
2.2 Subgroup Objectives 11

3.0 TRIAL DESIGN 11

3.1 Summary 11
3.2 Pragmatic-Explanatory Continuum 12

4.0 METHODS 13

4.1 Study Setting, Cluster Eligibility, and Selection of Clusters 13
4.2 Eligibility Criteria 14
4.3 Recruitment Strategy 15
4.3.1 Patient Screening and Consent 15
4.3.2 Enrollment Sampling Plan 16
4.4 Randomization Methods 16
4.5 Blinding 17
4.6 Description of the Interventions 17
4.6.1 Initial Run-In Phase 17
4.6.2 First Intervention Phase 17
4.6.3 Second Intervention Phase 18
4.6.4 Special Considerations for Ongoing Treatment Crossovers 18
4.6.5 Evaluation of Site Performance and Removal of Clinical Sites 18
4.6.6 Application of Pre-Operative Antiseptic Skin Solutions 19
4.6.7 Povidone-Iodine Treatment 19
4.6.8 Chlorhexidine Gluconate Treatment 20
4.7 Perioperative Co-Interventions 20
4.8 Outcome Measures 20
4.8.1 Primary Outcome 20
4.8.2 Secondary Outcome 23
4.8.3 Exploratory Outcomes 23
4.8.4 Data Collection and Participant Follow-up 24

5.0 STATISTICAL PLAN 25

5.1 Sample Size Determination 25
5.2 Statistical Methods 26
5.2.1 Analysis Plan Overview 26
5.2.2 Analysis of the Study Outcomes 27
5.2.3 Subgroup Analyses 27
5.2.4 Sensitivity Analyses 28
5.2.5 Interim Analysis 30
6.0 DATA MANAGEMENT 30
6.1 Case Report Forms and Data Transmission 30
6.2 Data Integrity 30
7.0 ETHICS AND DISSEMINATION 30
7.1 Research Ethics Approval 30
7.2 Consent 30
7.3 Confidentiality 32
7.4 Protocol Amendments 32
7.5 Adverse Event Reporting and Definitions 32
7.5.1 Serious Adverse Event (SAE) 32
7.5.2 Unanticipated Problems Resulting in Risk to Participant or Others 33
7.5.3 Clinical Site Reporting: Notifying the Methods Center 33
7.5.4 Clinical Site Reporting – IRB and REB 33
7.5.5 Safety Monitoring 33
7.6 Dissemination Policy 33
8.0 DEPARTMENT OF DEFENSE REPORTING REQUIREMENTS 34
9.0 SUB-STUDY: PATIENT EXPERIENCES IN THE AQUEOUS-PREP AND PREPARE TRIALS 34
9.1 Introduction 34
9.2 Rationale and Objectives 35
9.3 Sub-Study Design 35
9.4 Survey Participants and Distribution 36
9.5 Data Entry 36
9.6 Sample Size 36
9.7 Data Analysis 36
9.8 Anticipated Implications of Results 36
10.0 REFERENCES 38

LIST OF ABBREVIATIONS

Abbreviation	Explanation
Aqueous-PREP	A Pragmatic Randomized trial Evaluating Pre-operative aqueous antiseptic skin solutions in open fractures
CDC	Centers for Disease Control and Prevention
CEO	Center for Evidence-Based Orthopaedics
CHG	Chlorhexidine gluconate
CI	Confidence interval
CRF	Case report form
EDC	Electronic data capture
FDA	Food and Drug Administration
FLOW	Fluid Lavage of Open Wounds trial
FRI	Fracture-related infection
GEE	Generalized estimating equations
HRPO	Human Research Protection Office
ITT	Intention-to-treat
IRB	Institutional Review Board
ORP	Office of Research Protections
REB	Research Ethics Board
SAE	Serious adverse event
SSI	Surgical site infection
USAMRMC	United States Army Medical Research and Materiel Command

STUDY SUMMARY

**Methodology**	Cluster randomized crossover design.
**Coordinating Center**	This study will be centrally coordinated by the Methods Center at the Center for Evidence-Based Orthopaedics (CEO), McMaster University, Hamilton, Ontario and by the Administrative Center within the Department of Orthopaedics at the University of Maryland, R Adams Cowley Shock Trauma Center, Baltimore, Maryland.
**Clinical Sites**	At least 12 clinical sites. Additional clinical sites will be included or removed as needed.
**Background**	The prevention of infection is the single most important goal influencing peri-operative care of patients with open fractures. Standard practice in the management of open fractures includes sterile technique and pre-operative skin preparation with an antiseptic solution. The available solutions kill bacteria and decrease the quantity of native skin flora, thereby decreasing surgical site infection (SSI).[1–4] While there is extensive guidance on specific procedures for prophylactic antibiotic use and standards for sterile technique, the evidence regarding the choice of antiseptic skin preparation solution is very limited for open fracture surgery.
**Objectives**	The overall objective is to compare the effectiveness of aqueous pre-operative antiseptic skin preparation with 10% povidone-iodine versus 4% chlorhexidine gluconate (CHG) for the management of open fractures. A ranked order for assessing effectiveness will be used with *surgical site infection (SSI)* as the primary comparison and *unplanned fracture-related reoperations* as the secondary comparison.
**Subgroup Objectives**	We will explore if the pre-operative antiseptic skin solutions have different magnitudes of effect on SSI within three clinically important open fracture subgroups: severity of open fracture, location of fracture, and severity of wound contamination.
**Diagnosis and Main Inclusion Criteria**	All patients 18 years of age or older who present to a recruiting hospital for treatment of an open fracture(s) of the appendicular skeleton will be screened for participation within 3 weeks of their fracture. Eligible patients must receive surgical debridement of their open fracture wound(s) within 72 hours of their injury and the open fracture(s) must be managed definitively with a surgical implant (e.g., internal fixation, external fixation, joint prosthesis, etc.).

**Treatment Groups**	The Aqueous-PREP trial will compare two common iodophor and chlorhexidine based pre-operative antiseptic skin solutions used during open fracture surgery: 1) *Povidone-iodine:* 10% povidone-iodine (1% free iodine) in purified water and 2) *CHG:* 4% chlorhexidine gluconate in purified water.
**Randomization**	Treatment allocation will be determined using a cluster-randomized crossover trial design. The order of treatment allocation for each orthopaedic practice will be randomly assigned using a computer-generated randomization table. Each site will start with the initially allocated study solution and eventually crossover to the other solution for their second recruitment period. This process of alternating treatments will repeat approximately every 2 months as dictated by the initial randomization.
**Study Outcomes**	The primary outcome is SSI, guided by the Centers for Disease Control and Prevention's (CDC) National Healthcare Safety Network reporting criteria,[5] which includes superficial incisional SSI within 30 days and deep incisional or organ/space SSI within 90 days of definitive fracture management surgery. The secondary outcome is the occurrence of an unplanned fracture-related reoperation within 12 months of the fracture. Alternative definitions of SSI, including the confirmatory criteria for Fracture-Related Infection (FRI) and the CDC criteria within 1 year of injury will be used for sensitivity analyses of the primary comparison. All study outcomes will be adjudicated by a blinded committee using clinical notes and radiographs.
**Follow-Up**	Study participants will be followed at 6 weeks, 3 months, 6 months, 9 months, and 12 months from their fracture.
**Sample Size**	A minimum of 1,540 participants with open fractures.
**Significance**	SSIs are often devastating complications for open fracture patients because of the resultant reoperations, adverse events from antibiotic courses, and fracture healing difficulties. Given the severity of open fractures, maximizing the effectiveness of current prophylactic procedures is essential. The Aqueous-PREP trial will provide necessary evidence to guide the prevention of SSIs in open fractures, and the trial is poised to have a significant impact on the care and outcomes of open extremity fracture patients.

1.0 INTRODUCTION

1.1 Open Fractures

Open fractures represent some of the most severe musculoskeletal injuries.[6] Due to their high-energy mechanisms, these extremity fractures are accompanied by soft-tissue injuries that contribute to unacceptably poor outcomes. The Fluid Lavage of Open Wounds (FLOW) trial of 2,447 open fracture patients reported a 13.2% incidence of open fracture-related reoperations;[7] however, these study events were even more common in open wounds with severe contamination (29.8%, 95% confidence interval (CI): 22.6–38.1%) and in patients with higher grades of injury (Gustilo-Anderson Type III: 18.0%, 95% CI: 15.6–20.7%). Ultimately, complications from open fractures lead to prolonged morbidity, loss of function, and potential limb loss.[8]

1.2 Prevention of Infection

The prevention of infection is the single most important goal influencing peri-operative care of patients with open fractures. Standard practice in the management of open fractures includes sterile technique and pre-operative skin cleaning with an antiseptic solution. The available solutions kill bacteria and decrease the quantity of native skin flora, thereby decreasing surgical site infection (SSI).[1–4] While there is extensive guidance on specific procedures for prophylactic antibiotic use and standards for sterile technique, the evidence regarding the choice of antiseptic skin preparation solution is very limited for open fracture surgery.

1.3 Rationale for Pre-Operative Antiseptic Skin Solution Prophylaxis

The most common skin preparation solutions include either an iodophor or chlorhexidine-based active ingredient, and are delivered in an alcohol or aqueous-based solution. Iodophors achieve effective antisepsis by penetrating the cell wall of microorganisms and disrupting critical protein and nucleic acid structures.[9] Iodophors are effective against most bacteria, but also may have broader-spectrum coverage of mycobacteria, viruses, and some spores compared to chlorhexidine gluconate (CHG).[9] CHG similarly achieves antimicrobial effects by penetrating the cell wall of microorganisms. This antimicrobial action allows CHG to be effective against most bacteria.[9]

The evidence guiding pre-operative antiseptic skin solution choice in fracture surgery is largely extrapolated from other surgical disciplines. In a randomized controlled trial involving 849 patients undergoing clean-contaminated abdominal, gynecologic, or urologic surgery, the use of 2% CHG in 70% isopropyl alcohol was compared to aqueous 10% povidone-iodine. The overall rate of 30-day SSI was significantly lower in the CHG-alcohol group than the povidone-iodine group (9.5% vs. 16.1%; P=0.004; relative risk, 0.59; 95% CI: 0.41–0.85). While this study demonstrated superior efficacy of CHG-alcohol compared to povidone-iodine, comparing an alcohol based solution to an aqueous solution creates uncertainty about whether the result observed occurred from the superiority of CHG over iodine, isopropyl alcohol over water, or a synergistic combination of CHG with alcohol.[1] In an effort to overcome the controversies associated with comparing CHG and iodine in different solutions, a more recent randomized controlled trial of 1,147 caesarean section patients allocated patients to 2% CHG with 70% isopropyl alcohol versus 8.3% povidone-iodine with 72.5% isopropyl alcohol. Similar to the previous randomized controlled trial, CHG proved more efficacious for reducing 30-day SSI

(4.0% in the CHG-alcohol group and 7.3% in the iodine-alcohol group; relative risk, 0.55; 95% CI: 0.34–0.90; P=0.02).[2]

While the evidence from the above two randomized controlled trials demonstrate decreased SSI from CHG solutions in clean-contaminated abdominal and genito-urinary surgery, a larger non-randomized trial reported opposite effectiveness results. Swenson *et al.,* completed a larger 3,209 patient pragmatic sequential implementation study, in which the use of the preoperative skin antiseptic solution was changed after six-month periods.[4] In this study, there were three treatment periods, each with approximately 1,000 general surgery patients undergoing elective and emergent cases. In the first period, patients received 7.5% povidone-iodine scrub, 70% isopropyl alcohol scrub, and 10% povidone-iodine skin paint. The second group received 2% CHG with 70% isopropyl alcohol (CHG group), and the third group received 0.7% iodine povacrylex in 74% isopropyl alcohol. Adjusted comparisons were performed using the intention to treat principle and an as-treated analysis. Lower SSI rates were seen in the povidone-iodine skin paint group (4.8%) and the iodine povacrylex in isopropyl alcohol group (4.8%), compared with the SSI rates in the 2% chlorhexidine and 70% isopropyl alcohol group (8.2%) (P< 0.05; povidone-iodine skin paint odds ratio: 0.56, 95% CI: 0.40–0.79).[4] While the results of the Swenson study contradict those of the smaller randomized controlled trials, this large pragmatic study further highlights that the choice of antiseptic skin solution affects SSIs, and data to select the best solution remains conflicting.

Considering the conflicting data, the most recent Cochrane systematic review comparing the efficacy of pre-operative antiseptic skin solutions for clean surgery concluded, "investment in at least one large trial (in terms of participants) is warranted to add definitive and hopefully conclusive data to the current evidence base. Ideally any future trial would evaluate the iodine-containing and chlorhexidine-containing solutions relevant to current practice…"[10] The Cochrane recommendation is a direct response to the limitations of the current available literature comparing antiseptic skin solutions. For orthopaedic fracture surgery, the impact of the treatment uncertainty is further magnified when considering the higher rates of SSIs among open fracture patients.

1.4 Extrapolating Evidence from Other Surgical Disciplines to Fracture Surgery is Problematic

With regards to orthopaedic patients, the inconsistent results leave the optimal antiseptic solution in doubt; in addition, results may differ across surgical settings. The risk of SSI is substantially greater in open fracture patients due to both the nature of the injury and the required surgery to fix broken bones. Furthermore, the emergent nature of fracture surgery means that patients are unable to undergo other prophylactic skin care, such as CHG bathing, which is rendered to elective cases to reduce SSI.

Most important, the contamination from the injury is a critical difference from elective abdominal or gynecologic surgery. Other differences include substantial soft tissue damage during the injury, the use of a tourniquet that decreases the blood flow to the limb (potentially increasing the risk of infection), and the additional risk of implanting metal fixation that can harbor bacteria. Swenson *et al.,* directly acknowledged that the studies performed in general surgery patients may not apply to other specialties, particularly orthopaedic surgery.[4] Even if one

wanted to directly apply the conflicting results outlined above to the care of open fractures, there are critical limitations in the sparse general surgery and obstetrical literature available.

The most significant limitation in the existing literature is the use of a 30-day endpoint for SSI in all three studies described above.[1,2,4] While this may be acceptable for identifying most SSIs that involve only the skin (superficial SSI), infections that occur deep to the muscle and around the bone (deep SSI and organ/space SSI) often present beyond 30-days post-injury and have significantly more morbidity and mortality than superficial SSI. This is a major limitation to the external validity of the previous studies' ability to guide fracture fixation practice. In the FLOW open fracture trial, nearly half the infection-related complications were identified between 30 and 90 days from injury.[7] Not only does the existing literature not extend follow-up during this period, it is plausible that the treatment effects of the antiseptic solutions behave differently for preventing deep or organ/space infections that often present between 30 and 90 days post-surgery. The need for longer follow-up is supported by a mandatory 90-day surveillance period for deep and organ/space SSIs according to the Centers for Disease Control and Prevention (CDC).[5] Therefore, the lack of directly applicable evidence, an overall paucity of good clinical evidence, and the inadequate duration of outcome follow-up mandate the need for a large, rigorous clinical trial in surgical preparation solutions in open fracture care.

<u>1.5 Why Iodophor Skin Preparations May Reduce Open Fracture SSI</u>

The only surgical skin preparation effectiveness data available for open fracture management come from the FLOW trial.[7] Secondary multivariable analyses of 2,447 patients with open fractures found that when compared to chlorhexidine solutions, iodophor-based skin antiseptic preparation solutions could be protective against complications (Adjusted Hazard Ratio 0.88, 95% CI: 0.69–1.12).[7] However, the wide CI suggests iodophor solutions may reduce the odds of infection by as much as 31% or increase it by as much as 12%, leaving its superiority as a fracture care surgical preparation solution unresolved.[7]

There are several chemical properties to suggest povidone-iodine may be more effective than CHG at preventing open fracture SSI.[9] Firstly, povidone-iodine has a broader spectrum of antimicrobial activity.[9] Secondly, many open fracture patients require repeat surgical debridement and therefore, these patients will receive multiple exposures to the pre-operative antiseptic solution. Extended use of povidone-iodine has not been associated with the selection of resistant bacterial strains, whereas bacterial resistance to chlorhexidine has been documented.[9,11,12] While the methods for detecting CHG resistance are challenging and its clinical significance remains uncertain, these early observations heighten interest in establishing the comparative effectiveness of iodophors versus CHG.

<u>1.6 Why Iodophor Skin Preparations May Reduce Open Fracture Reoperations</u>

While the primary rationale for using antiseptic skin preparation solutions is to reduce the risk of SSI, many fracture healing complications are associated with indolent infections. These low-grade infections typically do not exhibit clinical signs consistent with SSI. Instead, they present several months post-fracture fixation and are only detected from deep tissue samples collected during secondary surgeries to treat fractures that fail to heal (nonunion). Previous fracture non-union studies have identified an infectious etiology in 31–38% of cases.[13,14] Similarly, results from the FLOW trial suggest that 58% of the reoperation events were caused by fracture

nonunion or a hardware failure related to infection, wound-healing problem, or bone-healing problem (n= 188/323). In addition, among a series of 211 patients requiring reoperation for deep post-operative fracture infections, 40% of open fracture infections occurred beyond the 90-day surveillance period for SSI.[15] Therefore, given the rationale that povidone-iodine may be more effective in preventing SSI, it is clinically plausible that its use may also reduce unplanned open fracture reoperations.

1.7 Lack of Surgeon Consensus

The FLOW trial demonstrated a clear divide among orthopaedic surgeons regarding their choice to use the two most common antiseptic solutions during open fracture fixation surgery.[7] Iodophor solutions were used in 54% of the surgeries performed, while 41% were performed using chlorhexidine solutions. The remaining surgeons either used both iodophor and chlorhexidine (4%), or alcohol with no iodophor or chlorhexidine (1%).[7] Building upon the lack of consensus among orthopaedic surgeons participating in the FLOW trial, our research team conducted an internet-based survey and several interviews with orthopaedic surgeons to understand the reasons for the lack of consensus in the use of surgical preparation solutions. Similar to the observations of the FLOW trial, there was nearly an equal split between the use of iodophor and chlorhexidine solutions. More insight was gained in interviews with the surgeons. Three main drivers for surgeon decision-making were identified: 1) they continued to use the antiseptic solution shown to them during their surgical training, 2) they used the solution recommended by their hospital, or 3) they felt the tissue toxicity was less with their chosen solution. No surgeon could cite a clinical study that helped guide their decision, despite all surgeons indicating they believed the antiseptic solution was important for reducing their patient's risk of SSI. Limited consensus among surgeons reflects a lack of compelling evidence on the optimal approaches to surgical skin preparation, further vindicating the need for a large definitive trial.

The *Aqueous-PREP trial, A **P**ragmatic **R**andomized trial **E**valuating **P**re-operative aqueous antiseptic skin solutions in open fractures,* will address these gaps in the literature.

2.0 STUDY OBJECTIVES AND HYPOTHESES

2.1 Study Objectives and Hypotheses

The overarching objective of this trial is to compare the effectiveness of an aqueous pre-operative antiseptic skin preparation with 10% povidone-iodine versus 4% CHG for the management of open fractures. A ranked order for assessing effectiveness will be used, with *surgical site infection (SSI)* as the primary comparison (primary objective) and *unplanned fracture-related reoperations* as the secondary comparison (secondary objective). While previous randomized controlled trials in general surgery and gynecology demonstrated superior efficacy of chlorhexidine-alcohol solutions to reduce SSIs,[1,2] results from larger populations of general surgery patients and the recently completed FLOW trial[7] suggest iodophor-based solutions could be more effective than chlorhexidine in open fracture patients. Therefore, we hypothesize that aqueous solutions of 10% povidone-iodine will be more effective than aqueous 4% CHG to reduce 90-day SSIs or unplanned fracture-related reoperations within one year of injury.

2.2 Subgroup Objectives

The Aqueous-PREP trial will also explore the possibility of differential treatment effects of the pre-operative antiseptic skin solutions among clinically important open fracture subgroups. Subgroups will be defined by: i) the severity of open fracture (Gustilo-Anderson type I or II versus III);[8] ii) upper extremity versus lower extremity open fractures; and iii) severity of wound contamination. High-grade soft tissue injury (Gustilo-Anderson type III), lower extremity open fractures, and moderate/severe wound contamination are established predictors of SSI and reoperations from the FLOW trial.[16] In addition, there are known differences in patients' skin flora based on anatomic region of injury. As a result, it is likely that the study interventions may be more effective in certain subgroups. Due to its broader spectrum of antimicrobial activity, the increased effectiveness observed by Swenson *et al.*, and the possible benefits observed in the FLOW trial, we hypothesize that 10% povidone-iodine antiseptic skin solution will be associated with a larger reduction in odds for SSI and reoperation in open fracture patients with worse fracture severity, lower extremity fractures, and more severely contaminated wounds.

3.0 TRIAL DESIGN

3.1 Summary

This study is a multi-center pragmatic cluster randomized crossover trial of a minimum of 1,540 participants with open extremity fractures requiring surgical management. The unit of randomization is the orthopaedic practices within clinical sites (clusters), with individual patients being the unit of analysis. Recruitment for each treatment group will be performed in multiple iterations of approximately two-month periods. Each orthopaedic practice will initially be randomized to use one of two pre-operative aqueous surgical skin preparation solutions (10% povidone-iodine or 4% CHG) for open fracture surgeries at their institution (**Figure 1**). Upon completion of the two-month period, each orthopaedic practice will crossover to the alternative treatment allocation and complete another two-month recruitment period. This process of alternating treatment periods (crossovers) will continue until the minimum sample size is achieved and the study's budgeted recruitment duration is completed. Upon completion of recruitment, it is expected that each orthopaedic practice will enroll a minimum of 77 patients per treatment, and that most clinical sites will exceed this minimum recruitment goal. Clinical site personnel will screen potential patients for eligibility, and if eligible, they will be invited to participate in the trial. Study participants will be assessed at regular intervals in the one year following their fracture. The primary outcome will include any SSI event from the time of open fracture to the end of the 30- and 90-day post-operative periods from their definitive fracture management surgery. The secondary outcome will include unplanned fracture-related reoperations that occur within one-year of their fracture. A blinded Adjudication Committee will review SSIs and unplanned fracture-related reoperations to confirm that they meet the criteria for being a study event.

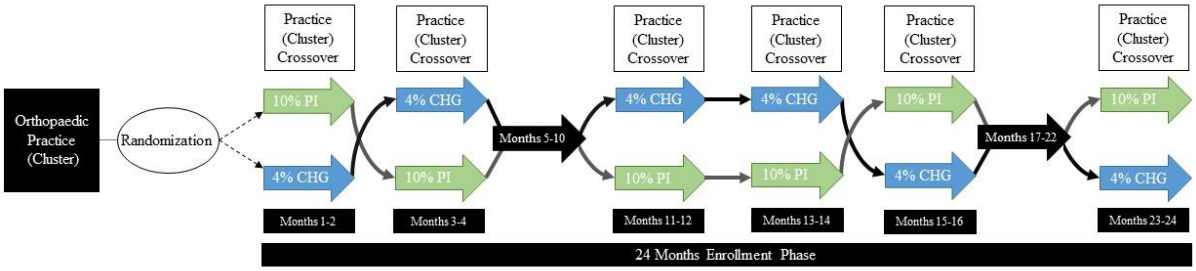

Figure 1: Randomized Treatment Allocation, Cluster Crossover, and Recruitment

3.2 Pragmatic-Explanatory Continuum

In accordance with recommended methodology standards, we have used the PRagmatic-Explanatory Continuum Indicator Summary (PRECIS-2) toolkit to evaluate the Aqueous-PREP trial design decisions to determine whether these decisions will lead to a study that answers, "Does this intervention work under usual conditions?" (pragmatic) versus "Can this intervention work under ideal conditions?" (explanatory). The PRECIS-2 tool uses a 5-point Likert scale in 9 domains to evaluate the continuum of design choices. A domain score of 5 indicates "very pragmatic," while a score of 1 suggests "very explanatory." **Table 1** outlines the investigators' assessment of the trial design and the rationale for each assessed score and **Figure 2** displays the PRECIS-2 wheel.

Table 1: PRECIS-2 Score

Domain	Score	Rationale
Eligibility	5	Eligibility criteria are very broad and include all fracture patients that would be treated in all hospital environments.
Recruitment	5	Recruitment of all consenting fracture patients treated at each participating hospital will be performed.
Setting	4	Recruitment is occurring at multiple sites across the US and Canada; however, since most of the recruiting hospitals are regional referral centers the setting is "mostly pragmatic."
Organization	5	The interventions do not need an increase in providers or care delivery compared to the usual antiseptic care provided. For each antiseptic solution, a brief in-service training session will be provided to the clinical sites, as per any new product/procedure that is being introduced into an operating room.
Flexibility (delivery)	5	The interventions will be delivered in the usual care manner with no advice on allowed co-interventions or strict protocols to ensure compliance.
Flexibility (adherence)	-	This section is left blank according to PRECIS-2 guidance because the intervention is provided prior to patient consent and individual patient compliance is not an issue. If provider adherence is considered, the study design is rather pragmatic (4) because there will be limited encouragement to follow the manufacturer's directions for use, other than periodic newsletters, investigator meetings, and possible provider survey during the recruitment period.
Follow-up	5	All study follow-up is consistent with usual care.
Primary outcome	5	The outcome has been validated by patients as being very relevant to the study participants and it does not require specialized expertise beyond the treating physician for diagnosis.
Primary analysis	5	All available study data will be used for analysis following the intention to treat principle.

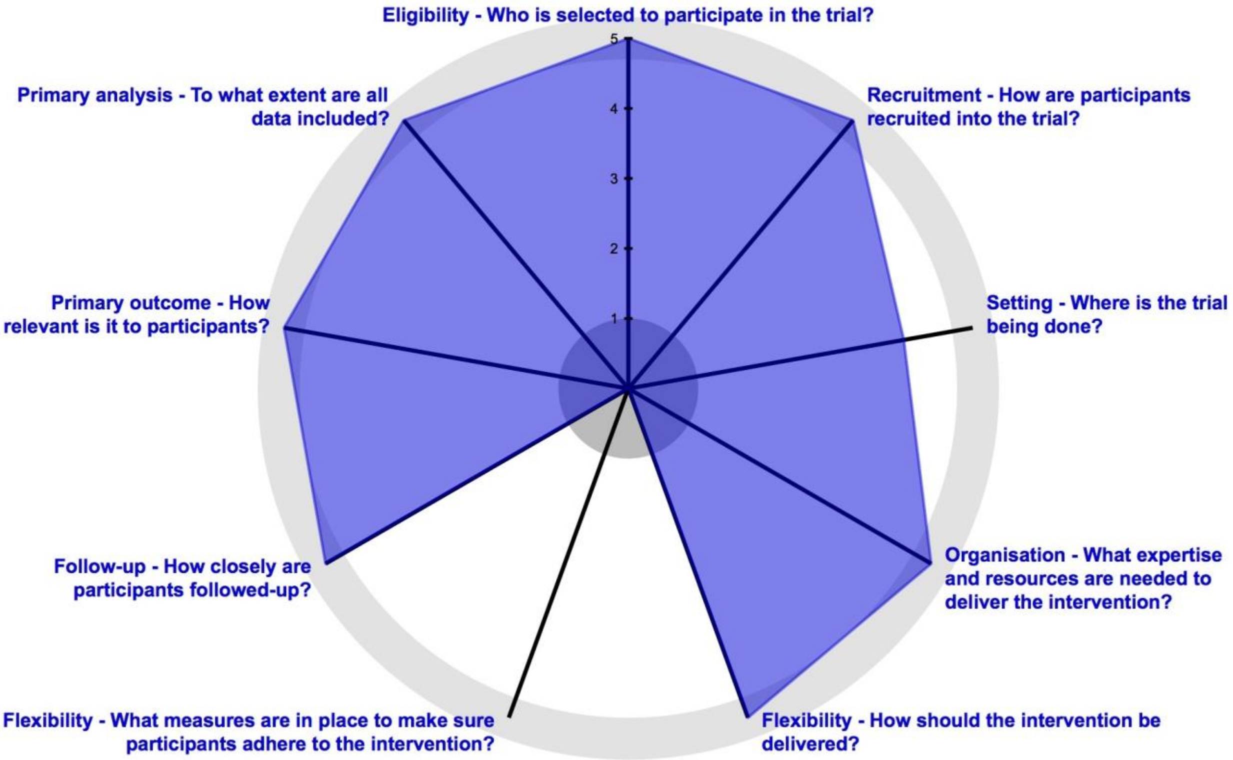

Figure 2: PRECIS-2 Wheel

4.0 METHODS

4.1 Study Setting, Cluster Eligibility, and Selection of Clusters

This study will be coordinated by the Methods Center at the Center for Evidence-Based Orthopaedics (CEO), McMaster University, Hamilton, Ontario and by the Administrative Center within the Department of Orthopaedics at the University of Maryland School of Medicine, R Adams Cowley Shock Trauma Center, Baltimore, Maryland. Patients will be enrolled from at least 12 clinical sites. Clusters (orthopaedic practices within clinical sites) will be carefully screened prior to participation in the Aqueous-PREP study. Clinical site inclusion criteria are: 1) adequate research personnel infrastructure to manage the study; 2) adequate open fracture volume to complete enrollment within the study timeline (i.e., a minimum of 77 open fractures per year); 3) commitment from all or most orthopaedic surgeons to participate in the trial; and 4) ability to use the two aqueous-based preparation solutions. The exclusion criteria are: 1) lack of interest in the trial; 2) anticipated challenges with complying with the protocol; 3) conflicting studies, in the judgment of the Principal Investigators, that would inhibit patient participation; and 4) budgeting or contract constraints.

The screening process will begin with potential clinical sites completing a feasibility questionnaire that asks about research experience and infrastructure, open fracture volume, current practice patterns, and interest in participating in the trial. Clinical sites that meet the eligibility criteria at this stage will be invited to participate in a series of teleconferences to review study and clinical logistics in detail with members of the study team. The Principal

Investigators and study personnel will further vet the clinical sites during these calls and will ask about hospital and patient demographics to ensure that a variety of fracture patient populations and referral patterns, ranging from large urban trauma centers to rural community hospitals, are included in the Aqueous-PREP study. Study personnel will document reasons for clinical site ineligibility. Upon selection, clinical sites will be asked to complete a questionnaire that will detail current surgeon preferences and practices for pre-operative surgical preparation techniques and co-interventions known to influence the incidence of SSIs (see Section 4.7).

4.2 Eligibility Criteria

Broad eligibility criteria will be used to increase the generalizability of the trial.

The inclusion criteria are:

1. Patients 18 years of age or older.
2. Open fracture of the appendicular skeleton.
3. Received or will receive definitive fracture treatment with a surgical implant(s) (e.g., internal fixation, external fixation, joint prosthesis, etc.).
4. Open fracture wound management that includes formal surgical debridement within 72 hours of their injury.
5. Will have all planned fracture care surgeries performed by a participating surgeon or delegate.
6. Informed consent obtained.
7. Patient enrolled within 3 weeks of their fracture.

The exclusion criteria are:

1. Patients that did not or will not receive the allocated pre-operative surgical preparation solution due to a medical contraindication.
2. Received previous surgical debridement or management of their open fracture at a non-participating hospital or clinic.
3. Open fracture managed outside of the participating orthopaedic service (e.g., hand fracture managed by plastic surgeon).
4. Chronic or acute infection at or near the fracture site at the time of initial fracture surgery.
5. Burns at the fracture site.
6. Incarceration.
7. Expected injury survival of less than 90 days.
8. Terminal illness with expected survival less than 90 days.
9. Previous enrollment in a PREP-IT trial.
10. Currently enrolled in a study that does not permit co-enrollment.
11. Unable to obtain informed consent due to language barriers.
12. Likely problems, in the judgment of study personnel, with maintaining follow-up with the patient.
13. Excluded due to sampling strategy.

Additional eligibility considerations:

1. Patients with multiple open fractures will be eligible for inclusion. Study personnel will collect data on up to three open fracture regions. In patients with more than three open fracture regions, the treating surgeon will determine the three most severe open fractures.

For each open fracture, the entire injured anatomic region will be included.[17] Therefore, if there are two open fractures that anatomically communicate, they will be considered within the same region (e.g. within the shoulder region, forearm, etc.). Similarly, adjacent closed fractures that anatomically communicate with the open fracture or are treated within the same surgical incision will be included. Common examples of these include forearm fractures, tibia/fibula fractures, and peri-articular fractures. The anatomic joint region, adjacent fractures, and contiguous wounds will be defined at the time of patient enrollment on the case report forms (CRFs).

2. All open fractures should be treated as per cluster randomization.
3. At the time of screening, patients who are in another study who meet eligibility criteria are to be included in the Aqueous-PREP trial unless the other trial does not permit co-enrollment.

4.3 Recruitment Strategy

4.3.1 Patient Screening and Consent

Patients 18 years of age or older who present to a recruiting hospital for treatment of an open fracture will be screened for participation within 3 weeks of their fracture. To screen patients presenting with an open fracture(s) for eligibility, designated study personnel at each clinical site will develop a patient enrollment plan. This plan will typically consist of daily participation in orthopaedic patient rounds and a review of daily listings of hospital admissions for patients with open fractures. Upon identification, the study personnel will screen the patient for eligibility and if eligible, approach them for informed consent. Study participants must be enrolled within 3 weeks of their fracture(s) and enrollment may take place at any time within this window. If the patient is unable to provide informed consent (e.g., due to their injury) at the time they were initially identified, informed consent may be delayed until they are able to provide informed consent. Alternatively, if the patient is unable to provide informed consent, informed consent may be obtained from their proxy, with consent obtained from the patient when/if the patient is able to provide consent. Allowing informed consent from a patient's proxy healthcare decision maker will reduce the risk of recruitment bias against the most severely injured patients. In addition, potentially eligible patients will be approached to participate in the trial, even if they did not receive the correct pre-operative antiseptic skin solution. This is consistent with the intention-to-treat principle (ITT) and is necessary to maintain the prognostic balance achieved during the cluster randomization. All screened patients will be classified as included, excluded, or missed. See **Table 2** below for the Schedule of Events.

Table 2: Schedule of Events

**Assessment**	**Visit 1:** Enrollment	**Visit 2:** 6 weeks post-fracture	**Visit 3:** 3 months post-fracture	**Visit 4:** 6 months post-fracture	**Visit 5:** 9 months post-fracture	**Visit 6:** 12 months post-fracture
Eligibility Screening	●					
Informed Consent	●					
Collection of Demographic and Fracture Characteristics Data	●					
Collection of	●					

Assessment	**Visit 1:** Enrollment	**Visit 2:** 6 weeks post-fracture	**Visit 3:** 3 months post-fracture	**Visit 4:** 6 months post-fracture	**Visit 5:** 9 months post-fracture	**Visit 6:** 12 months post-fracture
Surgical Data						
Collection of Peri-Operative Data	●					
Collection of Study Event (SSI) Data	●	●	●	●	●	●
Collection of Reoperation Data	●	●	●	●	●	●
Collection of SAE Data	●	●	●	●	●	●

Informed consent and enrollment must occur within the 3 weeks (21 days) from the patient's fracture (Day 0 is the date of the fracture).
Visits are to be completed at routine clinic visits. When necessary, visits may also be completed by telephone, text, email, standard mail, and/or a review of the participant's medical record.
Follow-up visit windows touch so that participants will always fall into a specific window. The windows are: 4 to 8 weeks (i.e., 28 to 56 days), 2 to 4.5 months (i.e., 57 to 137 days), 4.5 to 7.5 months (i.e., 138 to 228 days), 7.5 to 12 months (i.e., 229 to 365 days), and greater than 12 months (i.e., 366 to 730 days), respectively, from the participant's fracture.

4.3.2 Enrollment Sampling Plan

When the volume of eligible patients exceeds a participating site's ability to effectively enroll and follow all eligible patients, a sampling strategy may be implemented. A sampling strategy is available within the REDCap Cloud electronic data capture (EDC) system which will randomly determine whether an eligible patient should be approached for consent and inclusion in the study. The randomization software will use randomly selected block sizes consistent with the sampling ratio being used during the recruitment periods. Examples of potential random sampling strategies a site may use include:

1. For every three eligible patients, there will be one excluded eligible patient (3:1 ratio).
2. For every two eligible patients, there will be one excluded eligible patient (2:1 ratio).
3. For each eligible patient, there will be one excluded eligible patient (1:1 ratio).
4. For each eligible patient, there will be two excluded eligible patients (1:2 ratio).
5. For each eligible patient, there will be three excluded eligible patients (1:3 ratio).

The number of eligible patients approached for consent and inclusion in the study, and the number of eligible patients that are excluded due to a sampling strategy will be documented in the EDC system.

4.4 Randomization Methods

Treatment allocation will be determined using a cluster-randomized crossover trial design. The order of treatment allocation for each orthopaedic practice (cluster) will be randomly assigned using a computer-generated randomization table. Each site will start with the initially allocated study solution and crossover to the other solution for their second recruitment period. This process of alternating treatments will repeat approximately every 2 months as dictated by the initial randomization. For sites that enroll for more than 1 year, the order of treatment allocation

may be reversed after 12 months to ensure equal distribution of each treatment across each calendar month in the study's duration (**Figure 1**). Randomization will be completed by personnel at the CEO Methods Center at the onset of the trial. Personnel from the Methods Center will notify personnel at each participating clinical site of their treatment allocation order. This will allow each participating clinical site to begin preparing for the first run-in period.

4.5 Blinding

The orthopaedic team (including the study coordinators) cannot be blinded to the treatment allocation as the antiseptic solutions are visually distinguishable and these individuals need to lead the implementation of the cluster-crossover protocol at their clinical site. The Adjudication Committee Members and data analysts will be blinded to the study treatment. All interpretation of study results will initially be done in a blinded manner by developing two interpretations of the results. One interpretation will assume treatment A is povidone-iodine, the other interpretation will assume it is CHG. Once the data interpretations for each assumption are finalized, the data will be unblinded and the correct interpretation will be accepted.[18]

4.6 Description of the Interventions

4.6.1 Initial Run-In Phase

Prior to initiating patient recruitment, each clinical site will begin using their assigned pre-operative antiseptic skin solution for eligible open fracture surgeries (run-in period) to ensure that acceptable compliance is met before initiating participant enrollment. Acceptable compliance during the run-in phase will be defined as at least 15 eligible open fracture patients with >90% of eligible patients receiving the allocated antiseptic solution or a minimum of one month in duration. The run-in phase may be extended up to 3 months, as deemed necessary by the CEO Methods Center. Study personnel at each clinical site will document compliance with administering the allocated treatment during the run-in phase and submit this weekly to the CEO Methods Center. Specifically, the weekly reports will include the total number of eligible open fracture patients operated on, the proportion who received the assigned pre-operative antiseptic skin solution, and the proportion who did not receive the assigned pre-operative antiseptic skin solution along with details about the deviations (e.g., name of attending surgeon, solution used, rationale for not using the assigned pre-operative antiseptic skin solution). This portion of the study protocol is for quality assurance during the initial implementation of the trial procedures. Open fracture surgeries reviewed during the run-in phase will not be included in the trial. Similarly, these patients will not be approached for informed consent and no individual patient-level data will be submitted. CEO Methods Center personnel will review the weekly reports with each of the clinical sites and develop strategies, as needed, to ensure acceptable compliance during the run-in phase. This weekly communication will prevent any delays in transitioning to the participant enrollment phase.

4.6.2 First Intervention Phase

Once the initial run-in phase is completed, participant recruitment will begin with the clinical sites continuing to use the same pre-operative antiseptic skin solution for all eligible open fracture surgeries for a two-month period. Patients will receive the initially allocated treatment solution for all of their open fracture management surgeries, including repeat planned surgeries, even if a planned subsequent surgery occurs during a recruitment period using the non-allocated solution. Participating clusters will ideally be able to enroll a minimum of 77 open fracture

patients per treatment over the total study recruitment duration, and it is anticipated that most recruiting centers will exceed this minimum goal. Methods Center personnel will continue to monitor compliance with the assigned pre-operative antiseptic skin solution over the enrollment phase and work collaboratively with the clinical sites to minimize cases in which a patient receives the incorrect solution. These monitoring activities will coincide with site-specific procedures to maintain compliance for all patients, even those requiring multiple surgical procedures. If an open fracture requires multiple surgeries and the correct solution is not applied at each procedure, the patient will remain in the study and be analyzed using the allocated solution (ITT principle).

4.6.3 Second Intervention Phase

Once the first intervention phase is completed, each site will crossover to the opposite study solution. There will be no run-in phase for the second solution and each site will need to develop local procedures to ensure a successful crossover. Example procedures to minimize carry-forward of first solution into the second solution phase include: 1) removing the bottles of the first solution from the orthopaedic operating rooms; 2) changing study posters and notifications within the operating rooms; and 3) performing the crossover during the middle of the week to provide a few days' notice to the operating room staff and to avoid contamination of recent open fracture patients returning for repeat procedures (e.g., weekend admissions). The enrollment goals and procedures will mirror the first intervention phase. Methods Center personnel will continue to monitor compliance with the assigned pre-operative antiseptic skin solution over the enrollment phase and work collaboratively with the clinical sites to reduce the risk of contamination.

4.6.4 Special Considerations for Ongoing Treatment Crossovers

Treatment allocation will continue to alternate between the study solutions, as outlined above, for the remainder of study duration. Each intervention phase will be approximately 2 months in duration, as agreed upon by the local site and CEO Methods Center personnel. The duration may be modified to avoid crossovers on holidays, weekends, and other circumstances that could threaten a successful crossover. The expected recruitment duration for the trial is approximately 24 months; however, some sites may have a shorter total recruitment duration (e.g., a participating site who joins the trial later, high volume clinical sites, etc.). The two-month enrollment periods will help account for seasonal variability in SSI incidence and their associated infectious organisms,[19] as each crossover period will cover a season. In addition, for those clinical sites enrolling beyond 12 months, the distribution of recruitment periods for each solution may be seasonally matched by reversing the order of the alternating allocation after 12 months of recruitment.

4.6.5 Evaluation of Site Performance and Removal of Clinical Sites

After every two recruitment periods (approximately every four months), each site will be evaluated for continued participation in the trial. Sites with <90% of eligible patients receiving the allocated solution, differential adherence between study solutions, <95% follow-up of the primary outcome, <90% follow-up of the secondary outcome, incomplete data submission, or other threats to data quality or the validity of the study may be withdrawn from the trial. In the event a site is withdrawn, data collection will be completed for all enrolled participants and these data will be included in the final study analysis.

4.6.6 Application of Pre-Operative Antiseptic Skin Solutions

Each solution will be applied to the injured area in accordance with the Food and Drug Administration (FDA) and Health Canada approved manufacturer's directions for use. While the application and minimum drying time for both study solutions are very similar, local study personnel will provide standardized in-service (training) for orthopaedic surgeons, operating room technicians, and nurses at each participating hospital prior to the run-in phases for each of the two randomized interventions. This training should include reviewing the manufacturers' directions for use to help minimize incorrect application at clinical sites that may not routinely use both solutions. In addition, the manufacturers may also provide demonstration videos and posters for continued refresher training for each solution.

The study protocol will mandate the antiseptic skin solution to be used in each intervention phase (Sections 4.4, 4.6.1, 4.6.2, 4.6.3, and 4.6.4); however, the protocol will remain pragmatic to variability in other co-intervention steps performed during the entire pre-operative skin preparation process performed in the operating room. Based on individual surgeon preference, this often includes mechanically removing visible dirt or debris with a scrub brush, and/or cleaning the limb with isopropyl alcohol or antiseptic scrub solution. These additional skin preparation steps will be permitted provided that: 1) the final skin preparation step prior to surgical incision is the application of the allocated antiseptic solution; and, 2) participating surgeons continue to use the same skin preparation co-interventions in both intervention phases. Co-interventions that contain the opposite active ingredient from the current intervention phase (e.g., using a chlorhexidine scrub brush during the povidone-iodine intervention phase, or conversely, using a povidone-iodine scrub during the chlorhexidine intervention phase) should be avoided; however, deviations from this recommendation will be permitted in order to maintain pragmatic *flexibility of delivery* and reflect real-world clinical practice. The details of all operating room antiseptic co-interventions will be documented.

The pragmatic nature of the cluster-randomized design will reduce the risk of crossovers between treatment groups because all consecutive patients during each recruitment period will receive the same treatment. Similarly, open fracture patients that require multiple planned surgeries for their injury will receive the same antiseptic skin solution during each subsequent procedure. Methods Center personnel will work with each of the clinical sites to develop strategies for minimizing crossovers. For example, for patients enrolled within 14 days of the anticipated end of a recruitment period or patients requiring multiple surgeries, study personnel will develop local procedures to identify these patients as study participants, and indicate the patient's allocated antiseptic solution in the medical chart and CRFs.

4.6.7 Povidone-Iodine Treatment

The povidone-iodine solution will contain 10% povidone-iodine (1% free iodine) in purified water as the only active ingredient. Products that list other inactive ingredients, including alcohol, will be permitted. The brand of the solution will be left to the discretion of the clinical site, although Methods Center personnel will confirm that the chosen solution is acceptable. Acceptable brands include, but are not limited to, Betadine® [Purdue Products, L.P. Stamford, CT] and Scrub Care® [Cardinal Health, Dublin, OH]. Clinical site personnel will store and handle the povidone-iodine solution as per the manufacturers' recommendations. Operating

room personnel will apply the solution to the operative site as the final preoperative skin antisepsis preparation immediately prior to commencing surgical fixation. They will apply the solution as per manufacturer's directions for use (e.g., technique of application, duration of application, drying time, drying techniques, replacement of draping, etc.).

4.6.8 Chlorhexidine Gluconate Treatment

The CHG solution will contain 4% CHG in purified water as the only active ingredient. Products that list other inactive ingredients, including alcohol, will be permitted. The brand of the solution will be left to the discretion of the clinical site, although Methods Center personnel will confirm that the chosen solution is acceptable. Acceptable brands include, but are not limited to, Betasept® [Purdue Products, L.P. Stamford, CT] or Hibiclens® [Mölnlycke Health Care US LLC. Norcross, GA]. Clinical site personnel will store and handle the CHG solution as per the manufacturers' recommendations. Operating room personnel will apply the solution to the operative site as the final preoperative skin antisepsis preparation immediately prior to commencing surgical fixation. They will apply the solution as per manufacturer's directions (e.g., technique of application, duration of application, drying time, replacement of draping, etc.).

4.7 Perioperative Co-Interventions

To optimize the internal validity of the trial findings, key details of co-interventions known to influence the incidence of SSIs will be documented. Hospitals typically implement standard procedures to achieve quality process benchmarks designed to minimize SSIs. These benchmarks are outlined in several similar guidelines such as the Joint Commission's Surgical Care Improvement Project 10 Core Measures to prevent SSI, the Society for Healthcare Epidemiology of America compendium to prevent SSI, and prevention guides from the Institute for Healthcare Improvement and the Association of periOperative Registered Nurses. While these guidelines mandate core benchmark processes to minimize SSI, it is not practical or generalizable for the trial protocol to standardize the steps taken or co-interventions performed to achieve these core measures, since each participating hospital will already have their own implemented procedures. This is the primary rationale for the cluster-crossover design, in which each participating hospital will act as its own control for the effect of co-interventions. Therefore, four key approaches to account for and limit the potential differential application of co-interventions during the study periods will be performed: 1) study periods for each intervention are kept relatively short to improve the likelihood that newly implemented co-interventions will be equally distributed across both treatment solutions; 2) encourage participating hospitals not to make changes to their existing infection prevention interventions during the study periods; 3) document the co-interventions being used in the hospitals throughout the study periods; and 4) record any changes in co-interventions that do occur if mandated by a participating hospital's administration. To this end, a monitoring tool containing a list of commonly applied prophylactic co-interventions being used at the participating clinical sites will be completed every four months to document any changes to their infection prevention strategies during the study period.

4.8 Outcome Measures

4.8.1 Primary Outcome

The primary outcome is SSI, guided by the CDC's National Healthcare Safety Network reporting criteria (2017),[5] which includes superficial incisional SSI within 30 days and deep incisional or organ/space SSI within 90 days of fracture surgery (**Table 3**). Since the management of some

open fractures may have more than one operative procedure as part of an intentionally staged surgical plan (e.g., multiple irrigation and debridements, wound closures, temporary stabilization surgeries, definitive fixation surgery), the primary outcome will include any SSI event from the date of open fracture to the end of the 30- and 90-day post-operative surveillance periods from their definitive fracture management surgery. For participants with multiple open fracture regions, the date of the definitive fracture management surgery will be matched to the open fracture region with the SSI.

Table 3: CDC Surgical Site Infection Criteria

Outcome	Description
Superficial Incisional SSI	Date of event for infection occurs from the date of fracture to 30 days after the definitive fracture management surgery (where day 1 = the procedure date)<br>**AND**<br>involves only skin and subcutaneous tissue of the incision<br>**AND**<br>patient has at least one of the following:<br>a. purulent drainage from the superficial incision.<br>b. organisms identified from an aseptically-obtained specimen from the superficial incision or subcutaneous tissue by a culture or non-culture based microbiologic testing method which is performed for purposes of clinical diagnosis or treatment (e.g., not Active Surveillance Culture/Testing [ASC/AST]).<br>c. superficial incision that is deliberately opened by a surgeon, attending physician or other designee and culture or non-culture based testing is not performed.<br>**AND**<br>patient has at least one of the following signs or symptoms: pain or tenderness; localized swelling; erythema; or heat.<br>d. diagnosis of a superficial incisional SSI by the surgeon or attending physician or other designee.<br>The following do not qualify as criteria for meeting the definition of superficial SSI:<br>• Diagnosis/treatment of cellulitis (redness/warmth/swelling), by itself, does not meet criterion "d" for superficial incisional SSI. Conversely, an incision that is draining or that has organisms identified by culture or non-culture based testing is not considered a cellulitis.<br>• A stitch abscess alone (minimal inflammation and discharge confined to the points of suture penetration).<br>• A localized stab wound or pin site infection- Such an infection might be considered either a skin (SKIN) or soft tissue (ST) infection, depending on its depth, but not an SSI<br>Note: A laparoscopic trocar site for an operative procedure is not considered a stab wound.<br>• An infected burn wound is classified as BURN and is not an SSI.
Deep Incisional SSI	The date of event for infection occurs from the date of fracture to 90 days after the definitive fracture management surgery (where day 1 = the procedure date)<br>**AND**<br>involves deep soft tissues of the incision (e.g., fascial and muscle layers)<br>**AND**<br>patient has at least one of the following:<br>a. purulent drainage from the deep incision.<br>b. a deep incision that spontaneously dehisces, or is deliberately opened or aspirated by a surgeon, attending physician or other designee, and organism is identified by a culture or non-culture based microbiologic testing method which is performed for purposes of clinical diagnosis or treatment (e.g., not Active Surveillance Culture/Testing [ASC/AST]) or culture or non-culture based microbiologic testing method is not performed<br>**AND**<br>patient has at least one of the following signs or symptoms: fever (>38°C); localized pain or tenderness. A culture or non-culture based test that has a negative finding does not meet

Outcome	Description
	this criterion.<br>c. an abscess or other evidence of infection involving the deep incision that is detected on gross anatomical or histopathologic exam, or imaging test
Organ/Space SSI	Date of event for infection occurs from the date of fracture to 90 days after the definitive fracture management surgery (where day 1 = the procedure date)<br>**AND**<br>infection involves any part of the body deeper than the fascial/muscle layers, that is opened or manipulated during the operative procedure<br>**AND**<br>patient has at least one of the following:<br>a. purulent drainage from a drain that is placed into the organ/space (e.g., closed suction drainage system, open drain, T-tube drain, CT guided drainage)<br>b. organisms are identified from an aseptically-obtained fluid or tissue in the organ/space by a culture or non-culture based microbiologic testing method which is performed for purposes of clinical diagnosis or treatment (e.g., not Active Surveillance Culture/Testing [ASC/AST]).<br>c. an abscess or other evidence of infection involving the organ/space that is detected on gross anatomical or histopathologic exam, or imaging test evidence suggestive of infection.<br>**AND**<br>meets at least one criterion for a specific organ/space infection site listed in Table 3 of the CDC Procedure-associated Module (summarized in Table 4 below).[5] These criteria are found in the Surveillance Definitions for Specific Types of Infections chapter.[19]

*The CDC criteria has been modified to include all definitive fracture management surgeries, as opposed to including only National Healthcare Safety Network procedures that require infection reporting.

The CDC criteria for classifying SSIs will be followed. If multiple tissue levels are involved in the infection, the type of SSI (superficial incisional, deep incisional, or organ/space) reported will reflect the deepest tissue layer involved in the infection during the surveillance period. The date of event will be the date that the patient met criteria for the deepest level of infection: 1) Report infection that involves the organ/space as an organ/space SSI, whether or not it also involves the superficial or deep incision sites and 2) Report infection that involves the superficial and deep incisional sites as a deep incisional SSI. The most relevant National Healthcare Safety Network Organ/Space SSI classifications are summarized in **Table 4**.

Table 4: Relevant Organ/Space SSI Sites

Organ/Space SSI	BONE	Osteomyelitis
	JNT	Joint or bursa infection
	PJI	Prosthetic joint infection

All reported SSIs will be reviewed independently by an infection preventionist nurse and an orthopaedic surgeon who are members of the Adjudication Committee. Briefly, they will complete the review by examining all relevant information to determine if the SSI meets the CDC criteria of a superficial incisional SSI, deep incisional SSI, or organ/space SSI. The Committee will reach consensus on all reviewed SSIs. A hospital epidemiologist and infectious disease physician who are members of the Adjudication Committee will be available to provide guidance as needed. All members of the Adjudication Committee will be blinded to the treatment allocation.

4.8.2 Secondary Outcome

The secondary outcome is unplanned fracture-related reoperation within 12 months of the open fracture. This outcome has been used in previous open fracture trials and is defined as any unplanned surgery that occurred from the time of injury to 12 months post-injury that is associated with an infection at the operative site or contiguous to it, a wound-healing problem, or a fracture delayed union or nonunion. Common examples include any unplanned: 1) irrigation and debridement of surgical incisions or open fracture wounds due to infections or wound healing problems; 2) revision wound closure for dehiscence; 3) soft tissue coverage procedure for infected or necrotic wound; 4) fracture delayed union or nonunion surgery (such as bone grafting or implant exchange); and 5) reoperation for hardware or prosthesis failure due to infection or bone-healing problems. Removal of hardware for soft tissue prominence or periprosthetic fracture are common examples of reoperations that will not be considered outcome events. Two orthopaedic surgeons who are members of the Adjudication Committee will independently review all reported unplanned fracture-related reoperations to determine if they meet the criteria for being a study event. The Committee will reach consensus on all reviewed unplanned fracture-related reoperations.

4.8.3 Exploratory Outcomes

Two exploratory definitions of infection will be used for sensitivity analyses of the primary comparison. The first exploratory outcome is fracture-related infection (FRI) within 12 months of the open fracture, defined by the confirmatory criteria for FRI outlined in a 2018 consensus definition.[20] The FRI criteria has been selected as an exploratory outcome because the CDC criteria has been criticized for failing to adequately account for the complexities of infections in traumatic fractures.[20,21] The FRI criteria attempts to improve upon the ability to detect infections specifically in fracture patients; however, this definition of FRI has not been fully validated or widely adopted.

The confirmatory criteria include the presence of one or more of the following signs/symptoms:

1) Fistula, sinus or wound breakdown (with communication to the bone or the implant).
2) Purulent drainage from the wound or presence of pus during surgery.
3) Phenotypically indistinguishable pathogens identified by culture from at least two separate deep tissue/implant (including sonication-fluid) specimens taken during an operative intervention. In case of tissue, multiple specimens (3) should be taken, each with clean instruments (not superficial or sinus tract swabs). In cases of joint effusion, arising in a joint adjacent to a fractured bone, fluid samples obtained by sterile puncture may be included as a single sample.
4) Presence of microorganisms in deep tissue taken during an operative intervention, as confirmed by histopathological examination using specific staining techniques for bacteria or fungi.

The second exploratory outcome is SSI using the CDC criteria *within 12 months of the open fracture*. This secondary outcome will use the same diagnostic CDC reporting criteria for the primary outcome (**Tables 3 and 4**); however, the timeframe for this outcome will be expanded to include all SSIs that occur within 12 months of open fracture. Similar to the rationale for using the FRI outcome, and the recommendations for a minimum of 12 months follow-up for orthopaedic fracture outcomes[22], this expanded timeframe will detect infections that occur

beyond the standard CDC surveillance reporting periods. This modification of the CDC reporting periods has been used in previous orthopaedic fracture trials.[7,23]

An infection preventionist nurse and an orthopaedic surgeon member of the Adjudication Committee will review all reported SSIs to determine if they meet the FRI confirmatory criteria and / or the CDC criteria following the processes described above (see Section 4.8.1).

4.8.4 Data Collection and Participant Follow-up

After obtaining informed consent, study personnel will record the baseline data on the study CRFs. They will obtain this information directly from the participant or proxy, from the participant's medical chart, and the participant's treating orthopaedic surgeon and other health care providers. Data collection points include patient characteristics and injury details such as age, gender, comorbidities, mechanism of injury, socioeconomic status, and other injuries. Study personnel will also record the characteristics of up to three open fracture regions including the location, fracture severity, size of the wound, and degree of soft tissue injury using the Gustilo classification.[8,17,24]

Surgical data and in-hospital data will be collected throughout the participant's hospital stay. Detailed information will be collected regarding the surgical management of the open fracture(s), including the time to irrigation and debridement. For each open fracture region, study personnel will record the use of staged debridements, the presence or lack of skin closure between debridements, and the use of local antibiotics at the wound. The method(s) of initial and final fracture management will also be captured. Lastly, study personnel will record the use of negative pressure wound therapy for open wounds or in the presence of open wounds surgically closed. These treatment decisions are hypothesized to be associated markers of injury severity and potential confounders of the study interventions.

Study participants will be followed at 6 weeks, 3 months, 6 months, 9 months, and 12 months from their fracture. SSIs and unplanned fracture-related reoperations will be identified at the time of diagnosis/occurrence and/or during each participant's clinical assessment and medical record review that will occur during their routine outpatient clinic visits (**Table 2**). Detailed information on the SSI including the date of diagnosis, patient signs and symptoms, culture test results, method of treatment(s), and date of resolution will be collected. Study personnel will also record details about the participants' reoperations on the CRFs (e.g., date of reoperation, type of procedure, reason for procedure, etc.). In cases where the participant does not return to the clinic, study personnel will contact the participant by telephone, text, email, standard mail, and/or will review their medical record for any SSIs, and unplanned fracture-related reoperations. If the patient reports being treated at another hospital, study personnel will obtain the medical records from the other hospital. We have used this approach in our other multi-center trials (e.g., SPRINT, TRUST, FLOW, FAITH, HEALTH, etc.).[7,23,25–27]

To ensure research participant safety, serious adverse events (SAEs) will be documented at each follow-up visit and promptly submitted to the Methods Center and the local or central Institutional Review Board (IRB) or Research Ethics Board (REB) as per the required reporting processes.

Several strategies may be used to maximize follow-up including: 1) at the time of enrollment, each participant will provide their own telephone number, as well as the name and address of a primary care physician, and the names and phone numbers of three people at different addresses with whom the participant does not live with and who are likely to be aware of the participant's whereabouts; 2) participants will receive a reminder card upon discharge for their next follow up visit by the clinical site study personnel; 3) participants will receive text message reminders; 4) follow-up will coincide with normal surgical fracture clinic visits; and 5) if a participant refuses or is unable to return for the follow-up assessment, study personnel will determine his/her status with regard to major study outcomes by telephone, text, or email contact with the participant or the provided alternate contacts. Given these are standard of care visits and the participants will be receiving ongoing orthopaedic care for their acute fractures, minimal loss to follow-up is expected. Using these techniques, we expect greater than 95% follow-up at 3 months and 90% follow-up at 12 months post-fracture.

Participants will not be deemed lost to follow-up until the 12-month visit is overdue and all attempts to contact the participant have been exhausted. Participants will not be withdrawn from the study if the study protocol was not adhered to (e.g., allocated treatment not received, missed follow-up visits, etc.). The reasons for participants being withdrawn from the study will be documented (e.g., withdrawal of consent or lost to follow up).

5.0 STATISTICAL PLAN

5.1 Sample Size Determination

The overall objective of the trial is to determine the most effective aqueous pre-operative antiseptic skin solution for use during open fracture management. This will be achieved by comparing the effectiveness of 10% povidone-iodine versus 4% CHG surgical skin preparations. The primary outcome is the occurrence of SSI, as per the adapted CDC criteria (**Table 3**).[5] The secondary outcome is the occurrence of unplanned fracture-related reoperations within 12 months of injury. The sample size was calculated for the primary comparison between proportions of patients with SSI in each treatment group; however, it is expected that this estimate will also provide adequate power for the secondary outcome (unplanned fracture-related reoperation) because the baseline incidence (13%) and effect size for the reoperation outcome are expected to be similar to the SSI estimates.[7]

Assuming an ITT principle for the analysis, the sample size was calculated based on a cluster crossover design with the cluster as the unit of randomization and the patient as the unit of analysis. For complex study designs, such as a cluster-randomized crossover trial, simple formulas to calculate sample size or power may not capture the expected variability from the observed data.[28] Simulation methods were used to obtain empirical power calculations based on a feasible number of recruiting clusters and the expected number of open fracture patients.[28] The simulation estimates are designed to detect a difference between the treatment groups, accounting for between hospital variability inherent to a cluster-crossover trial design. We have assumed that the povidone-iodine solution will achieve a 0.65 risk ratio for SSI with a 12.5% SSI baseline incidence.[1,3,7] This represents approximately a 4% absolute risk reduction in SSI and reoperation. This effect was deemed more conservative than data reported by Swenson et al. and was consistent with feasible recruitment goals.[4]

Recent simulation data suggest that increasing the number of period crossovers can increase the statistical power of a given sample size.[29] To ensure the most conservative sample size estimate, we have based our sample size assumptions using a single crossover, 2 period design. The initial power estimate assumed 10 recruiting clusters, a 10% loss to follow-up rate,[7] and applying the between-cluster variance of 0.095 observed in the FLOW trial. Based on enrollment of a minimum of 1,540 open fracture patients, greater than 80% power would be achieved. Subsequent to the initial power calculations, the early trial experience demonstrated a need to increase the number of clusters to obtain a feasible recruitment pace. As a result, a minimum of 12 clusters will enroll participants into Aqueous-PREP. The increase in clusters results in a marginal increase in power (~2%).

Table 5 below outlines the summary of the initial sample size assumptions that yield ≥80% power to detect difference between the treatments. These sample size estimates are rounded up to the nearest multiple of 20 to ensure balance among the clinical sites and two interventions.

Table 5: Sample Size Assumptions

Baseline SSI Risk	10% Povidone-Iodine Risk Ratio	10% Povidone-Iodine Odds Ratio	Sample Size	Sample Size Increased by 10%
10.0%	0.62	0.59	1600	1760
10.0%	0.65	0.63	1960	2100
10.0%	0.67	0.65	2200	2420
10.0%	0.70	0.68	2600	2860
12.5%	0.62	0.59	1300	1440
12.5%	0.65	0.62	1400	1540
12.5%	0.67	0.64	1600	1760
12.5%	0.70	0.67	1800	1980
14.0%	0.62	0.58	1200	1320
14.0%	0.65	0.61	1300	1440
14.0%	0.67	0.64	1500	1660
14.0%	0.70	0.67	1800	1980

Note: *Between cluster ICC = 0.028; Between cluster variance* = 0.095; Between period variance = 0; Number of clusters = 10; Number of periods = 2; Alpha = 0.05

5.2 Statistical Methods

5.2.1 Analysis Plan Overview

A detailed statistical analysis plan will be published prior to the completion of the trial. The analysis and reporting of the results will follow the CONSORT guidelines for reporting of both pragmatic trials[30] and cluster-randomization trials.[31] The process of patient enrollment and flow throughout the study will be summarized using a flow-diagram. Patient demographics and baseline outcome variables will be summarized using descriptive summary measures expressed as mean (standard deviation) or median (interquartile range) for continuous variables depending on the distribution, and number (percent) for categorical variables.[32] An ITT principle will be adopted to analyze all outcomes and the unit of analysis will be the individual patients. Missing data will be assumed to be missing at random and will be handled with multiple imputation.[33,34]

The primary analysis will compare the treatment groups on the SSI outcome and the secondary analysis will compare the unplanned fracture-related reoperation outcome. The secondary

comparison will be conducted in accordance with best practice guidelines for secondary analyses. For all models, the results will be expressed as relative measure of effect (odds, risk, or hazard ratios) and corresponding two-sided 95% confidence intervals.

5.2.2 Analysis of the Study Outcomes

Adopting an ITT principle, multilevel regression models will be used. Correlation structures will be fit based on the observed between cluster and between period effects. A robust sandwich estimator will be used to analyze the primary and secondary outcomes.

For the primary outcome, SSI will be the dependent variable and the antiseptic solution (treatment group) will be the independent variable. For the secondary outcome, unplanned fracture-related reoperation will be the dependent variable and the antiseptic solution (treatment group) will be the independent variable. For both analyses, multiple imputation will be used to handle missing data.[34]

As the optimal methods for analyzing cluster crossover trials continue to evolve, the final statistical modeling technique to be used will be determined in accordance with contemporary best practices prior to the completion of participant follow-up. A separate Statistical Analysis Plan will be developed prior to study closeout. **Table 6** below shows a summary of the study outcomes, corresponding hypotheses, and currently proposed methods of analysis.

Table 6: Summary of Outcome Analysis Plan

Objective	Outcome		Hypothesis	Method of Analysis
	Name	Type		
To determine the effect of 10% povidone-iodine versus 4% CHG pre-operative antiseptic skin solutions on the incidence of SSI and unplanned fracture-related reoperation.	SSI	Binary	10% povidone-iodine will be more effective than CHG	Multi-level regression model
	Unplanned Fracture-Related Reoperation	Binary	10% povidone-iodine will be more effective than CHG	Multi-level regression model

Note: CHG = 4% chlorhexidine gluconate; SSI = Surgical Site Infection

5.2.3 Subgroup Analyses

Three subgroup analyses will be performed. The primary subgroup will be defined by the severity of open fracture (Gustilo-Anderson type I or II versus III).[8] Secondary subgroups will include: i) upper extremity versus lower extremity open fractures; and ii) none, minimal, or surface contamination versus contamination embedded in bone or deep soft tissues.[17] These analyses will be performed by comparing the effect estimates in both groups (interaction effect). We hypothesize that effect will differ by subgroup. These analyses will be approached and reported in accordance with best practices and guidelines for subgroup analyses.[35–38] **Table 7** below shows a summary of the subgroup analysis objectives, corresponding outcomes, hypotheses, and methods of analysis.

Table 7: Summary of Subgroup Analysis Plan

Objective	Outcome	Hypothesis	Method of

	Name	Type		Analysis
Primary				
Severity of open fracture (Gustilo-Anderson Type I or II vs. Type III)	SSI / Unplanned Fracture-Related Reoperation	Binary	10% povidone-iodine will be associated with a larger reduction in odds for SSI and reoperation than CHG in more severe fractures	Interaction of treatment by subgroup
Secondary				
Upper extremity vs. lower extremity fractures	SSI / Unplanned Fracture-Related Reoperation	Binary	10% povidone-iodine will be associated with a larger reduction in odds for SSI and reoperation than CHG in lower extremity compared to upper extremity fractures	Interaction of treatment by subgroup
None, minimal, or surface wound contamination vs. embedded wound contamination	SSI / Unplanned Fracture-Related Reoperation	Binary	10% povidone-iodine will be associated with a larger reduction in odds for SSI and reoperation than CHG in embedded contaminated wounds compared to wounds with no, minimal or surface contamination	Interaction of treatment by subgroup

Note: CHG = 4% chlorhexidine gluconate; SSI = Surgical Site Infection

5.2.4 Sensitivity Analyses

Assessment of the sensitivity or robustness of the findings to the key assumptions is essential in trials. The following sensitivity analyses may be conducted to explore the effects of alternative analysis models, alternative missing data approaches, balancing prognostic imbalance, as-treated analyses, variability in co-interventions, and alternative definitions of SSI.

1) *Using different analysis models:* There are several methods for analyzing cluster randomized crossover trials.[34,39] Therefore, our sensitivity analyses will explore alternative multi-level models with different correlation structures for the error.[35,39,38]

2) *Different methods of handling missing data:* There are several methods of handling missing data in trials.[39] Multiple imputation assumes that the data are missing at random—an assumption that is not verifiable in practice. Other imputation methods will be used such as worst case scenario to impute missing data and assess the robustness of the results.[40] For the worst case scenario analysis, we will assume that a random proportion of participants lost to follow-up experienced a study event. For this sensitivity analysis, the proportion assumed to experience a study event will be equivalent to the upper confidence interval of the observed pooled event rate for each study outcome.

3) *Adjusted analyses for prognostic imbalance:* We will also perform sensitivity analyses that assume prognostic imbalance between the two treatment groups based on the following key variables known to be risk factors for SSI or reoperation after open fracture: Gustilo fracture type, lower extremity fracture, wound contamination, time from injury to first debridement, antiseptic wound dressing in the emergency department, method of fixation, wound closure at initial debridement, age, work-related injury, and employment status.[16] Adjusted analyses including the above risk factors and treatment group as independent variables will be performed for the SSI and reoperation outcomes.

4) *As-treated analyses:* The proportion of surgical procedures receiving the incorrect, non-allocated antiseptic solution will be reported. "As-treated" sensitivity analyses will be performed using the solution received as the independent variable. For participants that were treated in a single open fracture surgery, they will be analyzed using the antiseptic solution received. For participants who received multiple open fracture surgeries, two analyses will be performed. First, the antiseptic solution used in their last surgery prior to a study outcome event will define their study treatment. For the second analysis, the antiseptic solution received in the majority of their fracture surgeries will define their study treatment. Participants who were treated with multiple open fracture management surgeries, but received equal exposure to both treatment solutions (e.g. one surgery with CHG and one surgery with iodine), will be analyzed within their originally allocated treatment group.

5) *Co-intervention variability:* Selective censoring of one or more clusters and / or treatment periods will be performed to further explore between-cluster and between-period variability identified in the primary and secondary outcome comparisons. These analyses will be used to explore the robustness of the study conclusions in the context of measured practice variations in co-interventions that differ between participating sites and / or evolve over the duration of the study recruitment. Results that are sensitive to the removal of a cluster(s) and / or period(s) will be reported, along with potential clinical hypotheses that are supported by the measured clinical practice variation.

6) *Exploratory SSI definitions:* The above analyses (See Section 5.2.2) will be repeated for the primary comparison using the FRI outcome and the CDC definition within 1 year of injury to determine if the study conclusions are sensitive to alternative definitions of SSI.

Table 8 below shows a summary of the objectives, corresponding outcomes, hypotheses, and methods for each potential sensitivity analysis.

Table 8: Summary of Sensitivity Analysis Plan

	Objective	Outcome		Hypothesis	Method of Analysis
		Name	Type		
1	Different analysis models	SSI / Reoperation	Binary	10% povidone-iodine will be more effective than CHG	Multi-level regression models with different correlation structures
2	Different missing data approach	SSI / Reoperation	Binary	10% povidone-iodine will be more effective than CHG	Multi-level regression models with missing data imputed using worst-case scenario
3	Baseline prognostic imbalance	SSI / Reoperation	Binary	10% povidone-iodine will be more effective than CHG	Multi-level regression models with prognostic variables & treatment group
4	As-treated analysis	SSI / Reoperation	Binary	10% povidone-iodine will be more effective than CHG	Multi-level regression models using "as treated" treatment group
5	Co-intervention variability	SSI / Reoperation	Binary	Cluster- and period-variability is related to co-interventions	Censoring of cluster(s) and/or period(s) with differences in co-interventions

	Objective	Outcome		Hypothesis	Method of Analysis
		Name	Type		
6	Exploratory SSI definitions	FRI / CDC SSI within 1 year	Binary	10% povidone-iodine will be more effective than CHG	Multi-level regression models

Note: CHG = 4% chlorhexidine gluconate; SSI = Surgical Site Infection; FRI = Fracture Related Infection; CDC = Centers for Disease Control and Prevention.

5.2.5 Interim Analysis

No formal interim analyses are planned and the trial will not be stopped early for benefit. The Data and Safety Monitoring Committee (see Section 7.5.5) will review frequent safety reports and will collectively make judgments on the strength of evidence and the absolute magnitude and seriousness of any safety signals.[41] The Data and Safety Monitoring Committee may make recommendations regarding the trial.

6.0 DATA MANAGEMENT

6.1 Case Report Forms and Data Transmission

Clinical sites will be provided with the trial CRFs prior to initiation of enrollment. Research personnel at each clinical site will submit the required data, as detailed on the CRFs, to the Methods Center using the REDCap Cloud electronic data capture system. Clinical site personnel will receive a unique login and password for the REDCap Cloud system and will be able to view and modify data for participants recruited at their clinical site.

6.2 Data Integrity

The REDCap Cloud system uses a variety of mechanisms for checking data at the time of entry including skip logic, range checks, and data type checks. Upon receipt of new data, the personnel at the Methods Center will query all missing, implausible, or inconsistent data. Clinical site personnel will be able to review of open queries in the system and will be required to respond promptly.

7.0 ETHICS AND DISSEMINATION

7.1 Research Ethics Approval

The McMaster University Methods Center and all participating clinical sites will receive REB or IRB approval prior to commencing participant enrollment. A central IRB and local IRBs/REBs will be used based on clinical site logistics. Prior to local commencement of the study, each clinical site will provide the Methods Center with a copy of their ethics approval.

7.2 Consent

In many cluster randomized comparative effectiveness trials, a waiver of consent is obtained from the IRB of Record. The rationale for the waiver of consent is that all patients will receive treatments that are effective and within standards of care, they will receive one of the study treatments as part of their routine care regardless of study participation, the data collection is minimal and obtained from the patient's medical records, the trial involves no more than minimal risk to the patient, and that the waiver of consent will not adversely affect the rights and welfare of the patient. Most of these concepts apply to the current trial, as the Aqueous-PREP

trial is comparative effectiveness research where patients will receive one of the preoperative antiseptic skin solutions regardless of their participation in the study. Additionally, patients are not included in the decision-making process for the choice of antiseptic preparation solution, and, in most situations, they are not even aware of which solution is used. However, in contrast to many cluster randomized crossover trials, Aqueous-PREP study personnel will need to contact participants directly to collect baseline and outcome data, as this information cannot be reliably obtained from the patients' medical records. Therefore, study personnel will obtain informed consent from patients prior to data collection. This consent process will allow study participants to be informed about the study rationale and provide consent for ongoing surveillance and data collection.

To increase enrollment and to avoid missing potential study participants, the consent process may take place up to 3 weeks post-fracture. Consultation during the study design phase with IRB members and patient advisors confirmed the acceptability of this flexible approach, where consent may be obtained after the intervention. The primary rationale for allowing consent after the intervention is consistent with the waiver of consent principles outlined above, but in addition, the patient and IRB stakeholders recognized that obtaining consent prior to the patient's first surgery could add undue decision making stress to a patient who is awaiting surgical management of a serious extremity injury; allowing consent after their surgery would likely facilitate an improved consent process.

The consent process will typically take place in the patient's hospital room or in the outpatient fracture clinic, either before or after the patient has had surgery(ies) to manage their open fracture. If the patient is unable to provide informed consent (e.g., due to their injury, language restrictions) within 3 weeks of their fracture, informed consent will be obtained from their proxy. In addition, if a patient has been discharged from hospital prior to being invited to participate in the study, a delegated member of the clinical care team may obtain their consent by telephone, as approved by the IRB of Record.

To obtain informed consent, delegated study personnel should follow the below procedures:

- Present study information in a manner that is understandable to the potential participant/proxy.
- Discuss the study with the potential participant/proxy and answer any questions he or she asks.
- Allow the potential participant/proxy an opportunity to discuss participation with their family, friends, or family physician, if desired.
- Confirm that the participant/proxy understands the risks and benefits of participating in the study and that their participation is voluntary.
- Complete and obtain signatures for informed consent form and obtain contact information from the participant/proxy.
- Provide /send the participant/proxy with a paper/electronic copy of the signed consent form.

Consent may be obtained electronically or using pen and paper consent forms, as approved by the IRB of Record.

The process of obtaining and documenting informed consent will be completed in accordance with local Good Clinical Practice recommendations. Consent procedures and forms, and the

communication, transmission and storage of patient data will comply with the IRB of Record and Department of Defense requirements for compliance with The Health Insurance Portability and Accountability Act.

Upon providing informed consent, study participants will be followed for 12 months from their fracture. Given the short follow-up time, the need for a regular reassessment of consent will not apply; however, participants may withdraw their consent at any time.

7.3 Confidentiality

Information about study participants will be kept confidential and will be managed in accordance with the below rules:

- All study-related information will be stored securely.
- All study participant information will be stored in locked file cabinets and accessible only to study personnel.
- All paper and electronic CRFs will be identified only by a coded participant number and initials.
- All databases will be password protected.

In the event that a participant revokes authorization to collect or use personal health information, the clinical site retains the ability to use all information collected prior to the revocation of participant authorization. For participants who have revoked authorization to collect or use personal health information, attempts should be made to obtain permission to collect at least vital status (i.e., primary outcome data) at the end of their scheduled study period.

7.4 Protocol Amendments

Any amendments to the study protocol which may affect the conduct of the study or the potential safety of or benefits to participants (e.g., changes to the study objectives, study design, sample size, or study procedures) will require a formal amendment to the protocol. Any protocol amendments will be approved by the Principal Investigators and will require approval by the McMaster University REB, the Central IRB, local IRBs/REBs, as well as the funders (as needed). Clinical sites will also be required to submit amendment requests to their IRB of Record to obtain approval for the amendment and to provide the Methods Center with a copy of this approval. Administrative changes (e.g., minor corrections or clarifications that have no effect on the way the study is conducted) will not need to undergo a formal amendment process.

7.5 Adverse Event Reporting and Definitions

7.5.1 Serious Adverse Event (SAE)

A SAE is any adverse event that is any of the following:

- Fatal
- Life threatening
- Requires or prolongs hospital stay
- Results in persistent or significant disability or incapacity
- A congenital anomaly or birth defect
- An important medical event

7.5.2 Unanticipated Problems Resulting in Risk to Participant or Others

Any incident, experience, or outcome that meets the following criteria:

- Unexpected in nature, severity, or frequency (e.g., not described in study-related documents such as the ethics-approved protocol or consent form, etc.).
- Related or possibly related to participation in the research (i.e., possibly related means there is reasonable possibility that the incident experience or outcome may have been caused by the procedures involved in the research).
- Suggests that the research places participants or others at greater risk of harm (including physical, psychological, economic, or social harm).

7.5.3 Clinical Site Reporting: Notifying the Methods Center

Clinical sites are responsible for reporting SAEs to the Methods Center via the REDCap Cloud system. Significant new information on ongoing SAEs should also be provided promptly to the Methods Center via the REDCap Cloud system. Unanticipated problems resulting in risk to participants or others are also to be reported promptly to the Methods Center.

7.5.4 Clinical Site Reporting – IRB and REB

Clinical sites are responsible for reporting SAEs and unanticipated problems resulting in risk to participants or others to their local REB/IRB or the Central IRB in accordance with local reporting requirements. Copies of each report and documentation of ethic board notification and receipt will be kept in the clinical site's study file.

7.5.5 Safety Monitoring

As per the FDA guidance document the *Establishment and Operation of Clinical Trial Data Monitoring Committees for Clinical Trial Sponsors*, a Data and Safety Monitoring Committee will oversee the safety of the trial participants and the overall conduct of the trial. The members of the Data and Safety Monitoring Committee will include two orthopaedic surgeons, an infectious disease expert, and a biostatistician. One orthopaedic surgeon will act as the Chair of the Committee. The Data and Safety Monitoring Committee will be responsible for safeguarding the interests of study participants, assessing the safety and efficacy of study procedures, and for monitoring the overall conduct of the study. The Data and Safety Monitoring Committee will frequently review enrollment and demographic summaries, listings of protocol deviations, and summaries and listings of SAEs. They will advise the Principal Investigators and study team on any concerns related to participant safety and trial conduct, and will make recommendations for the study to continue as designed, for study termination, for study continuation with major or minor modifications, or temporary suspension of enrollment until some uncertainty is resolved. We will develop a Data and Safety Monitoring Committee charter to guide the process.

7.6 Dissemination Policy

Results from the study will be submitted for publication regardless of whether there are significant findings. Every attempt will be made to ensure that the amount of time between completion of data collection and release of study findings are minimized.

8.0 DEPARTMENT OF DEFENSE REPORTING REQUIREMENTS

The following are the minimum reporting requirements with which the Principal Investigators must comply. The protocol will not be initiated at Department of Defense-funded clinical sites until written notification of approval of the research project is issued by the United States Army Medical Research and Materiel Command (USAMRMC) Office of Research Protections (ORP) Human Research Protection Office (HRPO).

- Substantive modifications to the research protocol and any modifications that could potentially increase risk to subjects must be submitted to the HRPO for approval prior to implementation. The USAMRMC ORP HRPO defines a substantive modification as a change in Principal Investigator, change or addition of an institution, elimination or alteration of the consent process, change to the study population that has regulatory implications (e.g. adding children, adding active duty population, etc), significant change in study design (i.e. would prompt additional scientific review) or a change that could potentially increase risks to subjects.
- Any changes of the IRB used to review and approve the research will be promptly reported to the USAMRMC ORP HRPO.
- All unanticipated problems involving risk to subjects or others must be promptly reported by telephone (301-619-2165), by email (usarmy.detrick.medcom-usamrmc.other.hrpo@mail.mil), or by facsimile (301-619-7803) to the HRPO. A complete written report will follow the initial notification. In addition to the methods above, the complete report can be sent to the US Army Medical Research and Materiel Command, ATTN: MCMR-RP, 810 Schreider Street, Fort Detrick, Maryland 21702-5000.
- Suspensions, clinical holds (voluntary or involuntary), or terminations of this research by the IRB, the institution, the Sponsor, or regulatory agencies will be promptly reported to the USAMRMC ORP HRPO.
- A copy of the continuing review approval notification by the IRB of Record must be submitted to the HRPO as soon as possible after receipt. Please note that the HRPO also conducts random audits at the time of continuing review. Additional information and documentation may be requested at that time.
- The final study report, including any acknowledgement documentation and supporting documents, must be submitted to the HRPO when available.
- The knowledge of any pending compliance inspection/visit by the FDA, Department of Health and Human Services Office of Human Research Protections, or other government agency concerning this research, the issuance of Inspection Reports, FDA Form 483, warning letters or actions taken by any regulatory agencies including legal or medical actions and any instances of serious or continuing noncompliance with the regulations or requirements, must be promptly reported to the HRPO.

9.0 SUB-STUDY: PATIENT EXPERIENCES IN THE AQUEOUS-PREP AND PREPARE TRIALS

9.1 Introduction

Patient and stakeholder involvement in the design of randomized controlled trials is increasingly becoming recognized as an essential component of a trial’s success.[42,43] Patient and stakeholder involvement (PSI) has been seen as the paradigm shift from research being done “to” or “for”

patients, to research being performed "with" or "by" patients themselves.[44] PSI allows for democratization of the research process and empowering patients throughout the entire research process – from design through to knowledge dissemination.[45] Research has found that patients and stakeholders are motivated to be involved in research for a wide variety of reasons, including a desire to contribute to research for the benefit of others.[46]

Prior research has argued that PSI enhances the focus of clinical trials on outcomes that are relevant to patients themselves, thus increasing the utility of any research findings.[47] Furthermore, PSI has been argued to improve recruitment and retention rates, while raising the quality of research findings and ultimately helping with the dissemination of research findings.[48] Lastly, PSI may be able to improve patient safety when patients are involved in safety reporting in hospital settings.[49]

Despite these findings, a recent systematic review estimates that far less than 1% of clinical trials engage patients in any meaningful or active way.[50] From the onset of the PREP-IT trials (i.e. the Aqueous-PREP and PREPARE trials), the PREP-IT investigators have engaged multiple patient-partners and stakeholders in the design, conduct, and implementation of the PREP-IT trials. One of our engagement goals is to identify ways in which we can better engage with PREP-IT study participants. To support this goal, we seek to learn about PREP-IT participants' experiences within the PREP-IT trials. This knowledge will be used to improve the study team's ability to engage study participants and provide study information in a meaningful and accessible manner. Additionally, the unique design of the PREP-IT trials (e.g., consent after the intervention, minimal follow-up, minimal requirements for participants) provides a novel trial to investigate this question. This led to the current sub-study.

9.2 Rationale and Objectives

One of the mandates of the PREP-IT program is to improve orthopaedic fracture research through meaningful engagement with our patient-partners and stakeholders. The objective of this sub-study is to learn about PREP-IT participants' experiences with participating in the Aqueous-PREP or PREPARE trial. The results of this sub-study will be used to develop strategies to better engage research participants both in the PREP-IT trials as well as in future clinical trials.

9.3 Sub-Study Design

This sub-study will consist of an exit survey that will be given to a subset of participants in the PREP-IT trials. Select clinical sites participating in the Aqueous-PREP and / or PREPARE trial will be invited to participate in the sub-study.

The exit survey is comprised of 14 questions that includes multiple choice and brief open-ended questions. All of the questions use clear and simple language written at or below a grade eight reading level to enhance the validity of results. The survey length has been kept to a minimum to maximize response rate and limit barriers that would affect its proper completion.

The survey was created after reviewing the current literature and with input from the PREP-IT investigators, research coordinators, patient-partners, and stakeholders. Engaging the larger study team follows the PREP-IT philosophy of meaningful engagement, as well as helps to

ensure that no vital questions were missed and that the survey wording is clear and easily understandable to the target audience. The questionnaire was pre-tested on a sample of convenience.

9.4 Survey Participants and Distribution

All potential substudy participants, or their proxies, will be required to provide informed consent specifically for the substudy prior to completing the survey. Informed consent for the substudy may be obtained at the time of enrollment in the Aqueous-PREP or PREPARE trial using consent procedures described in sections 4.3.1 and 7.2, or in-person at a subsequent follow-up visit or time of survey administration using a pen and paper consent form. The patient or proxy must be provided with a copy of the signed informed consent form. All sites within the United States of America must conduct their consenting process in accordance with HIPAA (Health Insurance Portability and Accountability Act) regulations as approved by their institutions, and sites in Canada must comply with the Personal Information Protection and Electronic Documents Act (PIPEDA).

Clinical sites participating in the sub-study will offer the survey to all eligible participants at the time they complete their one-year follow up visit. The survey will be sent to participants either through mail, email or RedCap Cloud, given to them on paper at a follow-up visit, or administered over the phone, depending on each individual participant's preference. The Research Coordinator may also telephone or text the participant to remind them to complete the exit survey. We will document the number of participants invited to participate in the survey as well as the number of participants who decline participation.

9.5 Data Entry

The exit survey responses will be entered into the Aqueous-PREP / PREPARE trial's electronic data capture (EDC) system.

9.6 Sample Size

Sample size was calculated using a 5% margin of error, with 95% confidence intervals, a potential population of all patients who have completed one year follow up (approximately 1600 patients) and an expected response rate of 50%. With this in mind, a sample size of approximately 310 patients who complete every survey question will be required.[51] As such, the survey will be distributed to all participants at participating clinical sites until our sample size of at least 310 participants is achieved.

9.7 Data Analysis

We will summarize all variables with frequencies and percentages. The short form questions will be coded appropriately based on themes.

9.8 Anticipated Implications of Results

This research serves as an important step towards understanding patients' perspectives as participants in a clinical trial. Additionally, the research may influence how future clinical trials are designed and conducted, with the overall goal of a greater focus on the patient experience and increasing patient involvement in research. Lastly, the results of this sub-study could help

the study team to develop aids (e.g., posters, pamphlets, etc.) to improve patients’ understanding of clinical research and overall experience with the PREP-IT trials.

10.0 REFERENCES

1. Darouiche RO, Wall Jr. MJ, Itani KMF, et al. Chlorhexidine-Alcohol versus Povidone-Iodine for Surgical-Site Antisepsis. *N Engl J Med*. 2010;362(1):18-26. doi:10.1056/NEJMoa0810988
2. Tuuli MG, Liu J, Stout MJ, et al. A Randomized Trial Comparing Skin Antiseptic Agents at Cesarean Delivery. *N Engl J Med*. 2016;374(7):647-655. doi:10.1056/NEJMoa1511048
3. Swenson BR, Sawyer RG. Importance of Alcohol in Skin Preparation Protocols. *Infect Control Hosp Epidemiol*. 2010;31(09):977. doi:10.1086/655843
4. Swenson BR, Hedrick TL, Metzger R, Bonatti H, Pruett TL, Sawyer RG. Effects of preoperative skin preparation on postoperative wound infection rates: a prospective study of 3 skin preparation protocols. *Infect Control Hosp Epidemiol*. 2009;30(10):964-971. doi:10.1086/605926
5. Centers for Disease Control and Prevention (CDC). *Surgical Site Infection (SSI) Event*.; 2017.
6. Schoenfeld AJ, Dunn JC, Belmont PJ. Pelvic, spinal and extremity wounds among combat-specific personnel serving in Iraq and Afghanistan (2003-2011): A new paradigm in military musculoskeletal medicine. *Injury*. 2013;44(12):1866-1870. doi:10.1016/j.injury.2013.08.001
7. FLOW Investigators. A Trial of Wound Irrigation in the Initial Management of Open Fracture Wounds. *N Engl J Med*. 2015;373(27):2629-2641. doi:10.1056/NEJMoa1508502
8. Gustilo R, Merkow R, Templeman D. Management of open fractures. *J Bone Jt Surg*. 1990;72(2):299-304.
9. Mcdonnell G, Russell AD. Antiseptics and disinfectants: Activity, action, and resistance. *Clin Microbiol Rev*. 1999;12(1):147-179. doi:10.1007/s13398-014-0173-7.2
10. Dumville J, McFarlane E, Edwards P, Lipp A, Holmes A, Liu Z. Preoperative skin antiseptic for prevention of surgical wound infections after clean surgery. *Cochrane Database Syst Rev*. 2013;(3):CD003949. doi:10.1002/14651858.CD003949.pub4
11. Kunisada T, Yamada K, Oda S, Hara O. Investigation on the efficacy of povidone-iodine against antiseptic-resistant species. *Dermatology*. 1997;195 Suppl(suppl 2):14-18. http://www.ncbi.nlm.nih.gov/pubmed/9403250.
12. Russell AD, Day MJ. Antibacterial activity of chlorhexidine. *J Hosp Infect*. 1993;25:229-238.
13. Tsang STJ, Mills LA, Frantzias J, Baren JP, Keating JF, Simpson AHRW. Exchange nailing for nonunion of diaphyseal fractures of the tibia: our results and an analysis of the risk factors for failure. *Bone Joint J*. 2016;98-B(4):534-541. doi:10.1302/0301-620X.98B4.34870
14. Mills L, Tsang J, Hooper G, Keenan G, Simpson A. The multifactorial aetiology of fracture nonunion and the importance of searching for latent infection. *Bone Joint Res*. 2016;5(10):512-519. doi:10.1302/2046-3758.510.BJR-2016-0138
15. Torbert JT, Joshi M, Moraff A, et al. Current Bacterial Speciation and Antibiotic Resistance in Deep Infections After Operative Fixation of Fractures. *J Orthop Trauma*. 2015;29(1):7-17. doi:10.1097/BOT.0000000000000158
16. Gitajn IL, Sprague S, Petrisor B, Jeray KJ, O'Hara N, Nascone J, Bhandari M, Slobogean G, FLOW Investigators. Predictors of Complications in Severe Open Fractures. *J Orthop Trauma*.

17. Müller M, Nazarian S, Koch P, Schatzker J. The Comprehensive Classification of Fractures of Long Bones. *Springer-Verlag Berlin Heidelb*. 1990. http://www.springer.com/kr/book/9783540181651.
18. Järvinen TLN, Sihvonen R, Bhandari M, et al. Blinded interpretation of study results can feasibly and effectively diminish interpretation bias. *J Clin Epidemiol*. 2014;67(7):769-772. doi:10.1016/j.jclinepi.2013.11.011
19. Centers for Disease Control and Prevention (CDC). *CDC/NHSN Surveillance Definitions for Specific Types of Infections*.; 2017.
20. Metsemakers WJ, Morgenstern M, McNally MA, et al. Fracture-related infection: A consensus on definition from an international expert group. *Injury*. 2018. doi:10.1016/j.injury.2017.08.040
21. Metsemakers WJ, Kuehl R, Moriarty TF, et al. Infection after fracture fixation: Current surgical and microbiological concepts. *Injury*. 2018. doi:10.1016/j.injury.2016.09.019
22. Journal of Orthopaedic Trauma. Journal of Orthopaedic Trauma: Online submission and review system. http://edmgr.ovid.com/jot/accounts/ifauth.htm. Published 2019. Accessed April 10, 2019.
23. Study to Prospectively Evaluate Reamed Intramedullary Nails in Patients with Tibial Fractures Investigators, Bhandari M, Guyatt G, et al. Randomized trial of reamed and unreamed intramedullary nailing of tibial shaft fractures. *J Bone Joint Surg Am*. 2008;90(12):2567-2578. doi:10.2106/JBJS.G.01694
24. Gustilo R, Anderson J. Prevention of infection in the treatment of one thousand and twenty-five open fractures of long bones: retrospective and prospective analyses. *J Bone Joint Surg Am*. 1976;58(4):453-458.
25. TRUST Investigators writing group, Busse JW, Bhandari M, et al. Re-evaluation of low intensity pulsed ultrasound in treatment of tibial fractures (TRUST): randomized clinical trial. *BMJ*. 2016;355:i5351. doi:10.1136/bmj.i5351
26. FAITH Investigators. Fracture fixation in the operative management of hip fractures (FAITH): an international, multicentre, randomised controlled trial. *Lancet*. 2017;389(10078):1519-1527. doi:10.1016/S0140-6736(17)30066-1
27. Bhandari M, Devereaux PJ, Einhorn TA, et al. Hip fracture evaluation with alternatives of total hip arthroplasty versus hemiarthroplasty (HEALTH): protocol for a multicentre randomised trial. *BMJ Open*. 2015;5(2):e006263. doi:10.1136/bmjopen-2014-006263
28. Reich NG, Myers JA, Obeng D, Milstone AM, Perl TM. Empirical power and sample size calculations for cluster-randomized and cluster-randomized crossover studies. *PLoS One*. 2012;7(4):e35564. doi:10.1371/journal.pone.0035564
29. Grantham KL, Kasza J, Heritier S, Hemming K, Litton E, Forbes AB. How many times should a cluster randomized crossover trial cross over? *Stat Med*. 2019. doi:10.1002/sim.8349
30. Zwarenstein M, Treweek S, Gagnier JJ, et al. Improving the reporting of pragmatic trials: an extension of the CONSORT statement. *BMJ*. 2008;337:a2390. doi:10.1136/BMJ.A2390
31. Campbell MK, Piaggio G, Elbourne DR, Altman DG. Consort 2010 statement: extension to cluster randomised trials. *BMJ*. 2012;345(sep04 1):e5661-e5661. doi:10.1136/bmj.e5661
32. Thabane L, Akhtar-Danesh N. Guidelines for reporting descriptive statistics in health research. *Nurse Res*. 2008;15(2):72-81. doi:10.7748/nr2008.01.15.2.72.c6331

33. Sterne J, White I, Carlin J, et al. Multiple imputation for missing data in epidemiological and clinical research: potential and pitfalls. *Br Med J*. 2009;338(July):b2393. doi:10.1136/bmj.b2393
34. Little RJA, Rubin DB. *Statistical Analysis with Missing Data*.; 2002. doi:10.2307/1533221
35. Wang R, Lagakos SW, Ware JH, Hunter DJ, Drazen JM. Statistics in Medicine — Reporting of Subgroup Analyses in Clinical Trials. *N Engl J Med*. 2007;357(21):2189-2194. doi:10.1056/NEJMsr077003
36. Sun X, Ioannidis JPA, Agoritsas T, Alba AC, Guyatt G. How to use a subgroup analysis: users' guide to the medical literature. *JAMA*. 2014;311(4):405-411. doi:10.1001/jama.2013.285063
37. Sun X, Briel M, Walter SD, Guyatt GH. Is a subgroup effect believable? Updating criteria to evaluate the credibility of subgroup analyses. *BMJ*. 2010;340(9209):c117. doi:10.1136/bmj.c117
38. Sun X, Briel M, Busse JW, et al. Subgroup Analysis of Trials Is Rarely Easy (SATIRE): a study protocol for a systematic review to characterize the analysis, reporting, and claim of subgroup effects in randomized trials. *Trials*. 2009;10:101. doi:10.1186/1745-6215-10-101
39. Morgan KE, Forbes AB, Keogh RH, Jairath V, Kahan BC. Choosing appropriate analysis methods for cluster randomised cross-over trials with a binary outcome. *Stat Med*. 2017;36(2):318-333. doi:10.1002/sim.7137
40. Zhang Y, Alyass A, Vanniyasingam T, et al. A systematic survey of the methods literature on the reporting quality and optimal methods of handling participants with missing outcome data for continuous outcomes in randomized controlled trials. *J Clin Epidemiol*. 2017;88:67-80. doi:10.1016/j.jclinepi.2017.05.016
41. Pocock SJ, Clayton TC, Stone GW. Challenging Issues in Clinical Trial Design Part 4 of a 4-Part Series on Statistics for Clinical Trials. *J Am Coll Cardiol*. 2015;66(25):2886-2898. doi:10.1016/j.jacc.2015.10.051
42. Chalmers I. What do I want from health research and researchers when I am a patient? *BMJ*. 1995;310(6990):1315-1318. doi:10.1136/bmj.310.6990.1315
43. Domecq JP, Prutsky G, Elraiyah T, et al. Patient engagement in research: A systematic review. *BMC Health Serv Res*. 2014;14:89. doi:10.1186/1472-6963-14-89
44. INVOLVE. *Briefing Notes for Researchers: Public Involvement in NHS, Public Health and Social Care Research*.; 2012. https://www.invo.org.uk/wp-content/uploads/2014/11/9938_INVOLVE_Briefing_Notes_WEB.pdf.
45. Esmail L, Moore E, Rein A. Evaluating patient and stakeholder engagement in research: Moving from theory to practice. *J Comp Eff Res*. 2015;4(2):133-145. doi:10.2217/cer.14.79
46. Tarpey M, INVOLVE Support Unit. *Why People Get Involved in Health and Social Care Research: A Working Paper*. Vol July.; 2006. http://www.invo.org.uk/wp-content/uploads/documents/whypeoplegetinvolvedinresearch2006.pdf.
47. Brett J, Staniszewska S, Mockford C, et al. A Systematic Review of the Impact of Patient and Public Involvement on Service Users, Researchers and Communities. *Patient*. 2014;7(4):387-395. doi:10.1007/s40271-014-0065-0
48. Robinson A. Patient and public involvement: In theory and in practice. *J Laryngol Otol*. 2014;128(4):318-325. doi:10.1017/S0022215114000735

49. Lawton R, O'Hara JK, Sheard L, et al. Can patient involvement improve patient safety? A cluster randomised control trial of the Patient Reporting and Action for a Safe Environment (PRASE) intervention. *BMJ Qual Saf*. 2017;26(8):622-631. doi:10.1136/bmjqs-2016-005570
50. Fergusson D, Monfaredi Z, Pussegoda K, et al. The prevalence of patient engagement in published trials: A systematic review. *Res Involv Engagem*. 2018;4:17. doi:10.1186/s40900-018-0099-x
51. Bartlett JE, Kotrlik JWKJW, Higgins C. Organizational research: Determining appropriate sample size in survey research appropriate sample size in survey research. *Inf Technol Learn Perform J*. 2001;19(1):43-50.
