# Supplementary material for: Effectiveness of Iodophor vs Chlorhexidine Solutions for Surgical Site Infections and Unplanned Reoperations for Patients Who Underwent Fracture Repair: The PREP-IT Master Protocol
Source: JAMA Netw Open. 2020 Apr 7;3(4):e202215. doi: 10.1001/jamanetworkopen.2020.2215 (PMC7139274; doi:10.1001/jamanetworkopen.2020.2215)
Supplement: Supplement 2. — PREPARE Trial Protocol [file jamanetwopen-3-e202215-s002.pdf]

PREP-IT
A Program of Randomized trials to Evaluate Pre-operative antiseptic skin solutions In orthopaedic Trauma

PREPARE: A Pragmatic Randomized trial Evaluating Pre-operative Alcohol skin solutions in FRactured Extremities

PREPARE PROTOCOL

Version: 2.1

Date: 04-Nov-2019

This study is funded by the Patient-Centered Outcomes Research Institute (PCORI) (PCS-1609-36512).

The PREPARE trial is part of the PREP-IT research program. The protocol is the confidential intellectual property of the Principal Investigators and PREP-IT Steering Committee, and the protocol cannot be used in any form without the expressed written permission of the Principal Investigators.

Reviewed and Approved by:		
Dr. Gerard Slobogean (Principal Investigator)	***Signature:***	***Date:*** 04-Nov-2019
Dr. Sheila Sprague (Principal Investigator)	***Signature:***	***Date:*** 04-Nov-2019
Dr. Mohit Bhandari (Principal Investigator)	***Signature:***	***Date:*** 04-Nov-2019

TABLE OF CONTENTS

LIST OF ABBREVIATIONS **4**
STUDY SUMMARY **5**
1.0 INTRODUCTION **8**
1.1 Extremity Fractures and Surgical Site Infections 8
1.2 Prevention of Infection 8
1.3 Rationale for Pre-Operative Antiseptic Skin Solution Prophylaxis 8
1.4 Extrapolating Evidence from Other Surgical Disciplines to Fracture Surgery is Problematic 10
1.5 Why Iodophor Skin Preparations May Reduce Operative Extremity Fracture SSI 11
1.6 Why Iodophor Skin Preparations May Reduce Extremity Fracture Reoperations . 11
1.7 Lack of Surgeon Consensus 12
2.0 STUDY OBJECTIVES AND HYPOTHESES **12**
2.1 Study Objectives and Hypotheses 12
2.2 Open and Closed Fracture Study Populations 13
2.3 Subgroup Objectives 13
2.3.1 Open Fracture Subgroups 13
2.3.2 Closed Fracture Subgroups 13
2.3.3 Subgroup Hypotheses 13
3.0 TRIAL DESIGN **14**
3.1 Summary 14
3.2 Pragmatic-Explanatory Continuum 15
4.0 METHODS **16**
4.1 Study Setting, Cluster Eligibility, and Selection of Clusters 16
4.2 Eligibility Criteria 17
4.2.1 Open Fracture Population Eligibility Criteria 17
4.2.2 Closed Fracture Population Eligibility Criteria 18
4.2.3 Additional Eligibility Considerations 18
4.3 Recruitment Strategy 19
4.3.1 Patient Screening & Consent 19
4.4 Managing Patient Volume 20
4.4.1 Enrollment of Patients from Only One Fracture Cohort 20
4.4.2 Enrollment Sampling Plan 21
4.4 Randomization Methods 21
4.5 Blinding 21
4.6 Description of the Interventions 22
4.6.1 Initial Run-In Phase 22
4.6.2 First Intervention Phase 22
4.6.3 Second Intervention Phase 23
4.6.4 Special Considerations for Ongoing Treatment Crossovers 23
4.6.5 Evaluation of Site Performance and Removal of Clinical Sites 23
4.6.6 Application of Pre-Operative Antiseptic Skin Solutions 23
4.6.7 Patients with Multiple Planned Surgeries 24
4.6.8 Iodophor Antiseptic Solution 24

4.6.9 CHG Antiseptic Solution 24
4.7 Perioperative Co-Interventions 25
4.8 Outcome Measures 25
4.8.1 Primary Outcome 25
4.8.2 Secondary Outcome 27
4.8.4 Data Collection and Participant Follow-up 28
5.0 STATISTICAL PLAN 30
5.1 Sample Size Determination 30
5.2 Statistical Methods 31
5.2.1 Analysis Plan Overview 31
5.2.2 Analysis of the Study Outcomes 32
5.2.3 Subgroup Analyses 32
5.2.4 Sensitivity Analyses 33
5.2.5 Interim Analysis 36
6.0 DATA MANAGEMENT 36
6.1 Case Report Forms and Data Transmission 36
6.2 Data Integrity 36
7.0 ETHICS AND DISSEMINATION 36
7.1 Research Ethics Approval 36
7.2 Consent 36
7.3 Confidentiality 38
7.4 Protocol Amendments 38
7.5 Adverse Event Reporting and Definitions 39
7.5.1 Serious Adverse Event (SAE) 39
7.5.2 Unanticipated Problems Resulting in Risk to Participant or Others 39
7.5.3 Serious Unexpected Adverse Drug Reactions 39
7.5.4 Adverse Event Reporting 39
7.5.5 Clinical Site Reporting – IRB and REB 40
7.5.6 Data and Safety Monitoring Committee 40
7.6 Dissemination Policy 40
8.0 SUB-STUDY: PATIENT EXPERIENCES IN THE AQUEOUS-PREP AND PREPARE TRIALS 40
8.1 Introduction 40
8.2 Rationale and Objectives 41
8.3 Sub-Study Design 41
8.4 Survey Participants and Distribution 42
8.5 Data Entry 42
8.6 Sample Size 42
8.7 Data Analysis 42
8.8 Anticipated Implications of Results 42
9.0 REFERENCES 43

LIST OF ABBREVIATIONS

Abbreviation	Explanation
CDC	Centers for Disease Control and Prevention
CEO	Center for Evidence-Based Orthopaedics
CHG	Chlorhexidine gluconate
CI	Confidence interval
CRF	Case report form
EDC	Electronic data capture
FDA	Food and Drug Administration
FLOW	Fluid Lavage of Open Wounds trial
FRI	Fracture-Related Infection
ITT	Intention-to-treat
IRB	Institutional Review Board
PREPARE	A Pragmatic Randomized trial Evaluating Pre-operative Alcohol skin solutions in FRactured Extremities
REB	Research Ethics Board
SAE	Serious adverse event
SSI	Surgical site infection

STUDY SUMMARY

**Methodology**	Cluster randomized crossover design.
**Coordinating Center**	This study will be centrally coordinated by the Methods Center at the Center for Evidence-Based Orthopaedics (CEO), McMaster University, Hamilton, Ontario and by the Administrative Center within the Department of Orthopaedics at the University of Maryland, R Adams Cowley Shock Trauma Center, Baltimore, Maryland.
**Clinical Sites**	At least 18 clinical sites in North America. Additional clinical sites will be included or removed as needed.
**Background**	The prevention of infection is an important goal influencing peri-operative care of extremity fracture patients. Standard practice in the operative management of extremity fractures includes sterile technique and pre-operative skin preparation with an antiseptic solution. The available solutions kill bacteria and decrease the quantity of native skin flora, thereby decreasing surgical site infection (SSI). While there is extensive guidance on specific procedures for prophylactic antibiotic use and standards for sterile technique, the evidence regarding the choice of antiseptic skin preparation solution is very limited for extremity fracture surgery.
**Objectives**	The overarching objective of this trial is to compare the effectiveness of iodine povacrylex (0.7% free iodine) in 74% isopropyl alcohol versus 2% chlorhexidine gluconate (CHG) in 70% isopropyl alcohol for the management of extremity fractures that require surgical treatment. The primary outcome for comparison is *surgical site infection (SSI),* and the secondary outcome is *unplanned fracture-related reoperation.*
**Open and Closed Fractures Populations**	Open fracture patients and closed fracture patients represent two distinct populations within extremity fracture surgery. Open and closed fracture participants will be recruited separately to independently compare the effectiveness of the study solutions in each population. Therefore, our effectiveness comparisons will be performed separately within the open fracture and closed fracture populations.
**Subgroup Objectives**	The PREPARE trial will also explore the possibility of differential treatment effects of the pre-operative antiseptic skin solutions among clinically important subgroups. The open fracture subgroups will be defined by i) the severity of open fracture (Gustilo-Anderson type I or II versus III);[1] ii) upper extremity versus lower extremity open fractures; iii) severity of wound contamination; and, iv) presence or absence of comorbidities that affect wound healing. The closed fracture subgroups will be defined by: i) severity of soft

	tissue injury (higher Tscherne injuries) and ii) presence or absence of comorbidities that affect wound healing.
**Diagnosis and Main Inclusion Criteria**	All patients 18 years of age or older who present to a recruiting hospital for treatment of an open fracture(s) of the appendicular skeleton will be screened for participation within 3 weeks of their fracture. All patients 18 years of age or older who present to a recruiting hospital for surgical treatment of a closed lower extremity or pelvic fracture(s) will be screened for participation within 6 weeks of their fracture. Eligible patients must have an open fracture of the appendicular skeleton or have a closed lower extremity or pelvic fracture, and their fractures must be definitively managed with a surgical implant (e.g., internal fixation, external fixation (open fractures and in closed fractures that require a surgical incision), joint prosthesis, etc.).
**Treatment Groups**	The PREPARE trial will compare the most common alcohol-based pre-operative antiseptic skin solutions used during extremity fracture surgery. The iodine-based treatment intervention is an antiseptic solution comprised of iodine povacrylex (0.7% free iodine) in 74% isopropyl alcohol. 3M™ DuraPrep™ [3M Health Care, St Paul, MN], will be the commercial product used. The CHG intervention is an antiseptic solution comprised of 2% CHG in 70% isopropyl alcohol. ChloraPrep® [CareFusion Inc., Leawood, KS, USA] will be the commercial product used.
**Randomization**	Treatment allocation will be determined using a cluster-randomized crossover trial design. The open and closed fracture populations will be treated with the same allocated solution at all times during the trial. The order of treatment allocation for each orthopaedic practice will be randomly assigned using a computer-generated randomization table. Each site will start with the initially allocated study solution and eventually crossover to the other solution for their second recruitment period. This process of alternating treatments will repeat approximately every 2 months as dictated by the initial randomization.
**Study Outcomes**	The primary outcome is SSI, guided by the Centers for Disease Control and Prevention's (CDC) National Healthcare Safety Network reporting criteria, which includes superficial incisional SSI within 30 days and deep incisional or organ/space SSI within 90 days of definitive fracture management surgery. The secondary outcome is the occurrence of an unplanned fracture-related reoperation within 12 months of the fracture. Alternative definitions of SSI, including the confirmatory criteria for Fracture-Related Infection (FRI) and the CDC criteria within 1 year of injury will be used for sensitivity analyses of the primary comparison. All study outcomes will be adjudicated by a blinded committee using clinical notes and radiographs.

**Follow-Up**	Study participants will be followed at 6 weeks, 3 months, 6 months, 9 months, and 12 months from their fracture.
**Sample Size**	A minimum of 1,540 participants with open fractures and a minimum of 6,280 participants with closed lower extremity or pelvic fractures will be included in PREPARE.
**Significance**	SSIs are often devastating complications for fracture patients because of the resultant reoperations, adverse events from antibiotic courses, and fracture healing difficulties. Given the substantial impact of extremity fractures, maximizing the effectiveness of current prophylactic procedures is essential. The PREPARE trial will provide necessary evidence to guide the prevention of SSIs in fracture care, and the trial is poised to have a significant impact on the care and outcomes of extremity fracture patients.

1.0 INTRODUCTION

1.1 Extremity Fractures and Surgical Site Infections

More than one million Americans suffer an extremity fracture (broken bone in the arm, leg, or pelvis) that requires surgery each year.[2,3] Approximately 5% (or 50,000) of surgical fracture patients develop a surgical site infection (SSI),[4,5] which is twice the rate among most surgical patients and nearly five times the rate among patients undergoing elective orthopaedic surgeries (e.g., joint replacement).[6] Patients who develop a SSI after their fracture fixation surgery experience a long and difficult treatment pathway. Researchers have identified that when a fracture patient experiences a SSI, they typically undergo at least two additional surgeries to control the infection, spend a median of 14 additional days in the hospital, and have significantly lower health related quality of life (HRQL).[7] Similarly, results from the recently completed Fluid Lavage of Open Wounds (FLOW) trial confirmed that patients who had a SSI, or another complication, that required an additional surgery reported significantly lower physical and mental HRQL in the 12 months following their fracture compared to patients who did not experience a SSI.[8] In the most severe cases, when a SSI cannot be controlled, a limb amputation becomes necessary.

Open fractures, closed lower extremity fractures, and pelvic fractures represent some of the most severe musculoskeletal injuries.[9] Due to their high-energy mechanisms, these fractures are often accompanied by soft-tissue injuries that contribute to unacceptably poor outcomes. The FLOW trial of 2,447 open fracture patients reported a 13.2% incidence of open fracture-related reoperations;[4] Closed fractures of the lower extremity are also at high risk of complications, particularly when compared to closed upper extremity fractures. For example, the rate of SSI in closed tibial plateau and plafond fractures range from 5.6 – 11.9%,[10–14] although some cohort studies have reported infection rates as high as 25.0%.[15] This is contrast with SSI rates of <5% for common upper extremity fractures like humeral shaft, forearm, or distal radius fractures.[16,17] This is further illustrated in a series of 214 deep orthopaedic fracture infections, in which 58% occurred in the tibia and ankle, and only 10% occurred anywhere in the upper extremity.[18] Finally, pelvic fractures are associated with some of the most challenging SSIs to treat among closed fractures because of their propensity to gram negative organisms and limitations in reconstruction options post-infection. Ultimately, infectious complications in these fracture populations lead to prolonged morbidity, loss of function, and potential limb loss.[1]

1.2 Prevention of Infection

The prevention of infection is the single most important goal influencing peri-operative care of patients with fractures that require surgical management. Standard practice in the management of extremity fractures includes sterile technique and pre-operative skin cleaning with an antiseptic solution. The available solutions kill bacteria and decrease the quantity of native skin flora, thereby decreasing SSI.[19–22] While there is extensive guidance on specific procedures for prophylactic antibiotic use and standards for sterile technique, the evidence regarding the choice of antiseptic skin preparation solution is very limited for extremity fracture surgery.

1.3 Rationale for Pre-Operative Antiseptic Skin Solution Prophylaxis

The most common skin preparation solutions include either an iodophor or chlorhexidine-based active ingredient and are delivered in an alcohol or aqueous-based solution. Iodophors achieve effective antisepsis by penetrating the cell wall of microorganisms and disrupting critical protein and nucleic acid structures.[23] Iodophors are effective against most bacteria, but also may have

broader-spectrum coverage of mycobacteria, viruses, and some spores compared to chlorhexidine gluconate (CHG).[23] CHG similarly achieves antimicrobial effects by penetrating the cell wall of microorganisms. This antimicrobial action allows CHG to be effective against most bacteria.[23]

The evidence guiding pre-operative antiseptic skin solution choice in fracture surgery is largely extrapolated from other surgical disciplines. In a randomized controlled trial involving 849 patients undergoing clean-contaminated abdominal, gynecologic, or urologic surgery, the use of 2% CHG in 70% isopropyl alcohol was compared to aqueous 10% povidone-iodine. The overall rate of 30-day SSI was significantly lower in the CHG in alcohol group compared to the povidone-iodine group (9.5% vs. 16.1%; P=0.004; relative risk, 0.59; 95% confidence interval (CI): 0.41–0.85). While this study demonstrated superior efficacy of CHG in alcohol compared to povidone-iodine, comparing an alcohol based solution to an aqueous solution creates uncertainty about whether the result observed occurred from the superiority of CHG over iodine, isopropyl alcohol over water, or a synergistic combination of CHG in alcohol.[19] In an effort to overcome the controversies associated with comparing CHG and iodine in different solutions, a more recent randomized controlled trial of 1,147 caesarean section patients allocated patients to 2% CHG in 70% isopropyl alcohol versus 8.3% povidone-iodine in 72.5% isopropyl alcohol. Similar to the previous randomized controlled trial, CHG proved more efficacious for reducing 30-day SSI (4.0% in the CHG in alcohol group and 7.3% in the iodine in alcohol group; relative risk, 0.55; 95% CI: 0.34–0.90; P=0.02).[20]

While the evidence from the above two randomized controlled trials demonstrates decreased SSI from CHG solutions in clean-contaminated abdominal and genito-urinary surgery, a larger non-randomized trial reported opposite effectiveness results. Swenson *et al.,* completed a larger 3,209 patient pragmatic sequential implementation study, in which the use of the preoperative skin antiseptic solution was changed after six-month periods.[21] In this study, there were three treatment periods, each with approximately 1,000 general surgery patients undergoing elective and emergent cases. In the first period, patients received 7.5% povidone-iodine scrub, 70% isopropyl alcohol scrub, and 10% povidone-iodine skin paint. The second group received 2% CHG in 70% isopropyl alcohol (CHG group), and the third group received 0.7% iodine povacrylex in 74% isopropyl alcohol. Adjusted comparisons were performed using the intention-to-treat (ITT) principle and an as-treated analysis. Lower SSI rates were seen in the povidone-iodine skin paint group (4.8%) and the iodine povacrylex in isopropyl alcohol group (4.8%), compared with the SSI rates in the 2% CHG in 70% isopropyl alcohol group (8.2%) ($P< 0.05$; povidone-iodine skin paint odds ratio: 0.56, 95% CI: 0.40–0.79).[21] While the results of the Swenson study contradict those of the smaller randomized controlled trials, this large pragmatic study further highlights that the choice of antiseptic skin solution affects SSIs, and data to select the best solution remain conflicting.

Considering the conflicting data, the most recent Cochrane systematic review comparing the efficacy of pre-operative antiseptic skin solutions for clean surgery concluded, "investment in at least one large trial (in terms of participants) is warranted to add definitive and hopefully conclusive data to the current evidence base. Ideally any future trial would evaluate the iodine-containing and CHG-containing solutions relevant to current practice…"[24] The Cochrane recommendation is a direct response to the limitations of the current available literature comparing antiseptic skin solutions. For orthopaedic fracture surgery, the impact of the treatment

uncertainty is further magnified when considering the higher rates of SSIs among open fracture patients and patients with closed lower extremity and pelvic fractures.

1.4 Extrapolating Evidence from Other Surgical Disciplines to Fracture Surgery is Problematic

With regards to orthopaedic patients, the inconsistent results leave the optimal antiseptic solution in doubt; in addition, results may differ across surgical settings. The risk of SSI is substantially greater in certain fracture populations (open fractures, closed lower extremity fractures, and pelvic fractures) due to the soft tissue trauma, wound contamination in open fractures, the increased risk of local vascular disruption, and the required surgery to fix the broken bones. Furthermore, the emergent nature of fracture surgery means that patients are unable to undergo other prophylactic skin care, such as CHG bathing, which is rendered to elective cases to reduce SSI. Additionally, the timing of prophylactic antibiotics may also fall beyond the recommended windows due to delays in getting to hospital; therefore, local antisepsis may become even more critical.

Most important, the soft tissue injury associated with a fracture is a critical difference from elective abdominal or gynecologic surgery. Other differences include wound contamination in open fractures, the use of a tourniquet that decreases the blood flow to the limb (potentially increasing the risk of infection), and the additional risk of implanting metal fixation that can harbor bacteria. Swenson *et al.,* directly acknowledged that the studies performed in general surgery patients may not apply to other specialties, particularly orthopaedic surgery.[21] Even if one wanted to directly apply the conflicting results outlined above to the care of fractures, there are critical limitations in the sparse general surgery and obstetrical literature available.

The most significant limitation in the existing literature is the use of a 30-day endpoint for SSI in all three studies described above.[19–21] While this may be acceptable for identifying most SSIs that involve only the skin (superficial SSI), infections that occur deep to the muscle and around the bone (deep SSI and organ/space SSI) often present beyond 30-days post-injury and have significantly more morbidity and mortality than superficial SSIs. This is a major limitation to the external validity of the previous studies' ability to guide fracture fixation practice. In the FLOW open fracture trial, nearly half the infection-related complications were identified between 30 and 90 days from injury.[4] Similarly, a large case series of patients who developed deep infections following fracture fixation found that post-operative infections occurred at an average of 77 days after surgery (range, 3 days to 51 weeks).[18] While infections occurred earlier in patients with closed fractures, a substantial proportion occurred beyond 90 days (**Figure 1**).[18] Not only does the existing literature not extend follow-up during this period, it is plausible that the treatment effects of the antiseptic solutions behave differently for preventing deep or organ/space infections that often present between 30 and 90 days post-surgery. The need for longer follow-up is supported by a mandatory 90-day surveillance period for deep and organ/space SSIs according to the Centers for Disease Control and Prevention (CDC).[25] Therefore, the lack of directly applicable evidence, an overall paucity of good clinical evidence, and the inadequate duration of outcome follow-up mandate the need for a large, rigorous clinical trial in surgical preparation solutions in fracture care.

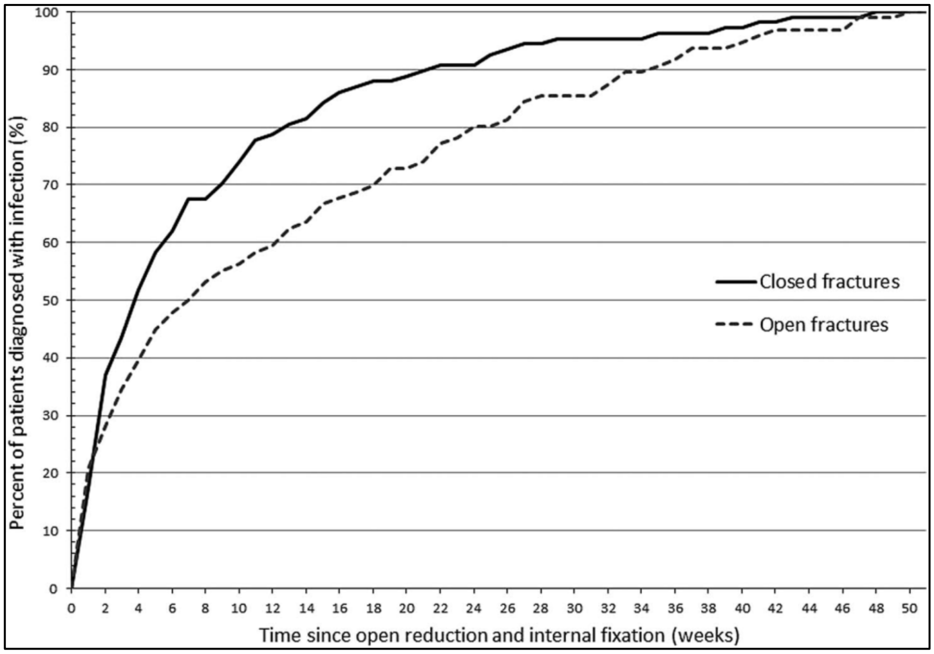

Figure 1: Time from Fracture Management Surgery Until SSI in Open and Closed Fractures

1.5 Why Iodophor Skin Preparations May Reduce Operative Extremity Fracture SSI

The only surgical skin preparation effectiveness data available for extremity fracture surgery come from the FLOW trial.[4] Secondary multivariable analyses of 2,447 patients with open fractures found that when compared to CHG solutions, iodophor-based skin antiseptic preparation solutions could be protective against complications (Adjusted Hazard Ratio 0.88, 95% CI: 0.69–1.12).[4] However, the wide CI suggests iodophor solutions may reduce the odds of infection by as much as 31% or increase it by as much as 12%, leaving its superiority as a fracture care skin preparation solution unresolved.[4]

There are several chemical properties to suggest iodine povacrylex may be more effective than CHG at preventing extremity fracture SSI.[23] Firstly, iodine has a potentially broader spectrum of antimicrobial activity.[23] Secondly, many open fracture patients require repeat surgical debridement, and therefore, these patients will receive multiple exposures to the pre-operative antiseptic solution. Extended use of iodophors has not been associated with the selection of resistant bacterial strains, whereas bacterial resistance to CHG has been documented.[23,26,27] While the methods for detecting CHG resistance are challenging and its clinical significance remains uncertain, these early observations heighten interest in establishing the comparative effectiveness of iodophors versus CHG. Finally, iodine povacrylex dries to form a water-insoluble polymer-based film that increases its resistance to being washed away by saline and bodily fluids.[21] This increased tissue adherence may contribute to increased antisepsis longevity compared to CHG solutions.

1.6 Why Iodophor Skin Preparations May Reduce Extremity Fracture Reoperations

While the primary rationale for using antiseptic skin preparation solutions is to reduce the risk of SSI, many fracture healing complications are associated with indolent infections. These low-grade infections typically do not exhibit clinical signs consistent with SSI. Instead, they present several months post-fracture fixation and are only detected from deep tissue samples collected during secondary surgeries to treat fractures that fail to heal (nonunion). Previous fracture

nonunion studies have identified an infectious etiology in 31–38% of cases.[28,29] Similarly, results from the FLOW trial suggest that 58% of the reoperation events were caused by fracture nonunion or a hardware failure related to an infection, wound-healing problem, or bone-healing problem (n= 188/323). This is consistent with the data presented in **Figure 1**, confirming a high proportion of fracture infections requiring surgery occurred beyond the 90-day surveillance period for SSI.[18] Therefore, given the rationale that iodophors may be more effective in preventing SSI, it is clinically plausible that its use may also reduce unplanned fracture-related reoperations.

1.7 Lack of Surgeon Consensus

The FLOW trial demonstrated a clear divide among orthopaedic surgeons regarding their choice to use the two most common antiseptic solutions during open fracture fixation surgery.[4] Iodophor solutions were used in 54% of the surgeries performed, while 41% were performed using CHG solutions. The remaining surgeons either used both iodophor and CHG (4%), or alcohol with no iodophor or CHG (1%).[4] Building upon the lack of consensus among orthopaedic surgeons participating in the FLOW trial, our research team conducted an internet-based survey (n = 210) and several interviews with orthopaedic surgeons to understand the reasons for the lack of consensus in the use of surgical preparation solutions. Similar to the observations of the FLOW trial, there was nearly an equal split between the use of iodophor and CHG solutions in open and closed fracture surgery. More insight was gained in interviews with the surgeons. Three main drivers for surgeon decision-making were identified: 1) they continued to use the antiseptic solution shown to them during their surgical training, 2) they used the solution recommended by their hospital, or 3) they felt the tissue toxicity was less with their chosen solution. No surgeon could cite a clinical study that helped guide their decision, despite all surgeons indicating they believed the antiseptic solution was important for reducing their patient's risk of SSI. Limited consensus among surgeons reflects a lack of compelling evidence on the optimal approaches to surgical skin preparation, further vindicating the need for a large definitive trial.

The PREPARE Trial, A Pragmatic Randomized trial Evaluating Pre-operative Alcohol skin solutions in FRactured Extremities, will address these gaps in the literature.

2.0 STUDY OBJECTIVES AND HYPOTHESES

2.1 Study Objectives and Hypotheses

The overarching objective of this trial is to compare the effectiveness of iodine povacrylex (0.7% free iodine) in 74% isopropyl alcohol versus 2% CHG in 70% isopropyl alcohol for the management of extremity fractures that require surgical treatment. Open and closed fracture participants will be recruited separately to compare the independent effectiveness of the study solutions in each population. *SSI* will be the primary outcome for comparing effectiveness (primary objective), and *unplanned fracture-related reoperation* will be the secondary outcome for comparison (secondary objective). While previous randomized controlled trials in general surgery and gynecology demonstrated superior efficacy of CHG in alcohol solutions to reduce SSIs,[19,20] results from larger populations of general surgery patients and the recently completed FLOW trial[4] suggest iodophor-based solutions could be more effective than CHG in fracture patients. Therefore, we hypothesize that iodine-povacrylex is a more effective pre-operative antiseptic skin solution than CHG to reduce 90-day SSIs and unplanned fracture-related reoperations within one year of injury.

2.2 Open and Closed Fracture Study Populations

Open fracture patients and closed fracture patients represent two distinct populations within extremity fracture surgery. Open fractures are associated with wound complications that are approximately four times greater than closed fractures.[4,5] The increased baseline risk, differing fracture treatment principles, and the distinct difference of having deep tissue exposed to micro-organisms at the time of injury provides a biologic rationale for maintaining separate open and closed fracture populations. This rationale is further strengthened by data collected from our surgeon survey that suggests many surgeons use different antiseptic skin prophylaxis procedures for open and closed fracture surgeries. Therefore, definitively comparing the effectiveness of the study solutions in each fracture population addresses distinctly different treatment decisions for surgeons. Similarly, if a difference in the effectiveness between the two study solutions were detected in only one of the fracture populations this would be an independently important clinical finding that would have an immediate effect on clinical practice.

2.3 Subgroup Objectives

The PREPARE trial will also explore the possibility of differential treatment effects of the pre-operative antiseptic skin solutions among clinically important subgroups within each independent fracture population.

2.3.1 Open Fracture Subgroups

The open fracture subgroups will be defined by i) the severity of open fracture (Gustilo-Anderson type I or II versus III);[1] ii) upper extremity versus lower extremity open fractures; iii) severity of wound contamination; and iv) presence or absence of comorbidities that affect wound healing.

2.3.2 Closed Fracture Subgroups

The closed fracture subgroups will be defined by: i) severity of soft tissue injury (Tscherne grade 3 versus grades 0-2), and ii) presence or absence of comorbidities that affect wound healing.

2.3.3 Subgroup Hypotheses

It has been established that several patient and injury factors are frequently associated with worse patient outcomes after extremity fractures.[30,31] As a result, we hypothesize that iodine povacrylex (0.7% free iodine) in 74% isopropyl alcohol will be associated with a larger reduction in odds for SSI and unplanned fracture-related reoperations among patients with a higher risk for extremity fracture SSI. Specifically, in both the open and closed fracture populations we expect to observe this heterogeneity of treatment effect in patients with more severe soft tissue injury and patients with increased comorbidities due to the potentially broader antimicrobial coverage, stronger tissue adherence, and increased antiseptic longevity of iodine povacrylex.[32] The credibility of all subgroup analyses will be assessed in accordance with criteria outlined by Sun et al.[33]

Within the open fracture population, high-grade soft tissue injury (Gustilo-Anderson type III), lower extremity open fractures, and moderate/severe wound contamination are established predictors of SSI and reoperations from the FLOW trial.[5] In addition, there are known differences in patients' skin flora based on anatomic region of injury. As a result, it is likely that the study interventions may be more effective in certain open fracture subgroups. Due to its broader spectrum of antimicrobial activity, the increased effectiveness observed by Swenson *et al.*, and the possible benefits observed in the FLOW trial, we hypothesize that the iodine povacrylex (0.7% free iodine) in 74% isopropyl alcohol antiseptic skin solution will be

associated with a larger reduction in odds for SSI and reoperation in open fracture patients with Gustilo-Anderson type III fractures, lower extremity fractures, and more severely contaminated wounds.

3.0 TRIAL DESIGN

3.1 Summary

This study is a multi-center pragmatic cluster randomized crossover trial with two independent populations of surgically treated fracture participants: 1) the open fracture population consisting of a minimum of 1,540 participants with open extremity fractures; and, 2) the closed fracture population of a minimum of 6,280 participants with closed lower extremity or pelvic fractures. The unit of randomization is the orthopaedic practices within clinical sites (clusters), with individual patients being the unit of analysis. The procedures for enrollment, study interventions, follow-up, and analyses within the open and closed fracture populations will follow the same protocol (with noted differences as applicable). Recruitment for each treatment group will be performed in multiple iterations of approximately two-month periods. Each orthopaedic practice will initially be randomized to use one of two pre-operative surgical skin preparation solutions (iodine povacrylex (0.7% free iodine) in 74% isopropyl alcohol versus 2% CHG in 70% isopropyl alcohol) for open and closed extremity fracture surgeries at their institution (**Figure 2**). Upon completion of the two-month period, each orthopaedic practice will crossover to the alternative treatment allocation and complete another two-month recruitment period. This process of alternating treatment periods (crossovers) will continue until the minimum sample size is achieved for each fracture population and the study's budgeted recruitment duration is completed.

Upon completion of recruitment, it is expected that each orthopaedic practice will enroll a minimum of 77 open fracture patients and 314 closed lower extremity or pelvic fracture patients per treatment (a minimum of 154 open fracture patients and 628 closed lower extremity or pelvic fracture patients in total) as applicable, and that most clinical sites will exceed this minimum recruitment goal. Clinical site personnel will screen potential patients for eligibility, and if eligible, they will be invited to participate in the trial. Study participants will be assessed at regular intervals in the one year following their fracture. The primary outcome will include any SSI event from the time of fracture to the end of the 30- and 90-day post-operative periods from their definitive fracture management surgery. The secondary outcome will include unplanned fracture-related reoperations that occur within one-year of their fracture. A blinded Adjudication Committee will review SSIs and unplanned fracture-related reoperations to confirm that they meet the criteria for being a study event.

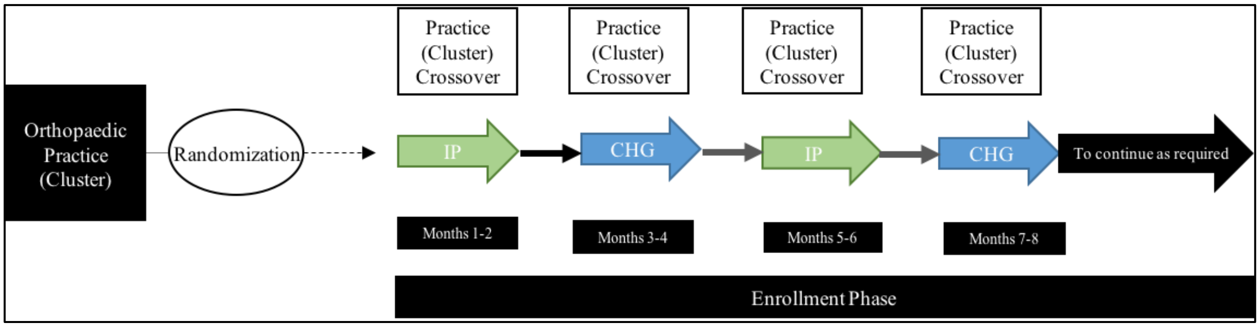

Figure 2: Randomized Treatment Allocation, Cluster Crossover, and Recruitment

3.2 Pragmatic-Explanatory Continuum

In accordance with recommended methodology standards, we have used the PRagmatic-Explanatory Continuum Indicator Summary (PRECIS-2) toolkit to evaluate the PREPARE trial design decisions to determine whether these decisions will lead to a study that answers, “Does this intervention work under usual conditions?” (pragmatic) versus “Can this intervention work under ideal conditions?” (explanatory). The PRECIS-2 tool uses a 5-point Likert scale in 9 domains to evaluate the continuum of design choices. A domain score of 5 indicates “very pragmatic,” while a score of 1 suggests “very explanatory.” **Table 1** outlines the investigators’ assessment of the trial design and the rationale for each assessed score and **Figure 3** displays the PRECIS-2 wheel.

Table 1: PRECIS-2 Score

Domain	Score	Rationale
Eligibility	5	Eligibility criteria are very broad and include all fracture patients that would be treated in all hospital environments.
Recruitment	5	Recruitment of all consenting fracture patients treated at each participating hospital will be performed.
Setting	4	Recruitment is occurring at multiple sites across the US and Canada; however, since most of the recruiting hospitals are regional referral centers the setting is “mostly pragmatic.”
Organization	5	The interventions do not need an increase in providers or care delivery compared to the usual antiseptic care provided. For each antiseptic solution, a brief in-service training session will be provided to the clinical sites, as per any new product/procedure that is being introduced into an operating room.
Flexibility (delivery)	5	The interventions will be delivered in the usual care manner with no advice on allowed co-interventions or strict protocols to ensure compliance.
Flexibility (adherence)	-	This section is left blank according to PRECIS-2 guidance because the intervention is provided prior to patient consent and individual patient compliance is not an issue. If provider adherence is considered, the study design is rather pragmatic (4) because there will be limited encouragement to follow the manufacturer’s directions for use, other than periodic newsletters, investigator meetings, and possible provider survey during the recruitment period.
Follow-up	5	All study follow-up is consistent with usual care.
Primary outcome	5	The outcome has been validated by patients as being very relevant to the study participants and it does not require specialized expertise beyond the treating physician for diagnosis.
Primary analysis	5	All available study data will be used for analysis following the intention to treat principle.

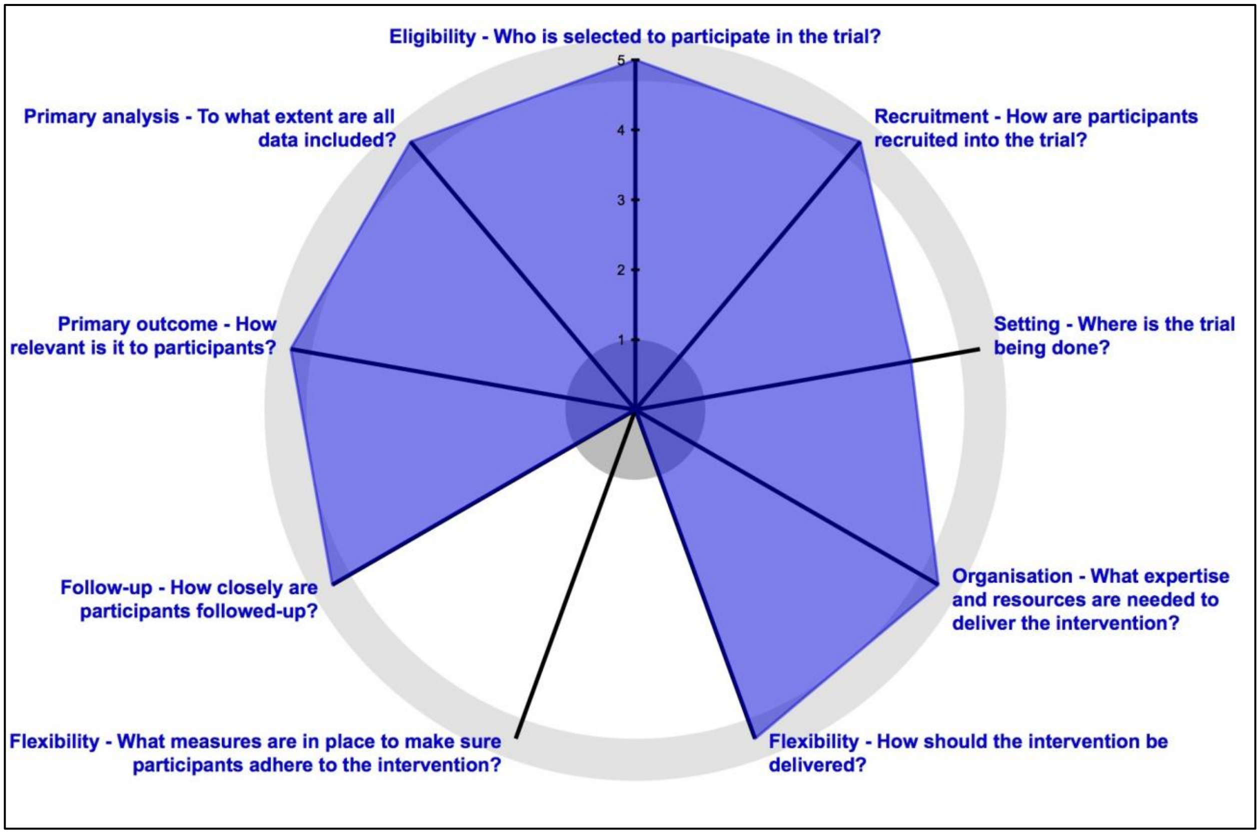

Figure 3: PRECIS-2 Wheel

4.0 METHODS

4.1 Study Setting, Cluster Eligibility, and Selection of Clusters

This study will be coordinated by the Methods Center at the Center for Evidence-Based Orthopaedics (CEO), McMaster University, Hamilton, Ontario and by the Administrative Center within the Department of Orthopaedics at the University of Maryland School of Medicine, R Adams Cowley Shock Trauma Center, Baltimore, Maryland. Patients will be enrolled from at least 18 clinical sites in North America. Clusters (orthopaedic practices within clinical sites) will be carefully screened prior to participation in the PREPARE trial. Clinical site inclusion criteria are: 1) adequate research personnel infrastructure to manage the study; 2) adequate open fracture volume and closed lower extremity and pelvic fracture volume to complete enrollment within the study timeline (i.e., a minimum of 77 open fractures and 314 closed lower extremity fractures per year); 3) commitment from all or most orthopaedic surgeons to participate in the trial; and 4) ability to use the two alcohol-based skin preparation solutions. The exclusion criteria are: 1) lack of interest in the trial; 2) anticipated challenges with complying with the protocol; 3) conflicting studies, in the judgment of the Principal Investigators, that would inhibit patient participation; and 4) budgeting or contract constraints.

The screening process will begin with potential clinical sites completing a feasibility questionnaire that asks about research experience and infrastructure, fracture volume, current practice patterns, and interest in participating in the trial. Clinical sites that meet the eligibility criteria at this stage will be invited to participate in a series of teleconferences to review study and clinical logistics in detail with members of the study team. The Principal Investigators and

study personnel will further vet the clinical sites during these calls and will ask about hospital and patient demographics to ensure that a variety of fracture patient populations and referral patterns, ranging from large urban trauma centers to smaller referral hospitals, are included in the PREPARE trial. Study personnel will document reasons for clinical site ineligibility. Upon selection, clinical sites will be asked to complete a questionnaire that will detail current surgeon preferences and practices for pre-operative surgical preparation techniques and co-interventions known to influence the incidence of SSIs (see Section 4.7).

4.2 Eligibility Criteria

Broad eligibility criteria will be used to increase the generalizability of the trial. Potential participants will be enrolled into only one of the study populations depending on whether they meet the open or closed fracture population criteria (Section 4.2.1 or 4.2.2). Participants who meet the initial criteria for both populations will be assigned to a study population based on the criteria outlined in Section 4.2.3.

4.2.1 Open Fracture Population Eligibility Criteria

The open fracture inclusion criteria are:

1. Patients 18 years of age or older.
2. Open fracture of the appendicular skeleton.
3. Received or will receive definitive fracture treatment with a surgical implant(s) (i.e., internal fixation, external fixation, joint prosthesis, etc.).
4. Open fracture wound management that includes formal surgical debridement within 72 hours of their injury.
5. Will have all planned fracture care surgeries performed by a participating surgeon or delegate.
6. Informed consent obtained.
7. Patient enrolled within 3 weeks of their fracture.

The open fracture exclusion criteria are:

1. Fracture of the hand (distal to radial carpal joint).
2. Patients who did not or will not receive the allocated pre-operative surgical preparation solution due to a medical contraindication.
3. Received previous surgical debridement or management of their fracture at a non-participating hospital or clinic (as applicable).
4. Open fracture managed outside of the participating orthopaedic service (e.g., foot fracture managed by podiatrist).
5. Chronic or acute infection at or near the fracture site at the time of initial fracture surgery.
6. Burns at the fracture site.
7. Incarceration.
8. Expected injury survival of less than 90 days.
9. Terminal illness with expected survival less than 90 days.
10. Currently enrolled in a study that does not permit co-enrollment.
11. Unable to obtain informed consent due to language barriers.
12. Likely problems, in the judgment of study personnel, with maintaining follow-up with the patient.
13. Prior or current enrollment in a PREP-IT trial.
14. Enrolled in the PREPARE closed cohort.
15. Excluded due to sampling strategy.

4.2.2 Closed Fracture Population Eligibility Criteria

The closed fracture inclusion criteria are:

1. Patients 18 years of age or older.
2. Closed fracture of the lower extremity or pelvis.
3. Received or will receive definitive fracture treatment with a surgical implant(s) (i.e., internal fixation, external fixation, joint prosthesis, etc.).
4. Fracture management requires a surgical incision (i.e., for fracture reduction or implant insertion).
5. Will have all planned fracture care surgeries performed by a participating surgeon or delegate.
6. Informed consent obtained.
7. Patient enrolled within 6 weeks of their fracture.

The closed fracture exclusion criteria are:

1. Patients who did not or will not receive the allocated pre-operative surgical preparation solution due to a medical contraindication.
2. Received previous surgical management of their fracture at a non-participating hospital or clinic.
3. Fracture managed outside of the participating orthopaedic service (e.g., foot fracture managed by podiatrist).
4. Chronic or acute infection at or near the fracture site at the time of initial fracture surgery.
5. Burns at the fracture site.
6. Incarceration.
7. Expected injury survival of less than 90 days.
8. Terminal illness with expected survival less than 90 days.
9. Currently enrolled in a study that does not permit co-enrollment.
10. Unable to obtain informed consent due to language barriers.
11. Likely, problems, in the judgment of study personnel, with maintaining follow-up with the patient.
12. Prior or current enrollment in a PREP-IT trial.
13. Enrolled in the PREPARE open cohort.
14. Excluded due to sampling strategy.

4.2.3 Additional Eligibility Considerations

1. Patients with multiple fractures will be eligible for inclusion.
    a. In patients with one or more open and one or more closed fractures, study personnel will determine whether the participant will be enrolled in the open or closed fracture population. This will be determined by identifying the fracture with the highest anticipated risk of SSI. In most cases, the open fracture will be selected, and the participant will be designated to the open fracture population; however, it is possible that a closed lower extremity fracture may have a higher anticipated SSI risk compared to an open fracture. A plausible example would be a Tscherne grade 3 closed intra-articular distal tibia fracture versus a Gustilo-Anderson type I open distal radius fracture.
    b. Once the participant is designated to the applicable open or closed fracture population, study personnel will collect data on up to three eligible fracture regions. If a participant is in the open fracture population, then only eligible

regions with open fractures will be included. Similarly, only closed lower extremity or pelvic fracture regions will be included if the participant is in the closed fracture population. In patients with more than three eligible fracture regions, the treating surgeon will determine the three regions with the most severe fractures.

c. For each fracture, the entire injured anatomic region will be included.[34] Therefore, if there are two fractures that anatomically communicate, they will be considered within the same region (e.g., within the shoulder region, forearm, etc.). For open fracture participants, adjacent closed fractures that anatomically communicate with an open fracture or are treated within the same surgical incision will be included in the open fracture region. Common examples of these include forearm fractures, tibia/fibula fractures, and peri-articular fractures. The anatomic joint region, adjacent fractures, and contiguous wounds will be defined at the time of patient enrollment on the case report forms (CRFs).

d. All included fracture regions should be treated with the same allocated antiseptic skin solution as per the cluster randomization.

2. At the time of screening, patients who are in another study who meet eligibility criteria are to be included in the PREPARE trial unless the other trial does not permit co-enrollment.
3. Closed fractures that are definitively managed without a surgical incision will be excluded (e.g., stab incisions and pin sites) because a localized stab wound or pin site infection does not meet the CDC definition for SSI. If there is an associated surgical incision, these fractures will be included (e.g., open reduction and fixation with an external fixator, k-wires, etc.).

4.3 Recruitment Strategy

4.3.1 Patient Screening & Consent

Patients 18 years of age or older who present to a recruiting hospital for treatment of an open fracture of the appendicular skeleton will be screened for participation within 3 weeks of their fracture. Patients 18 years of age or older who present to a recruiting hospital for surgical treatment of a closed lower extremity or pelvic fracture(s) will be screened for participation within 6 weeks of their fracture. To screen patients for eligibility, designated study personnel at each clinical site will develop a patient enrollment plan. This plan will typically consist of daily participation in orthopaedic patient rounds and a review of daily listings of hospital admissions for patients with open fractures and or closed lower extremity or pelvic fractures. Upon identification, the study personnel will screen the patient for eligibility and if eligible, approach them for informed consent. Study participants with open fractures must be enrolled within 3 weeks of their fracture(s) and study participants with closed fractures must be enrolled within 6 weeks of their fracture(s). Enrollment may take place at any time within this window. If the patient is unable to provide informed consent (e.g., due to their injury) at the time they were initially identified, informed consent may be delayed until they are able to provide informed consent. Alternatively, if the patient is unable to provide informed consent, informed consent may be obtained from their proxy, with consent obtained from the patient when/if the patient is able to provide consent. Allowing informed consent from a patient's proxy healthcare decision maker will reduce the risk of recruitment bias against the most severely injured patients. In addition, potentially eligible patients will be approached to participate in the trial, even if they did not receive the correct pre-operative antiseptic skin solution. This is consistent with the ITT principle and is necessary to maintain the

prognostic balance achieved during the cluster randomization. All screened patients will be classified as included, excluded, or missed. See **Table 2** below for the Schedule of Events.

Table 2: Schedule of Events

**Assessment**	**Visit 1:** Enrollment	**Visit 2:** 6 weeks post-fracture	**Visit 3:** 3 months post-fracture	**Visit 4:** 6 months post-fracture	**Visit 5:** 9 months post-fracture	**Visit 6** 12 months post-fracture
Eligibility Screening	●					
Informed Consent	●					
Collection of Demographic and Fracture Characteristics Data	●					
Collection of Surgical Data	●					
Collection of Peri-Operative Data	●					
Collection SSI Data	●	●	●	●	●	●
Collection of Reoperation Data	●	●	●	●	●	●
Collection of SAE Data	●	●	●	●	●	●

For patients with open fractures, informed consent and enrollment must occur within the 3 weeks (21 days) from the patient's fracture (Day 0 is the date of the fracture). For patients with closed fractures, informed consent and enrollment must occur within the 6 weeks (42 days) from the patient's fracture (Day 0 is the date of the fracture). Visits are to be completed at routine clinic visits. When necessary, visits may also be completed by telephone, text, email, standard mail, and/or a review of the participant's medical record.
Follow-up visit windows touch so that participants will always fall into a specific window. The windows are: 4 to 8 weeks (i.e., 28 to 56 days), 2 to 4.5 months (i.e., 57 to 137 days), 4.5 to 7.5 months (i.e., 138 to 228 days), 7.5 to 12 months (i.e., 229 to 365 days), and greater than 12 months (366 to 730 days), respectively, from the participant's fracture.

4.4 Managing Patient Volume

When the volume of eligible patients exceeds a participating site's ability to effectively enroll and follow all eligible patients, two strategies are available to manage patient volume and ensure that enrollment targets are met. Clinical sites may obtain permission from the Methods Centre to use either one or both of these strategies.

4.4.1 Enrollment of Patients from Only One Fracture Cohort

To manage patient volume, clinical sites may obtain permission from the Methods Centre to only enroll patients from one fracture cohort (i.e., open fracture cohort or closed fracture cohort), as opposed to both. When this strategy is used, clinical sites will only approach patients from the fracture population selected (i.e., they will only enroll patients with open fractures or only enroll patients with closed fractures). The Methods Centre will work with clinical sites to determine the fracture population from which patients should be recruited. Additionally, sites with competing studies may also enroll into one fracture cohort only. For example, clinical sites who

are participating in the Aqueous-PREP trial (sister trial to PREPARE), may participate in PREPARE by enrolling patients into the closed fracture cohort alone.

4.4.2 Enrollment Sampling Plan

A sampling strategy is available within the REDCap Cloud electronic data capture (EDC) system which will randomly determine whether an eligible patient should be approached for consent and inclusion in the study. The randomization software will use randomly selected block sizes consistent with the sampling ratio being used during the recruitment periods. Examples of potential random sampling strategies a site may use include:

1. For every three eligible patients, there will be one excluded eligible patient (3:1 ratio).
2. For every two eligible patients, there will be one excluded eligible patient (2:1 ratio).
3. For each eligible patient, there will be one excluded eligible patient (1:1 ratio).
4. For each eligible patient, there will be two excluded eligible patients (1:2 ratio).
5. For each eligible patient, there will be three excluded eligible patients (1:3 ratio).

The number of eligible patients approached for consent and inclusion in the study, and the number of eligible patients that are excluded due to a sampling strategy will be documented in the EDC system.

For sites enrolling patients from both open and closed fracture populations, the enrollment sampling plan may differ between the open and closed fracture populations. Therefore, it is possible that a recruiting cluster may achieve their overall enrollment goal sooner in one population than the other. If this occurs, Methods Center personnel may instruct the recruiting cluster to stop enrollment of the completed population and continue enrollment of only the other fracture population. This decision will be made based on the overall study recruitment, timelines, and other logistical concerns.

4.4 Randomization Methods

Treatment allocation will be determined using a cluster-randomized crossover trial design. The order of treatment allocation for each orthopaedic practice (cluster) will be randomly assigned using a computer-generated randomization table. Each site will start with the initially allocated study solution and crossover to the other solution for their second recruitment period. Both the open fracture and closed lower extremity and pelvic fracture populations will receive the same treatment allocation and follow the same crossover schedule. The process of alternating treatments will repeat approximately every two months as dictated by the initial randomization. For sites that enroll for more than 1 year, the order of treatment allocation may be reversed after 12 months to ensure equal distribution of each treatment across each calendar month in the study's duration (**Figure 2**). Randomization will be completed by personnel at the CEO Methods Center at the onset of the trial. Personnel from the Methods Center will notify personnel at each participating clinical site of their treatment allocation order. This will allow each participating clinical site to begin preparing for the first run-in period.

4.5 Blinding

The orthopaedic team (including the study coordinators) cannot be blinded to the treatment allocation as the antiseptic solutions are visually distinguishable and these individuals need to lead the implementation of the cluster-crossover protocol at their clinical site. The Adjudication Committee Members and data analysts will be blinded to the study treatment. All interpretations

of study results will initially be done in a blinded manner by developing two interpretations of the results. One interpretation will assume treatment A is iodine povacrylex (0.7% free iodine) in 74% isopropyl alcohol, the other interpretation will assume it is 2% CHG in 70% isopropyl alcohol. Once the data interpretations for each assumption are finalized, the data will be unblinded and the correct interpretation will be accepted.[35]

4.6 Description of the Interventions

4.6.1 Initial Run-In Phase

Prior to initiating patient recruitment, each clinical site will begin using their assigned pre-operative antiseptic skin solution for eligible fracture surgeries (run-in period) to ensure that acceptable compliance is met before initiating participant enrollment. Acceptable compliance during the run-in phase will be defined as at least 15 eligible open fracture patients and at least 15 closed lower extremity or pelvic fractures patients with >90% of eligible patients receiving the allocated antiseptic solution or a minimum of one month in duration. The run-in phase may be extended up to 3 months, as deemed necessary by the CEO Methods Center. Study personnel at each clinical site will document compliance with administering the allocated treatment during the run-in phase and submit this weekly to the CEO Methods Center. Specifically, the weekly reports will include the total number of eligible operative patients within the open fracture population and the closed fracture population, the proportion who received the assigned pre-operative antiseptic skin solution, and the proportion who did not receive the assigned pre-operative antiseptic skin solution along with details about the deviations (e.g., name of attending surgeon, solution used, rationale for not using the assigned pre-operative antiseptic skin solution). This portion of the study protocol is for quality assurance during the initial implementation of the trial procedures. Fracture surgeries reviewed during the run-in phase will not be included in the trial. Similarly, these patients will not be approached for informed consent and no individual patient-level data will be submitted. CEO Methods Center personnel will review the weekly reports with each of the clinical sites and develop strategies, as needed, to ensure acceptable compliance during the run-in phase. This weekly communication will prevent any delays in transitioning to the participant enrollment phase.

4.6.2 First Intervention Phase

Once the initial run-in phase is completed, participant recruitment will begin with the clinical sites continuing to use the same pre-operative antiseptic skin solution for all eligible fracture surgeries within the open and closed fracture populations over a two-month period. Patients will receive the initially allocated treatment solution for all of their fracture management surgeries, including repeat planned surgeries, even if a planned subsequent surgery occurs during a recruitment period using the non-allocated solution. Participating clusters will ideally be able to enroll a minimum of 77 open fracture patients and 314 closed lower extremity and pelvic fractures per treatment over the total study recruitment duration (total of 154 open fracture patients and 628 closed lower extremity and pelvic fracture patients), and it is anticipated that most recruiting centers will exceed this minimum goal. Methods Center personnel will continue to monitor compliance with the assigned pre-operative antiseptic skin solution over the enrollment phase and work collaboratively with the clinical sites to minimize cases in which a patient receives the incorrect solution. These monitoring activities will coincide with site-specific procedures to maintain compliance for all patients, even those requiring multiple surgical procedures. All assessments of compliance will be analyzed separately for the open and closed fracture populations. If a fracture requires multiple surgeries and the correct solution is not

applied at each procedure, the patient will remain in the study and be analyzed using the allocated solution (ITT principle).

4.6.3 Second Intervention Phase

Once the first intervention phase is completed, each site will crossover to the opposite study solution. This crossover will occur simultaneously in the open and closed fracture populations. There will be no run-in phase for the second solution and each site will need to develop local procedures to ensure a successful crossover. Example procedures to minimize carry-forward of first solution into the second solution phase include: 1) removing the bottles of the first solution from the orthopaedic operating rooms; 2) changing study posters and notifications within the operating rooms; and 3) performing the crossover during the middle of the week to provide a few days' notice to the operating room staff and to avoid contamination of recent fracture patients returning for repeat procedures (e.g., weekend admissions). The enrollment goals and procedures will mirror the first intervention phase. Methods Center personnel will continue to monitor compliance with the assigned pre-operative antiseptic skin solution over the enrollment phase and work collaboratively with the clinical sites to reduce the risk of contamination.

4.6.4 Special Considerations for Ongoing Treatment Crossovers

Treatment allocation will continue to alternate between the study solutions, as outlined above, for the remainder of study duration. Each intervention phase will be approximately two months in duration, as agreed upon by the clinical site and CEO Methods Center personnel. The duration may be modified to avoid crossovers on holidays, weekends, and other circumstances that could threaten a successful crossover. The expected recruitment duration for the trial is approximately 24 months; however, some sites may have a shorter total recruitment duration (e.g., a participating site that joins the trial later, high volume clinical sites, etc.). The two-month enrollment periods will help account for seasonal variability in SSI incidence and their associated infectious organisms,[36] as each crossover period will cover a season. In addition, for those clinical sites enrolling beyond 12 months, the distribution of recruitment periods for each solution may be seasonally matched by reversing the order of the alternating allocation after 12 months of recruitment.

4.6.5 Evaluation of Site Performance and Removal of Clinical Sites

After every two recruitment periods (approximately every four months), each site will be evaluated for continued participation in the trial. Sites with <90% of eligible patients receiving the allocated solution, differential adherence between study solutions, <95% follow-up of the primary outcome, <90% follow-up of the secondary outcome, incomplete data submission, or other threats to data quality or the validity of the study may be withdrawn from the trial. In the event a site is withdrawn, data collection will be completed for all enrolled participants and these data will be included in the final study analysis.

4.6.6 Application of Pre-Operative Antiseptic Skin Solutions

Each solution will be applied to the skin and allowed to dry for a minimum of three minutes. While the application and minimum drying time for both study solutions are very similar, local study personnel will provide standardized in-service (training) for orthopaedic surgeons, operating room technicians, and nurses at each participating hospital prior to the initial run-in phase. This training should include reviewing the manufacturers' directions for use to help minimize incorrect application at clinical sites that may not routinely use both solutions. In

addition, the manufacturers may also provide demonstration videos and posters for continued refresher training for each solution.

The study protocol will mandate the antiseptic skin solution to be used in each intervention phase (Sections 4.4, 4.6.1, 4.6.2, 4.6.3, and 4.6.4); however, the protocol will remain pragmatic to variability in the actual application of the solutions and other co-intervention steps performed during the entire pre-operative skin preparation process performed in the operating room. Based on individual surgeon preference, this often includes mechanically removing visible dirt or debris with a scrub brush, and/or cleaning the limb with isopropyl alcohol or an antiseptic scrub solution. These additional skin preparation steps will be permitted provided that: 1) the final skin preparation step prior to surgical incision is the application of the allocated antiseptic solution; and, 2) participating surgeons continue to use the same skin preparation co-interventions in both intervention phases. Co-interventions that contain the opposite active ingredient from the current intervention phase (e.g., using a CHG scrub brush during the iodine intervention phase, or conversely, using an iodine scrub during the CHG intervention phase) should be avoided; however, deviations from this recommendation will be permitted to maintain pragmatic *flexibility of delivery* and reflect real-world clinical practice. The details of all operating room antiseptic co-interventions will be documented.

4.6.7 Patients with Multiple Planned Surgeries

Fracture patients who require multiple planned surgeries for their injury will receive the same antiseptic skin solution during each subsequent procedure. Methods Center personnel will work with each of the clinical sites to develop strategies for minimizing crossovers. For example, for patients who require multiple surgeries and are enrolled within 14 days of the anticipated end of a recruitment period, study personnel will develop local procedures to identify these patients as study participants and indicate the patient's allocated antiseptic solution in the medical chart and CRFs.

4.6.8 Iodophor Antiseptic Solution

The iodine-based treatment intervention will be an antiseptic solution comprised of iodine povacrylex (0.7% free iodine) in 74% isopropyl alcohol. 3M™ DuraPrep™ [3M Health Care, St Paul, MN], will be the commercial product used. Clinical site personnel will store and handle the product as per the manufacturers' recommendations. Operating room personnel will apply the solution to the operative site as the final preoperative skin antisepsis preparation immediately prior to commencing surgical fixation. They will apply the solution as per manufacturer's directions for use (e.g., technique of application, duration of application, drying time, drying techniques, replacement of draping, etc.).

4.6.9 CHG Antiseptic Solution

The CHG solution will contain 2% CHG in 70% isopropyl alcohol as the only active ingredients. Products that list other inactive ingredients will be permitted. ChloraPrep® [CareFusion Inc., Leawood, KS, USA] will be the commercial product used. Clinical site personnel will store and handle the product as per the manufacturers' recommendations. Operating room personnel will apply the solution to the operative site as the final preoperative skin antisepsis preparation immediately prior to commencing surgical fixation. They will apply the solution as per manufacturer's directions (e.g., technique of application, duration of application, drying time, replacement of draping, etc.).

4.7 Perioperative Co-Interventions

To optimize the internal validity of the trial findings, key details of co-interventions known to influence the incidence of SSIs will be documented. Hospitals typically implement standard procedures to achieve quality process benchmarks designed to minimize SSIs. These benchmarks are outlined in several similar guidelines such as the Joint Commission's Surgical Care Improvement Project 10 Core Measures to prevent SSI, the Society for Healthcare Epidemiology of America compendium to prevent SSI, and prevention guides from the Institute for Healthcare Improvement and the Association of periOperative Registered Nurses. While these guidelines mandate core benchmark processes to minimize SSI, it is not practical or generalizable for the trial protocol to standardize the steps taken or co-interventions performed to achieve these core measures, since each participating hospital will already have their own implemented procedures. This is the primary rationale for the cluster-crossover design, in which each participating hospital will act as its own control for the effect of co-interventions. Therefore, four key approaches to account for and limit the potential differential application of co-interventions during the study periods will be performed: 1) study periods for each intervention are kept relatively short to improve the likelihood that newly implemented co-interventions will be equally distributed across both treatment solutions; 2) encourage participating hospitals not to make changes to their existing infection prevention interventions during the study periods; 3) document the co-interventions being used in the hospitals throughout the study periods; and 4) record any changes in co-interventions that do occur if mandated by a participating hospital's administration. To this end, a monitoring tool containing a list of commonly applied prophylactic co-interventions being used at the participating clinical sites will be completed approximately every four months to document any changes to their infection prevention strategies during the study period.

4.8 Outcome Measures

<u>4.8.1 Primary Outcome</u>

The primary outcome is SSI, guided by the CDC's National Healthcare Safety Network reporting criteria (2017),[25] which includes superficial incisional SSI within 30 days and deep incisional or organ/space SSI within 90 days of fracture surgery (**Table 3**). Since the management of some fractures may have more than one operative procedure as part of an intentionally staged surgical plan (e.g., multiple irrigation and debridements, wound closures, temporary stabilization surgeries, definitive fixation surgery), the primary outcome will include any SSI event from the date of fracture to the end of the 30- and 90-day post-operative surveillance periods from their definitive fracture management surgery. For participants with multiple fracture regions, the date of the definitive fracture management surgery will be matched to the fracture region with the SSI.

Table 3: CDC Surgical Site Infection Criteria

Outcome	Description
Superficial Incisional SSI	Date of event for infection occurs from the date of fracture to 30 days after the definitive fracture management surgery (where day 1 = the procedure date)<br>**AND**<br>involves only skin and subcutaneous tissue of the incision<br>**AND**<br>patient has at least one of the following:<br>a. purulent drainage from the superficial incision.<br>b. organisms identified from an aseptically-obtained specimen from the superficial incision or subcutaneous tissue by a culture or non-culture based microbiologic testing method which is performed for purposes of clinical diagnosis or treatment (e.g., not Active Surveillance Culture/Testing [ASC/AST]).

Outcome	Description
	c. superficial incision that is deliberately opened by a surgeon, attending physician or other designee and culture or non-culture based testing is not performed.<br>**AND**<br>patient has at least one of the following signs or symptoms: pain or tenderness; localized swelling; erythema; or heat.<br>d. diagnosis of a superficial incisional SSI by the surgeon or attending physician or other designee.<br>The following do not qualify as criteria for meeting the definition of superficial SSI:<br>• Diagnosis/treatment of cellulitis (redness/warmth/swelling), by itself, does not meet criterion "d" for superficial incisional SSI. Conversely, an incision that is draining or that has organisms identified by culture or non-culture based testing is not considered a cellulitis.<br>• A stitch abscess alone (minimal inflammation and discharge confined to the points of suture penetration).<br>• A localized stab wound or pin site infection- Such an infection might be considered either a skin (SKIN) or soft tissue (ST) infection, depending on its depth, but not an SSI<br>Note: A laparoscopic trocar site for an operative procedure is not considered a stab wound.<br>• An infected burn wound is classified as BURN and is not an SSI.
Deep Incisional SSI	The date of event for infection occurs from the date of fracture to 90 days after the definitive fracture management surgery (where day 1 = the procedure date)<br>**AND**<br>involves deep soft tissues of the incision (e.g., fascial and muscle layers)<br>**AND**<br>patient has at least one of the following:<br>a. purulent drainage from the deep incision.<br>b. a deep incision that spontaneously dehisces, or is deliberately opened or aspirated by a surgeon, attending physician or other designee, and organism is identified by a culture or non-culture based microbiologic testing method which is performed for purposes of clinical diagnosis or treatment (e.g., not Active Surveillance Culture/Testing [ASC/AST]) or culture or non-culture based microbiologic testing method is not performed<br>**AND**<br>patient has at least one of the following signs or symptoms: fever (>38°C); localized pain or tenderness. A culture or non-culture based test that has a negative finding does not meet this criterion.<br>c. an abscess or other evidence of infection involving the deep incision that is detected on gross anatomical or histopathologic exam, or imaging test
Organ/Space SSI	Date of event for infection occurs from the date of fracture to 90 days after the definitive fracture management surgery (where day 1 = the procedure date)<br>**AND**<br>infection involves any part of the body deeper than the fascial/muscle layers, that is opened or manipulated during the operative procedure<br>**AND**<br>patient has at least one of the following:<br>a. purulent drainage from a drain that is placed into the organ/space (e.g., closed suction drainage system, open drain, T-tube drain, CT guided drainage)<br>b. organisms are identified from an aseptically-obtained fluid or tissue in the organ/space by a culture or non-culture based microbiologic testing method which is performed for purposes of clinical diagnosis or treatment (e.g., not Active Surveillance Culture/Testing [ASC/AST]).<br>c. an abscess or other evidence of infection involving the organ/space that is detected on gross anatomical or histopathologic exam, or imaging test evidence suggestive of infection.<br>**AND**<br>meets at least one criterion for a specific organ/space infection site listed in Table 3 of the CDC Procedure-associated Module (summarized in Table 4 below).[25] These criteria are found in the Surveillance Definitions for Specific Types of Infections chapter.[37]

*The CDC criteria has been modified to include all definitive fracture management surgeries, as opposed to including only National Healthcare Safety Network procedures that require infection reporting.

The CDC criteria for classifying SSIs will be followed. If multiple tissue levels are involved in the infection, the type of SSI (superficial incisional, deep incisional, or organ/space) reported will reflect the deepest tissue layer involved in the infection during the surveillance period. The date of event will be the date that the participant met criteria for the deepest level of infection using the following procedures: 1) report infection that involves the organ/space as an organ/space SSI, whether or not it also involves the superficial or deep incision sites and 2) report infection that involves the superficial and deep incisional sites as a deep incisional SSI. The most relevant National Healthcare Safety Network Organ/Space SSI classifications are summarized in **Table 4**. Whenever possible, the treating surgeon or study personnel should take photos of the infected region to facilitate the adjudication process.

Table 4: Relevant Organ/Space SSI Sites

Organ/Space SSI	BONE	Osteomyelitis
	JNT	Joint or bursa infection
	PJI	Prosthetic joint infection

All reported SSIs will be reviewed independently by an infection preventionist nurse and an orthopaedic surgeon who are members of the Adjudication Committee. Briefly, they will complete the review by examining all relevant information to determine if the SSI meets the CDC criteria of a superficial incisional SSI, deep incisional SSI, or organ/space SSI. The Committee will reach consensus on all reviewed SSIs. A hospital epidemiologist and infectious disease physician who are members of the Adjudication Committee will be available to provide guidance as needed. All members of the Adjudication Committee will be blinded to the treatment allocation.

4.8.2 Secondary Outcome

The secondary outcome is unplanned fracture-related reoperation within 12 months of the fracture(s). This outcome has been used in previous fracture trials and is defined as any unplanned surgery that occurred from the time of injury to 12 months post-injury that is associated with an infection at the operative site or contiguous to it, a wound-healing problem, or a fracture delayed union or nonunion. Common examples include any unplanned: 1) irrigation and debridement of surgical incisions or open fracture wounds due to infections or wound healing problems; 2) revision wound closure for dehiscence; 3) soft tissue coverage procedure for infected or necrotic wound; 4) fracture delayed union or nonunion surgery (such as bone grafting or implant exchange); and 5) reoperation for hardware or prosthesis failure due to infection or bone-healing problems. Removal of hardware for soft tissue prominence or periprosthetic fracture are common examples of reoperations that will not be considered outcome events. To facilitate adjudication, the treating surgeon or study personnel should take photographs of any infections or wound infections. Two orthopaedic surgeons who are members of the Adjudication Committee will independently review all reported unplanned fracture-related reoperations to determine if they meet the criteria for being a study event. The Committee will reach consensus on all reviewed unplanned fracture-related reoperations.

4.8.3 Exploratory Outcomes

Two exploratory definitions of infection will be used for sensitivity analyses of the primary comparison. The first exploratory outcome is fracture-related infection (FRI) within 12 months of the fracture, defined by the confirmatory criteria for FRI outlined in a 2018 consensus definition.[38] The FRI criteria has been selected as an exploratory outcome because the CDC criteria has been criticized for failing to adequately account for the complexities of infections in traumatic fractures.[38,39] The FRI criteria attempts to improve upon the ability to detect infections specifically in fracture patients; however, this definition of FRI has not been fully validated or widely adopted.

The confirmatory criteria include the presence of one or more of the following signs/symptoms:

1) Fistula, sinus or wound breakdown (with communication to the bone or the implant).
2) Purulent drainage from the wound or presence of pus during surgery.
3) Phenotypically indistinguishable pathogens identified by culture from at least two separate deep tissue/implant (including sonication-fluid) specimens taken during an operative intervention. In case of tissue, multiple specimens (3) should be taken, each with clean instruments (not superficial or sinus tract swabs). In cases of joint effusion, arising in a joint adjacent to a fractured bone, fluid samples obtained by sterile puncture may be included as a single sample.
4) Presence of microorganisms in deep tissue taken during an operative intervention, as confirmed by histopathological examination using specific staining techniques for bacteria or fungi.

The second exploratory outcome is SSI using the CDC criteria *within 12 months of the fracture*. This secondary outcome will use the same diagnostic CDC reporting criteria for the primary outcome (**Tables 3 and 4**); however, the timeframe for this outcome will be expanded to include all SSIs that occur within 12 months of fracture. Similar to the rationale for using the FRI outcome, and the recommendations for a minimum of 12 months follow-up for orthopaedic fracture outcomes[40], this expanded timeframe will detect infections that occur beyond the standard CDC surveillance reporting periods. This modification of the CDC reporting periods has been used in previous orthopaedic fracture trials.[4,41]

An infection preventionist nurse and an orthopaedic surgeon member of the Adjudication Committee will review all reported SSIs to determine if they meet the FRI confirmatory criteria and / or the CDC criteria following the processes described above (see Section 4.8.1).

4.8.4 Data Collection and Participant Follow-up

After obtaining informed consent, study personnel will record the baseline data on the study CRFs. They will obtain this information directly from the participant or proxy, from the participant's medical chart, and the participant's treating orthopaedic surgeon or other health care providers. Data collection points include participant characteristics and injury details such as age, gender, comorbidities, mechanism of injury, and other injuries. Study personnel will also record the characteristics of up to three eligible fracture regions including the bone(s) fractured, fracture severity, size of the wound (if applicable), and degree of soft tissue injury using the Tscherne classification in closed fractures and the Gustilo classification in open fractures.[1,34,36]

Surgical data and in-hospital data will be collected throughout the participant's hospital stay. Detailed information will be collected regarding the surgical management of their fracture(s),

including the timing of the surgery(ies) and the method of initial and definitive fracture treatment. For open fracture regions, study personnel will also record the use of staged debridements, the presence or lack of skin closure between debridements, and the use of local antibiotics at the wound. Lastly, study personnel will record the use of negative pressure wound therapy for open wounds or in the presence of open wounds surgically closed. These treatment decisions are hypothesized to be associated markers of injury severity and potential confounders of the study interventions.

Study participants will be followed at 6 weeks, 3 months, 6 months, 9 months, and 12 months from their fracture. SSIs and unplanned fracture-related reoperations will be identified at the time of diagnosis/occurrence and/or during each participant's clinical assessment and medical record review that will occur during their routine outpatient clinic visits (**Table 2**). Detailed information on the SSI including the date of diagnosis, participant signs and symptoms, culture test results, method of treatment(s), and date of resolution will be collected. Study personnel will also record details about the participants' reoperations on the CRFs (e.g., date of reoperation, type of procedure, reason for procedure, etc.). In cases where the participant does not return to the clinic, study personnel will contact the participant by telephone, text, email, and standard mail and will review their medical record for any SSIs or fracture-related reoperations. If the participant reports being treated at another hospital, study personnel will obtain the medical records from the other hospital. We have used this approach in our other multi-center trials (e.g., SPRINT, TRUST, FLOW, FAITH, HEALTH, etc.).[4,41–44]

To ensure research participant safety, serious adverse events (SAEs) will be documented at each follow-up visit and promptly submitted to the Methods Center and the local or central Institutional Review Board (IRB) or Research Ethics Board (REB) as per the required reporting processes.

Several strategies may be used to maximize follow-up including: 1) at the time of enrollment, each participant will provide their own telephone number, as well as the name and address of a primary care physician, and the names and phone numbers of three people at different addresses with whom the participant does not live with and who are likely to be aware of the participant's whereabouts; 2) participants will receive a reminder card upon discharge for their next follow up visit by the clinical site study personnel; 3) participants will receive text message reminders; 4) follow-up will coincide with normal surgical fracture clinic visits; and 5) if a participant refuses or is unable to return for the follow-up assessment, study personnel will determine his/her status with regard to major study outcomes by telephone, text, mail, or email contact with the participant or the provided alternate contacts. Given these are standard of care visits and the participants will be receiving ongoing orthopaedic care for their acute fractures, minimal loss to follow-up is expected. Using these techniques, we expect greater than 95% follow-up at 3 months and 90% follow-up at 12 months post-fracture.

Participants will not be deemed lost to follow-up until the 12-month visit is overdue and all attempts to contact the participant have been exhausted. Participants will not be withdrawn from the study if the study protocol was not adhered to (e.g., allocated treatment not received, missed follow-up visits, etc.). The reasons for participants being withdrawn from the study will be documented (e.g., withdrawal of consent or lost to follow up).

5.0 STATISTICAL PLAN

5.1 Sample Size Determination

The overall objective of the trial is to determine the most effective alcohol-based pre-operative antiseptic skin solution for use during extremity fracture management. This objective is being performed independently in the open and closed fracture populations. In both fracture populations, the analyses will compare the effectiveness of iodine povacrylex (0.7% free iodine) in 74% isopropyl alcohol versus 2% CHG in 70% isopropyl alcohol surgical skin preparations. The primary outcome is the occurrence of SSI, as per the adapted CDC criteria (**Table 3**).[25] The secondary outcome is the occurrence of unplanned fracture-related reoperations within 12 months of injury. Separate sample size estimates for the open and closed fracture populations were calculated to facilitate the primary comparison between proportions of patients with SSI in each treatment group. It is expected that this estimate will also provide adequate power for the secondary outcome (unplanned fracture-related reoperation) because a meaningful effect size for the reoperation outcome is expected to be similar to the SSI estimates. Additionally, the baseline risk of unplanned reoperations in both fracture populations is expected to be greater than the risk of SSI.[4]

Assuming an ITT principle for the analysis, the sample size was calculated based on a cluster crossover design with the cluster as the unit of randomization and the patient as the unit of analysis. For complex study designs, such as a cluster-randomized crossover trial, simple formulas to calculate sample size or power may not capture the expected variability from the observed data.[45] Simulation methods were used to obtain empirical power calculations based on a feasible number of recruiting clusters and the expected number of participants within the open and closed fracture populations.[45] The simulation estimates are designed to detect a difference between the treatment groups, accounting for between hospital variability inherent to a cluster-crossover trial design.

We have estimated the CHG group will experience a SSI incidence of 12.5% in the open fracture population and a 3.5% incidence within the closed fracture population.[3,4] Compared to CHG, we have assumed the iodine povacrylex solution will achieve a 0.65 risk reduction for SSI and unplanned fracture-related reoperation in each fracture population.[22] This effect was selected as the smallest difference that would be important to detect, in the sense that any smaller effect would not be of clinical or substantive importance. Additionally, this effect was deemed more conservative than data reported by Swenson et al. and was consistent with feasible recruitment goals.[21]

We have based our sample size assumptions using a single crossover, 2-period design to ensure the most conservative sample size estimate. Recent simulation data suggest that increasing the number of period crossovers can increase the statistical power of a given sample size.[46] The initial power estimate assumed 10 recruiting clusters, a 10% loss to follow-up rate,[4] and applying the between-cluster variance of 0.095 observed in the FLOW trial. Based on enrollment of a minimum of 1,540 open fracture patients and 6,280 closed lower extremity and pelvic fractures, greater than 80% power would be achieved for each fracture population. Subsequent to the initial power calculations, the early trial experience demonstrated a need to increase the number of clusters to obtain a feasible recruitment pace. As a result, a minimum 18 clusters will enroll participants into PREPARE. The increase in clusters results in a marginal increase in power (~2%).

Table 5 and **Table 6** below outlines the summary of the initial sample size assumptions. These sample size estimates are rounded up to the nearest multiple of 20 to ensure balance among 10 clinical sites and two interventions.

Table 5: Sample Size Assumptions for Open Fractures

Baseline SSI Risk	Iodine Risk Ratio	Iodine Odds Ratio	Sample Size	Sample Size Increased by 10%
10.0%	0.62	0.59	1,600	1,760
10.0%	0.65	0.63	1,960	2,100
10.0%	0.67	0.65	2,200	2,420
10.0%	0.70	0.68	2,600	2,860
12.5%	0.62	0.59	1,300	1,440
12.5%	0.65	0.62	1,400	1,540
12.5%	0.67	0.64	1,600	1,760
12.5%	0.70	0.67	1,800	1,980
14.0%	0.62	0.58	1,200	1,320
14.0%	0.65	0.61	1,300	1,440
14.0%	0.67	0.64	1,500	1,660
14.0%	0.70	0.67	1,800	1,980

Note: Between cluster ICC = 0.028; Between cluster variance = 0.095; Between period variance = 0; Number of clusters = 10; Number of periods = 2; Alpha = 0.05

Table 6: Sample Size Assumptions for Closed Fractures of the Lower Extremity

Baseline SSI Risk	Iodine Relative Risk	Iodine Odds Ratio	Sample size	Sample Size increased by 10%
2%	0.62	0.62	8,200	9,020
2%	0.65	0.65	10,000	11,000
2%	0.67	0.67	11,400	12,540
3.5%	0.62	0.61	4,700	5,170
3.5%	0.65	0.64	5,700	6,280
3.5%	0.67	0.66	6,600	7,260
5%	0.62	0.61	3,300	3,640
5%	0.65	0.64	4,100	4,520
5%	0.67	0.67	4,300	4,740

Note: Between cluster ICC = 0.028; Between cluster variance[*] = 0.095; Between period variance = 0; Number of clusters = 10; Number of periods = 2; Alpha = 0.05

5.2 Statistical Methods

5.2.1 Analysis Plan Overview

A detailed statistical analysis plan will be published prior to the completion of the trial. The following analysis plan will be conducted independently for the open and closed fracture populations. For each population, the analyses and reporting of the results will follow the CONSORT guidelines for reporting of both pragmatic trials[47] and cluster-randomized trials.[48] The process of participant enrollment and flow throughout the study will be summarized using a flow-diagram. Participant demographics and baseline outcome variables will be summarized using descriptive summary measures expressed as mean (standard deviation) or median (interquartile range) for continuous variables depending on the distribution, and number (percent) for categorical variables.[49] An ITT principle will be adopted to analyze all outcomes and the unit of analysis will be the individual participants. Missing data will be assumed to be missing at random and will be handled with multiple imputation.[50,51]

The primary analysis will compare the treatment groups using the SSI outcome and the secondary analysis will compare the unplanned fracture-related reoperation outcome. The secondary comparison will be conducted in accordance with best practice guidelines for secondary analyses. For all models, the results will be expressed as relative measure of effect (odds, risk, or hazard ratios) and corresponding two-sided 95% confidence intervals.

5.2.2 Analysis of the Study Outcomes

Adopting an ITT principle, multilevel regression models will be used. Correlation structures will be fit based on the observed between cluster and between period effects. A robust sandwich estimator will be used to analyze the primary and secondary outcomes.

For the primary outcome, SSI will be the dependent variable and the antiseptic solution (treatment group) will be the independent variable. For the secondary outcome, unplanned fracture-related reoperation will be the dependent variable and the antiseptic solution (treatment group) will be the independent variable. For both analyses, multiple imputation will be used to handle missing data.[51]

As the optimal methods for analyzing cluster crossover trials continue to evolve, the final statistical modeling technique to be used will be determined in accordance with contemporary best practices prior to the completion of participant follow-up. A separate Statistical Analysis Plan will be developed prior to study closeout. **Table 7** below shows a summary of the study outcomes, corresponding hypotheses, and currently proposed methods of analysis.

Table 7: Summary of Outcome Analysis Plan

Objective	Outcome		Hypothesis	Method of Analysis
	Name	Type		
To determine the effect of iodine-based versus CHG-based pre-operative antiseptic skin solutions on the incidence of SSI and unplanned fracture-related reoperation.	SSI	Binary	Iodine solution will be more effective than CHG solution	Multi-level regression model
To determine the effect of iodine-based versus CHG-based pre-operative antiseptic skin solutions on the incidence of SSI and unplanned fracture-related reoperation.	Unplanned Fracture-Related Reoperation	Binary	Iodine solution will be more effective than CHG solution	Multi-level regression model

Note: CHG = chlorhexidine gluconate; SSI = Surgical Site Infection

5.2.3 Subgroup Analyses

A limited number of *a priori* subgroup analyses will be performed. The open fracture subgroups will include: i) severity of open fracture wound (Gustilo-Anderson type I or II versus III);[1] ii) upper extremity versus lower extremity open fractures; iii) none, minimal, or surface contamination versus contamination embedded in bone or deep soft tissues;[34] and, iv) presence or absence of comorbidities that affect wound healing.

The closed fracture subgroups of interest include: i) severe soft tissue injury (Tscherne Grade 3 versus Tscherne Grade 0-2) and, ii) presence or absence of comorbidities that affect wound healing. These analyses will be performed by comparing the effect estimates in both groups (interaction effect). We hypothesize that effect will differ by subgroup. These analyses will be

approached and reported in accordance with best practices and guidelines for subgroup analyses.[33,52–54] **Table 8** below shows a summary of the subgroup analysis objectives, corresponding outcomes, hypotheses, and methods of analysis for each fracture population.

Table 8: Summary of Subgroup Analysis Plan

Objective	Outcome		Hypothesis	Method of Analysis
	Name	Type		
Open Fracture Subgroup Analyses				
Severity of open fracture (Gustilo-Anderson Type I or II vs. Type III)	SSI / Unplanned Fracture-Related Reoperation	Binary	Iodine solution will be associated with a larger reduction in odds for SSI and reoperation than CHG solution in more severe fractures	Interaction of treatment by subgroup
Upper extremity vs. lower extremity fractures	SSI / Unplanned Fracture-Related Reoperation	Binary	Iodine solution will be associated with a larger reduction in odds for SSI and reoperation than CHG solution in lower extremity compared to upper extremity fractures	Interaction of treatment by subgroup
None, minimal, or surface wound contamination vs. embedded wound contamination	SSI / Unplanned Fracture-Related Reoperation	Binary	Iodine solution will be associated with a larger reduction in odds for SSI and reoperation than CHG solution in embedded contaminated wounds compared to wounds with no, minimal or surface contamination	Interaction of treatment by subgroup
Presence or absence of comorbidities that affect wound healing	SSI / Unplanned Fracture-Related Reoperation	Binary	Iodine solution will be associated with a larger reduction in odds for SSI and reoperation than CHG solution in participants who have comorbidities that affect wound healing	Interaction of treatment by subgroup
Closed Fracture Subgroup Analyses				
Severe soft tissue injuries (Tscherne Grade 3 vs. Tscherne Grade 0-2)	SSI / Unplanned Fracture-Related Reoperation	Binary	Iodine solution will be associated with a larger reduction in odds for SSI and reoperation than CHG solution in fractures with severe soft tissue injuries	Interaction of treatment by subgroup
Presence or absence of comorbidities that affect wound healing	SSI / Unplanned Fracture-Related Reoperation	Binary	Iodine solution will be associated with a larger reduction in odds for SSI and reoperation than CHG solution in participants who have comorbidities that affect wound healing	Interaction of treatment by subgroup

Note: CHG = chlorhexidine gluconate; SSI = Surgical Site Infection

5.2.4 Sensitivity Analyses

Assessment of the sensitivity or robustness of the findings to the key assumptions is essential in trials. The following sensitivity analyses may be conducted to explore the effects of alternative analysis models, alternative missing data approaches, balancing prognostic imbalance, as-treated analyses, variability in co-interventions, and alternative definitions of SSI.

1. *Using different analysis models*: There are several methods for analyzing cluster randomized crossover trials.[51,55] Therefore, our sensitivity analyses will explore alternative multi-level models with different correlation structures for the error.[52,55]

2. *Different methods of handling missing data*: There are several methods of handling missing data in trials.[55] Multiple imputation assumes that the data are missing at random—an assumption that is not verifiable in practice. Other imputation methods will be used such as worst case scenario to impute missing data and assess the robustness of the results.[56] For the worst case scenario analysis, we will assume that a random sample of participants lost to follow-up experienced a study event. For this sensitivity analysis, the proportion assumed to experience a study event will be equivalent to the upper confidence interval of the observed pooled event rate for each study outcome.

3. *Adjusted analyses for prognostic imbalance*: We will also perform sensitivity analyses that assume prognostic imbalance between the two treatment groups based on the following key variables known to be risk factors for SSI or reoperation after extremity fracture management: soft tissue injury, time from injury to definitive fixation, age, work-related injury, and employment status.[5] For patients with open fractures, these additional risk factors will also be considered: Gustilo fracture type, lower extremity fracture, wound contamination, time from injury to first debridement, antiseptic wound dressing in the emergency department, method of fixation, and wound closure at initial debridement.[5] Adjusted analyses including the above risk factors and treatment group as independent variables will be performed for the SSI and reoperation outcomes.

4. *As-treated analyses*: The proportion of surgical procedures receiving the incorrect, non-allocated antiseptic solution will be reported. "As-treated" sensitivity analyses will be performed using the solution received as the independent variable. For participants that were treated in a single fracture surgery, they will be analyzed using the antiseptic solution received. For participants who received multiple fracture surgeries, two analyses will be performed. First, the antiseptic solution used in their last surgery prior to a study outcome event will define their study treatment. For the second analysis, the antiseptic solution received in the majority of their fracture surgeries will define their study treatment. Participants who were treated with multiple fracture management surgeries, but received equal exposure to both treatment solutions (e.g., one surgery with CHG and one surgery with iodine), will be analyzed within their originally allocated treatment group.

5. *Co-intervention variability*: Selective censoring of one or more clusters and / or treatment periods will be performed to further explore between-cluster and between-period variability identified in the primary and secondary outcome comparisons. These analyses will be used to explore the robustness of the study conclusions in the context of measured practice variations in co-interventions that differ between participating sites and / or evolve over the duration of the study recruitment. Results that are sensitive to the removal of a cluster(s) and / or period(s) will be reported, along with potential clinical hypotheses that are supported by the measured clinical practice variation.

6. *Quantitative pooling of open fracture and closed fracture populations*: We will quantitatively pool the treatment effects from the open and closed fracture populations if

the direction of the effect is consistent across the two populations. The rationale for this sensitivity analysis approach is that a consistent direction of effect in the two populations suggests that the populations and mechanism of effect are similar enough to provide a clinically useful estimate of treatment effect if applied to all surgically treated fractures. If the direction of the effect is in opposite directions, for example, CHG appears to be more effective in closed fractures and iodine povacrylex is more effective in open fractures, then no pooling will be performed. This scenario would suggest that the populations and heterogeneity of treatment effect is too divergent; therefore, a pooled treatment estimate would not be clinically useful since surgeons will continue to view the choice of antiseptic skin solution for open and closed fractures patients as separate treatment decisions.

7. *Exploratory SSI definitions:* The above analyses will be repeated for the primary comparison using the FRI outcome and the CDC definition within 1 year of injury to determine if the study conclusions are sensitive to alternative definitions of SSI.

Table 9 below shows a summary of each potential sensitivity analysis objectives, corresponding outcomes, hypotheses, and methods of analysis.

Table 9: Summary of Sensitivity Analysis Plan

	Objective	Outcome		Hypothesis	Method of Analysis
		Name	Type		
1	Different analysis models	SSI / Reoperation	Binary	Iodine solution will be more effective than CHG solution	Multi-level regression models with different correlation structures
2	Different missing data approach	SSI / Reoperation	Binary	Iodine solution will be more effective than CHG solution	Multi-level regression models with missing data imputed using worst-case scenario
3	Baseline prognostic imbalance	SSI / Reoperation	Binary	Iodine solution will be more effective than CHG solution	Multi-level regression models with prognostic variables & treatment group
4	As-treated analysis	SSI / Reoperation	Binary	Iodine solution will be more effective than CHG solution	Multi-level regression models using "as treated" treatment group
5	Co-intervention variability	SSI / Reoperation	Binary	Cluster- and period-variability is related to co-interventions	Censoring of cluster(s) and/or period(s) with differences in co-interventions
6	Quantitative pooling	SSI / Reoperation	Binary	Iodine solution will be more effective than CHG solution in all fracture patients	Meta-analysis with fixed effects
7	Exploratory SSI definitions	FRI / CDC SSI within 1 year	Binary	Iodine solution will be more effective than CHG solution	Multilevel regression model

Note: CHG = chlorhexidine gluconate; SSI = Surgical Site Infection; FRI = fracture-related infection; CDC = Centers for Disease Control and Prevention

5.2.5 Interim Analysis

No formal interim analyses are planned and the trial will not be stopped early for benefit. The Data and Safety Monitoring Committee (see Section 7.5.6) will review frequent safety reports and will collectively make judgments on the strength of evidence and the absolute magnitude and seriousness of any safety signals.[57] The Data and Safety Monitoring Committee may make recommendations regarding the trial.

6.0 DATA MANAGEMENT

6.1 Case Report Forms and Data Transmission

Clinical sites will be provided with the trial CRFs prior to initiation of enrollment. Research personnel at each clinical site will submit the required data, as detailed on the CRFs, to the Methods Center using the REDCap Cloud electronic data capture system. Clinical site personnel will receive a unique login and password for the REDCap Cloud system and will be able to view and modify data for participants recruited at their clinical site.

6.2 Data Integrity

The REDCap Cloud system uses a variety of mechanisms for checking data at the time of entry including skip logic, range checks, and data type checks. Upon receipt of new data, the personnel at the Methods Center will query all missing, implausible, or inconsistent data. Clinical site personnel will be able to review all open queries in the system and will be required to respond promptly.

7.0 ETHICS AND DISSEMINATION

7.1 Research Ethics Approval

The McMaster University Methods Center and all participating clinical sites will receive REB or IRB approval prior to commencing participant enrollment. A central IRB and local IRBs/REBs will be used based on clinical site logistics. Prior to local commencement of the study, each clinical site will provide the Methods Center with a copy of their ethics approval.

7.2 Consent

In many cluster randomized comparative effectiveness trials, a waiver of consent is obtained from the IRB of Record. The rationale for the waiver of consent is that all patients will receive treatments that are effective and within standards of care, they will receive one of the study treatments as part of their routine care regardless of study participation, the data collection is minimal and obtained from the patient's medical records, the trial involves no more than minimal risk to the patient, and that the waiver of consent will not adversely affect the rights and welfare of the patient. Most of these concepts apply to the current trial, as the PREPARE trial is comparative effectiveness research where patients will receive one of the preoperative antiseptic skin solutions regardless of their participation in the study. Additionally, patients are never included in the decision-making process for the choice of antiseptic preparation solution, and, in most situations, they are not even aware of which solution is used. However, in contrast to many cluster randomized crossover trials, PREPARE trial personnel will need to contact participants directly to collect baseline and outcome data, as this information cannot be reliably obtained

from the patients' medical records. Therefore, study personnel will obtain informed consent from patients prior to data collection. This consent process will allow study participants to be informed about the study rationale and provide consent for ongoing surveillance and data collection.

To increase enrollment and to avoid missing potential study participants, the consent process may take place up to 3 weeks post-fracture for open fracture patients and up to 6 weeks post-fracture for closed fracture patients. Consultation during the study design phase with IRB members and patient advisors confirmed the acceptability of this flexible approach, where consent may be obtained after the intervention. The primary rationale for allowing consent after the intervention is consistent with the waiver of consent principles outlined above, but in addition, the patient and IRB stakeholders recognized that obtaining consent prior to the patient's first surgery could add undue decision making stress to a patient who is awaiting surgical management of a serious extremity injury; allowing consent after their surgery would likely facilitate an improved consent process.

The consent process will typically take place in the patient's hospital room or in the outpatient fracture clinic, either before or after the patient has had surgery(ies) to manage their fracture. If the patient is unable to provide informed consent (e.g., due to their injury, language restrictions) within 3 weeks of their open fracture or 6 weeks of their closed fracture, informed consent will be obtained from their proxy. In addition, if a patient has been discharged from hospital prior to being invited to participate in the study, a delegated member of the clinical care team may initiate the consent process by telephone, as approved by the IRB of Record.

To obtain informed consent, delegated study personnel should follow the below procedures:

- Present study information in a manner that is understandable to the potential participant/proxy.
- Discuss the study with the potential participant/proxy and answer any questions he or she asks.
- Allow the potential participant/proxy an opportunity to discuss participation with their family, friends, or family physician, if desired.
- Confirm that the participant/proxy understands the risks and benefits of participating in the study and that their participation is voluntary.
- Complete and obtain signatures for informed consent form and obtain contact information from the participant/proxy.
- Provide/send the participant/proxy with a paper/electronic copy of the signed consent form.

Consent may be obtained electronically or using pen and paper consent forms, as approved by the IRB of Record. If potential participants are contacted by telephone, documenting written informed consent will involve the following procedures:

- The study team confirms the potential participant's interest in learning more about the study and verifies the mailing address or fax number to which the consent form can be sent.
- A blank consent form is mailed or faxed along with a cover letter that introduces the study and explains when the phone conversation will occur. A stamped, self-addressed

envelope is provided if standard mail is used so the participant can return the signed consent document to the study team.

- After the potential participant has received the document, a member of the study team calls the participant and walks through the entire document over the phone, answering questions and making notes about the participant's questions. Time and date of the conversation should be recorded.
- Once all questions are answered, the participant signs the consent form if they are willing to participate. S/he returns the consent form by mail or fax.
- Once received, the study team member who conducted the consent conversation should sign the consent form and date with today's date. To explain the discrepancy, this individual should also write a note on the consent form stating that the participant's consent was obtained by phone on xx date (the date the participant signed.)
- The participant should receive back a fully-signed copy of the consent form for their records.

The process of obtaining and documenting informed consent will be completed in accordance with local Good Clinical Practice recommendations. Consent procedures and forms, and the communication, transmission and storage of patient data will comply with the IRB of Record requirements for compliance with The Health Insurance Portability and Accountability Act.

Upon providing informed consent, study participants will be followed for 12 months from their fracture. Given the short follow-up time, the need for a regular reassessment of consent will not apply; however, participants may withdraw their consent at any time.

7.3 Confidentiality

Information about study participants will be kept confidential and will be managed in accordance with the below rules:

- All study-related information will be stored securely.
- All study participant information will be stored in locked file cabinets, or locked room, as applicable, and accessible only to study personnel.
- All paper and electronic CRFs will be identified only by a coded participant number.
- All databases will be password protected.

In the event that a participant revokes authorization to collect or use personal health information, the clinical site retains the ability to use all information collected prior to the revocation of participant authorization. For participants who have revoked authorization to collect or use personal health information, attempts should be made to obtain permission to collect at least vital status (i.e., primary outcome data) at the end of their scheduled study period.

7.4 Protocol Amendments

Any amendments to the study protocol which will affect the conduct of the study, impact the safety or benefits to participants or affect the analysis and the interpretation of the safety and efficacy of the intervention under investigation (e.g., changes to the study objectives, study design, sample size, or study procedures) will necessitate a formal amendment to the protocol. Any protocol amendments will be approved by the Principal Investigators and will require approval by the McMaster University REB, the Central IRB, local IRBs/REBs, as well as the Funder (as needed). The Methods Center will also file an amendment to all applicable regulatory

agencies for changes to the protocol made after the original regulatory approval. Clinical sites will also be required to submit amendment requests to their IRB of Record to obtain approval for the amendment and to provide the Methods Center with a copy of this approval. Administrative changes (e.g., minor corrections or clarifications that have no effect on the way the study is conducted) will not need to undergo a formal amendment process.

7.5 Adverse Event Reporting and Definitions

7.5.1 Serious Adverse Event (SAE)

A SAE is any adverse event that is any of the following:

- Fatal
- Life threatening
- Requires or prolongs hospital stay
- Results in persistent or significant disability or incapacity
- A congenital anomaly or birth defect
- An important medical event

7.5.2 Unanticipated Problems Resulting in Risk to Participant or Others

Any incident, experience, or outcome that meets the following criteria:

- Unexpected in nature, severity, or frequency (e.g., not described in study-related documents such as the ethics-approved protocol or consent form, etc.).
- Related or possibly related to participation in the research (i.e., possibly related means there is reasonable possibility that the incident experience or outcome may have been caused by the procedures involved in the research).
- Suggests that the research places participants or others at greater risk of harm (including physical, psychological, economic, or social harm).

7.5.3 Serious Unexpected Adverse Drug Reactions

A serious adverse drug reaction means a noxious and unintended response to a drug that occurs at any dose and that requires in-patient hospitalization or prolongation of existing hospitalization, causes congenital malformation, results in persistent or significant disability or incapacity, is life-threatening or results in death. An adverse drug reaction is considered unexpected when its nature (i.e., specificity or outcome), severity or frequency is either not identified, or is not consistent with the term or description used in the product labelling.

7.5.4 Adverse Event Reporting

Clinical sites are responsible for reporting SAEs and serious unexpected adverse drug reactions immediately to the Methods Center via the REDCap Cloud system. Significant new information on ongoing SAEs should also be provided promptly to the Methods Center via the REDCap Cloud system. Unanticipated problems resulting in risk to participants or others are also to be reported promptly to the Methods Center.

The Methods Center will inform all applicable regulatory agencies of any serious unexpected adverse drug reaction in respect of the drug that has occurred as follows:

(a) if it is neither fatal nor life threatening, within 15 days after becoming aware of the information; and

(b) if it is fatal or life threatening, within seven days after becoming aware of the information.

Within eight days after having informed the regulatory agency of any serious unexpected adverse drug reactions, the Methods Center will submit to the regulatory agency a complete report in respect of that information that includes an assessment of the importance and implication of any findings made.

Adverse drug reactions that are expected or unexpected, but not serious, will not be reported to the regulatory agency, but rather monitored and tracked by the Methods Center. The Methods Center will report to applicable regulatory agencies "expected, serious" adverse drug reactions, where an increase in the rate of occurrence or severity, was judged to be clinically important.

A causality assessment will be undertaken by the Methods Center, together with the responsible investigator for clinical investigation cases, and any case judged as having a reasonable suspected causal relationship to the medicinal product will be reported.

7.5.5 Clinical Site Reporting – IRB and REB

Clinical sites are responsible for reporting SAEs and unanticipated problems resulting in risk to participants or others to their local REB/IRB or the Central IRB in accordance with local reporting requirements. Copies of each report and documentation of ethic board notification and receipt will be kept in the clinical site's study file.

7.5.6 Data and Safety Monitoring Committee

As per the FDA guidance document the *Establishment and Operation of Clinical Trial Data Monitoring Committees for Clinical Trial Sponsors*, a Data and Safety Monitoring Committee will oversee the safety of the trial participants and the overall conduct of the trial. The members of the Data and Safety Monitoring Committee will include two orthopaedic surgeons, an infectious disease expert, a biostatistician and a fracture patient representative. One orthopaedic surgeon will act as the Chair of the Committee. The Data and Safety Monitoring Committee will be responsible for safeguarding the interests of study participants, assessing the safety and efficacy of study procedures, and for monitoring the overall conduct of the study. The Data and Safety Monitoring Committee will frequently review enrollment and demographic summaries, listings of protocol deviations, and summaries and listings of SAEs. They will advise the Principal Investigators and study team on any concerns related to participant safety and trial conduct, and will make recommendations for the study to continue as designed, for study termination, for study continuation with major or minor modifications, or temporary suspension of enrollment until some uncertainty is resolved. We will develop a Data and Safety Monitoring Committee charter to guide the process.

7.6 Dissemination Policy

The results from each fracture population will be submitted for publication regardless of whether there are significant findings. Every attempt will be made to ensure that the amount of time between completion of data collection and release of study findings are minimized.

8.0 SUB-STUDY: PATIENT EXPERIENCES IN THE AQUEOUS-PREP AND PREPARE TRIALS

8.1 Introduction

Patient and stakeholder involvement in the design of randomized controlled trials is increasingly becoming recognized as an essential component of a trial's success.[58,59] Patient and stakeholder

involvement (PSI) has been seen as the paradigm shift from research being done "to" or "for" patients, to research being performed "with" or "by" patients themselves.[60] PSI allows for democratization of the research process and empowering patients throughout the entire research process – from design through to knowledge dissemination.[61] Research has found that patients and stakeholders are motivated to be involved in research for a wide variety of reasons, including a desire to contribute to research for the benefit of others.[62]

Prior research has argued that PSI enhances the focus of clinical trials on outcomes that are relevant to patients themselves, thus increasing the utility of any research findings.[63] Furthermore, PSI has been argued to improve recruitment and retention rates, while raising the quality of research findings and ultimately helping with the dissemination of research findings.[64] Lastly, PSI may be able to improve patient safety when patients are involved in safety reporting in hospital settings.[65]

Despite these findings, a recent systematic review estimates that far less than 1% of clinical trials engage patients in any meaningful or active way.[66] From the onset of the PREP-IT trials (i.e. the Aqueous-PREP and PREPARE trials), the PREP-IT investigators have engaged multiple patient-partners and stakeholders in the design, conduct, and implementation of the PREP-IT trials. One of our engagement goals is to identify ways in which we can better engage with PREP-IT study participants. To support this goal, we seek to learn about PREP-IT participants' experiences within the PREP-IT trials. This knowledge will be used to improve the study team's ability to engage study participants and provide study information in a meaningful and accessible manner. Additionally, the unique design of the PREP-IT trials (e.g., consent after the intervention, minimal follow-up, minimal requirements for participants) provides a novel trial to investigate this question. This led to the current sub-study.

8.2 Rationale and Objectives

One of the mandates of the PREP-IT program is to improve orthopaedic fracture research through meaningful engagement with our patient-partners and stakeholders. The objective of this sub-study is to learn about PREP-IT participants' experiences with participating in the Aqueous-PREP or PREPARE trial. The results of this sub-study will be used to develop strategies to better engage research participants both in the PREP-IT trials as well as in future clinical trials.

8.3 Sub-Study Design

This sub-study will consist of an exit survey that will be given to a subset of participants in the PREP-IT trials. Select clinical sites participating in the Aqueous-PREP and / or PREPARE trial will be invited to participate in the sub-study.

The exit survey is comprised of 14 questions that includes multiple choice and brief open-ended questions. All of the questions use clear and simple language written at or below a grade eight reading level to enhance the validity of results. The survey length has been kept to a minimum to maximize response rate and limit barriers that would affect its proper completion.

The survey was created after reviewing the current literature and with input from the PREP-IT investigators, research coordinators, patient-partners, and stakeholders. Engaging the larger study team follows the PREP-IT philosophy of meaningful engagement, as well as helps to ensure that no vital questions were missed and that the survey wording is clear and easily

understandable to the target audience. The questionnaire was pre-tested on a sample of convenience.

8.4 Survey Participants and Distribution

All potential substudy participants, or their proxies, will be required to provide informed consent specifically for the substudy prior to completing the survey. Informed consent for the substudy may be obtained at the time of enrollment in the Aqueous-PREP or PREPARE trial using procedures described in sections 4.3.1 and 7.2, or in-person at a subsequent follow-up visit or time of survey administration using a pen and paper consent form. The patient or proxy must be provided with a copy of the signed informed consent form. All sites within the United States of America must conduct their consenting process in accordance with HIPAA (Health Insurance Portability and Accountability Act) regulations as approved by their institutions, and sites in Canada must comply with the Personal Information Protection and Electronic Documents Act (PIPEDA).

Clinical sites participating in the sub-study will offer the survey to all eligible participants at the time they complete their one-year follow up visit. The survey will be sent to participants either through mail, email or RedCap Cloud, given to them on paper at a follow-up visit, or administered over the phone, depending on each individual participant's preference. The Research Coordinator may also telephone or text the participant to remind them to complete the exit survey. We will document the number of participants invited to participate in the survey as well as the number of participants who decline participation.

8.5 Data Entry

The exit survey responses will be entered into the Aqueous-PREP / PREPARE trial's electronic data capture (EDC) system.

8.6 Sample Size

Sample size was calculated using a 5% margin of error, with 95% confidence intervals, a potential population of all patients who have completed one year follow up (approximately 1600 patients) and an expected response rate of 50%. With this in mind, a sample size of approximately 310 patients who complete every survey question will be required.[67] As such, the survey will be distributed to all participants at participating clinical sites until our sample size of at least 310 participants is achieved.

8.7 Data Analysis

We will summarize all variables with frequencies and percentages. The short form questions will be coded appropriately based on themes.

8.8 Anticipated Implications of Results

This research serves as an important step towards understanding patients' perspectives as participants in a clinical trial. Additionally, the research may influence how future clinical trials are designed and conducted, with the overall goal of a greater focus on the patient experience and increasing patient involvement in research. Lastly, the results of this sub-study could help the study team to develop aids (e.g., posters, pamphlets, etc.) to improve patients' understanding of clinical research and overall experience with the PREP-IT trials.

9.0 REFERENCES

1. Gustilo R, Merkow R, Templeman D. Management of open fractures. *J Bone Jt Surg*. 1990;72(2):299-304.
2. National Center for Health Statistics. Public Use Microdata File Documentation, National Hospital Ambulatory Medical Care Survey, 2011. *Hyattsville, MD Natl Tech Inf Serv 2011*. https://www.cdc.gov/nchs/data/ahcd/nhamcs_outpatient/2011_opd_web_tables.pdf. Accessed February 28, 2018.
3. Darouiche RO. Treatment of Infections Associated with Surgical Implants. *N Engl J Med*. 2004;35014350:1422-1429.
4. FLOW Investigators. A Trial of Wound Irrigation in the Initial Management of Open Fracture Wounds. *N Engl J Med*. 2015;373(27):2629-2641. doi:10.1056/NEJMoa1508502
5. Gitajn IL, Sprague S, Petrisor B, Jeray KJ, O'Hara N, Nascone J, Bhandari M, Slobogean G, FLOW Investigators. Predictors of Complications in Severe Open Fractures. *J Orthop Trauma*.
6. Edwards JR, Peterson KD, Mu Y, et al. National Healthcare Safety Network (NHSN) report: Data summary for 2006 through 2008, issued December 2009. *Am J Infect Control*. 2009;37(10):783-805. doi:10.1016/j.ajic.2009.10.001
7. Whitehouse JD, Friedman ND, Kirkland KB, Richardson WJ, Sexton DJ. The Impact of Surgical-Site Infections Following Orthopedic Surgery at a Community Hospital and a University Hospital Adverse Quality of Life, Excess Length of Stay, and Extra Cost. *Infect Control Hosp Epidemiol*. 2002;23(04):183-189. doi:10.1086/502033
8. Unpublished Data - FLOW Trial.
9. Schoenfeld AJ, Dunn JC, Belmont PJ. Pelvic, spinal and extremity wounds among combat-specific personnel serving in Iraq and Afghanistan (2003-2011): A new paradigm in military musculoskeletal medicine. *Injury*. 2013;44(12):1866-1870. doi:10.1016/j.injury.2013.08.001
10. Molina CS, Stinner DJ, Fras AR, Evans JM. Course of treatment and rate of successful salvage following the diagnosis of deep infection in patients treated for pilon fractures (AO/OTA: 43). *J Orthop*. 2015;12:S18-S24. doi:10.1016/j.jor.2015.01.023
11. Colman M, Wright A, Gruen G, Siska P, Pape H-C, Tarkin I. Prolonged operative time increases infection rate in tibial plateau fractures. *Injury*. 2013;4(2):249-252. doi:10.1016/j.injury.2012.10.032
12. Lin S, Mauffrey C, Mark H, Stahel PF, Hak DJ. Surgical site infection after open reduction and internal fixation of tibial plateau fractures. *Eur J Orthop Surg Traumatol 24797–803*. doi:10.1007/s00590-013-1252-8
13. Morris, BJ, Unger, RZ, Archer, KR, Mathis, SL, Perdue, AM, Obremskey W. Risk Factors of Infection After ORIF of Bicondylar Tibial Plateau Fractures. *J Orthop Trauma*. 2013;27(9).
14. Momaya AM, Hlavacek J, Etier B, et al. Risk factors for infection after operative fixation of Tibial plateau fractures. *Injury*. 2016;24:1501-1505. doi:10.1016/j.injury.2016.04.011
15. Wyrsch B, McFerran MA, McAndrew M, Limbird TJ, Harper MC, Johnson KD SH. Operative Treatment of Fractures of the Tibial Plafond. *JBJS*. 1996;78-A(11).
16. Claessen FMAP, Braun Y, Van Leeuwen WF, Dyer GS, Van Den Bekerom MPJ, Ring D. What Factors are Associated With a Surgical Site Infection After Operative Treatment of an Elbow Fracture? *Clin Orthop Relat Res*. 1999;474:562-570. doi:10.1007/s11999-015-4523-3
17. Farragos AF, Schemitsch EH, McKee MD. Complications of Intramedullary Nailing for

Fractures of the Humeral Shaft: A Review. *J Orthop Trauma*. 1999;13(4):258-267.
18. Torbert JT, Joshi M, Moraff A, et al. Current Bacterial Speciation and Antibiotic Resistance in Deep Infections After Operative Fixation of Fractures. *J Orthop Trauma*. 2015;29(1):7-17. doi:10.1097/BOT.0000000000000158
19. Darouiche RO, Wall Jr. MJ, Itani KMF, et al. Chlorhexidine-Alcohol versus Povidone-Iodine for Surgical-Site Antisepsis. *N Engl J Med*. 2010;362(1):18-26. doi:10.1056/NEJMoa0810988
20. Tuuli MG, Liu J, Stout MJ, et al. A Randomized Trial Comparing Skin Antiseptic Agents at Cesarean Delivery. *N Engl J Med*. 2016;374(7):647-655. doi:10.1056/NEJMoa1511048
21. Swenson BR, Hedrick TL, Metzger R, Bonatti H, Pruett TL, Sawyer RG. Effects of preoperative skin preparation on postoperative wound infection rates: a prospective study of 3 skin preparation protocols. *Infect Control Hosp Epidemiol*. 2009;30(10):964-971. doi:10.1086/605926
22. Swenson BR, Sawyer RG. Importance of Alcohol in Skin Preparation Protocols. *Infect Control Hosp Epidemiol*. 2010;31(09):977. doi:10.1086/655843
23. Mcdonnell G, Russell AD. Antiseptics and disinfectants: Activity, action, and resistance. *Clin Microbiol Rev*. 1999;12(1):147-179. doi:10.1007/s13398-014-0173-7.2
24. Dumville J, McFarlane E, Edwards P, Lipp A, Holmes A, Liu Z. Preoperative skin antiseptic for prevention of surgical wound infections after clean surgery. *Cochrane Database Syst Rev*. 2013;(3):CD003949. doi:10.1002/14651858.CD003949.pub4
25. Centers for Disease Control and Prevention (CDC). *Surgical Site Infection (SSI) Event*.; 2017.
26. Kunisada T, Yamada K, Oda S, Hara O. Investigation on the efficacy of povidone-iodine against antiseptic-resistant species. *Dermatology*. 1997;195 Suppl(suppl 2):14-18. http://www.ncbi.nlm.nih.gov/pubmed/9403250.
27. Russell AD, Day MJ. Antibacterial activity of chlorhexidine. *J Hosp Infect*. 1993;25:229-238.
28. Tsang STJ, Mills LA, Frantzias J, Baren JP, Keating JF, Simpson AHRW. Exchange nailing for nonunion of diaphyseal fractures of the tibia: our results and an analysis of the risk factors for failure. *Bone Joint J*. 2016;98-B(4):534-541. doi:10.1302/0301-620X.98B4.34870
29. Mills L, Tsang J, Hooper G, Keenan G, Simpson A. The multifactorial aetiology of fracture nonunion and the importance of searching for latent infection. *Bone Joint Res*. 2016;5(10):512-519. doi:10.1302/2046-3758.510.BJR-2016-0138
30. Schemitsch E, Bhandari M, Guyatt G, et al. Prognostic Factors for Predicting Outcomes After Intramedullary Nailing of the Tibia Study to Prospectively Evaluate Reamed Intramedullary Nails in Patients with Tibial Fractures (SPRINT) Investigators*. *JBJS*. 2012;94(19):1786-1793. doi:10.2106/JBJS.J.01418
31. MacKenzie EJ, Bosse MJ. Factors influencing outcome following limb-threatening lower limb trauma: lessons learned from the Lower Extremity Assessment Project (LEAP). *J Am Acad Orthop Surg*. 2006;14(10 Spec No.):S205-S210. doi:10.5435/00124635-200600001-00044
32. Solution. 3M^{TM} DuraPrep^{TM} Surgical. 3M^{TM} DuraPrep^{TM} Surgical Solution. http://multimedia.3m.com/mws/media/515983O/duraprep-surgical-solution.pdf. Published 2009.
33. Sun X, Briel M, Walter SD, Guyatt GH. Is a subgroup effect believable? Updating criteria to evaluate the credibility of subgroup analyses. *BMJ*. 2010;340(9209):c117. doi:10.1136/bmj.c117

34. Müller M, Nazarian S, Koch P, Schatzker J. The Comprehensive Classification of Fractures of Long Bones. *Springer-Verlag Berlin Heidelb*. 1990. http://www.springer.com/kr/book/9783540181651.
35. Järvinen TLN, Sihvonen R, Bhandari M, et al. Blinded interpretation of study results can feasibly and effectively diminish interpretation bias. *J Clin Epidemiol*. 2014;67(7):769-772. doi:10.1016/j.jclinepi.2013.11.011
36. Gustilo R, Anderson J. Prevention of infection in the treatment of one thousand and twenty-five open fractures of long bones: retrospective and prospective analyses. *J Bone Joint Surg Am*. 1976;58(4):453-458.
37. Centers for Disease Control and Prevention (CDC). *CDC/NHSN Surveillance Definitions for Specific Types of Infections*.; 2017.
38. Metsemakers WJ, Morgenstern M, McNally MA, et al. Fracture-related infection: A consensus on definition from an international expert group. *Injury*. 2018. doi:10.1016/j.injury.2017.08.040
39. Metsemakers WJ, Kuehl R, Moriarty TF, et al. Infection after fracture fixation: Current surgical and microbiological concepts. *Injury*. 2018. doi:10.1016/j.injury.2016.09.019
40. Journal of Orthopaedic Trauma. Journal of Orthopaedic Trauma: Online submission and review system. http://edmgr.ovid.com/jot/accounts/ifauth.htm. Published 2019. Accessed April 10, 2019.
41. Study to Prospectively Evaluate Reamed Intramedullary Nails in Patients with Tibial Fractures Investigators, Bhandari M, Guyatt G, et al. Randomized trial of reamed and unreamed intramedullary nailing of tibial shaft fractures. *J Bone Joint Surg Am*. 2008;90(12):2567-2578. doi:10.2106/JBJS.G.01694
42. TRUST Investigators writing group, Busse JW, Bhandari M, et al. Re-evaluation of low intensity pulsed ultrasound in treatment of tibial fractures (TRUST): randomized clinical trial. *BMJ*. 2016;355:i5351. doi:10.1136/bmj.i5351
43. FAITH Investigators. Fracture fixation in the operative management of hip fractures (FAITH): an international, multicentre, randomised controlled trial. *Lancet*. 2017;389(10078):1519-1527. doi:10.1016/S0140-6736(17)30066-1
44. Bhandari M, Devereaux PJ, Einhorn TA, et al. Hip fracture evaluation with alternatives of total hip arthroplasty versus hemiarthroplasty (HEALTH): protocol for a multicentre randomised trial. *BMJ Open*. 2015;5(2):e006263. doi:10.1136/bmjopen-2014-006263
45. Reich NG, Myers JA, Obeng D, Milstone AM, Perl TM. Empirical power and sample size calculations for cluster-randomized and cluster-randomized crossover studies. *PLoS One*. 2012;7(4):e35564. doi:10.1371/journal.pone.0035564
46. Grantham KL, Kasza J, Heritier S, Hemming K, Litton E, Forbes AB. How many times should a cluster randomized crossover trial cross over? *Stat Med*. 2019. doi:10.1002/sim.8349
47. Zwarenstein M, Treweek S, Gagnier JJ, et al. Improving the reporting of pragmatic trials: an extension of the CONSORT statement. *BMJ*. 2008;337:a2390. doi:10.1136/BMJ.A2390
48. Campbell MK, Piaggio G, Elbourne DR, Altman DG. Consort 2010 statement: extension to cluster randomised trials. *BMJ*. 2012;345(sep04 1):e5661-e5661. doi:10.1136/bmj.e5661
49. Thabane L, Akhtar-Danesh N. Guidelines for reporting descriptive statistics in health research. *Nurse Res*. 2008;15(2):72-81. doi:10.7748/nr2008.01.15.2.72.c6331
50. Sterne J, White I, Carlin J, et al. Multiple imputation for missing data in epidemiological and clinical research: potential and pitfalls. *Br Med J*. 2009;338(July):b2393.

doi:10.1136/bmj.b2393
51. Little RJA, Rubin DB. *Statistical Analysis with Missing Data*.; 2002. doi:10.2307/1533221
52. Wang R, Lagakos SW, Ware JH, Hunter DJ, Drazen JM. Statistics in Medicine — Reporting of Subgroup Analyses in Clinical Trials. *N Engl J Med*. 2007;357(21):2189-2194. doi:10.1056/NEJMsr077003
53. Sun X, Ioannidis JPA, Agoritsas T, Alba AC, Guyatt G. How to use a subgroup analysis: users' guide to the medical literature. *JAMA*. 2014;311(4):405-411. doi:10.1001/jama.2013.285063
54. Sun X, Briel M, Busse JW, et al. Subgroup Analysis of Trials Is Rarely Easy (SATIRE): a study protocol for a systematic review to characterize the analysis, reporting, and claim of subgroup effects in randomized trials. *Trials*. 2009;10:101. doi:10.1186/1745-6215-10-101
55. Morgan KE, Forbes AB, Keogh RH, Jairath V, Kahan BC. Choosing appropriate analysis methods for cluster randomised cross-over trials with a binary outcome. *Stat Med*. 2017;36(2):318-333. doi:10.1002/sim.7137
56. Zhang Y, Alyass A, Vanniyasingam T, et al. A systematic survey of the methods literature on the reporting quality and optimal methods of handling participants with missing outcome data for continuous outcomes in randomized controlled trials. *J Clin Epidemiol*. 2017;88:67-80. doi:10.1016/j.jclinepi.2017.05.016
57. Pocock SJ, Clayton TC, Stone GW. Challenging Issues in Clinical Trial Design Part 4 of a 4-Part Series on Statistics for Clinical Trials. *J Am Coll Cardiol*. 2015;66(25):2886-2898. doi:10.1016/j.jacc.2015.10.051
58. Chalmers I. What do I want from health research and researchers when I am a patient? *BMJ*. 1995;310(6990):1315-1318. doi:10.1136/bmj.310.6990.1315
59. Domecq JP, Prutsky G, Elraiyah T, et al. Patient engagement in research: A systematic review. *BMC Health Serv Res*. 2014;14:89. doi:10.1186/1472-6963-14-89
60. INVOLVE. *Briefing Notes for Researchers: Public Involvement in NHS, Public Health and Social Care Research*.; 2012. https://www.invo.org.uk/wp-content/uploads/2014/11/9938_INVOLVE_Briefing_Notes_WEB.pdf.
61. Esmail L, Moore E, Rein A. Evaluating patient and stakeholder engagement in research: Moving from theory to practice. *J Comp Eff Res*. 2015;4(2):133-145. doi:10.2217/cer.14.79
62. Tarpey M, INVOLVE Support Unit. *Why People Get Involved in Health and Social Care Research: A Working Paper*. Vol July.; 2006. http://www.invo.org.uk/wp-content/uploads/documents/whypeoplegetinvolvedinresearch2006.pdf.
63. Brett J, Staniszewska S, Mockford C, et al. A Systematic Review of the Impact of Patient and Public Involvement on Service Users, Researchers and Communities. *Patient*. 2014;7(4):387-395. doi:10.1007/s40271-014-0065-0
64. Robinson A. Patient and public involvement: In theory and in practice. *J Laryngol Otol*. 2014;128(4):318-325. doi:10.1017/S0022215114000735
65. Lawton R, O'Hara JK, Sheard L, et al. Can patient involvement improve patient safety? A cluster randomised control trial of the Patient Reporting and Action for a Safe Environment (PRASE) intervention. *BMJ Qual Saf*. 2017;26(8):622-631. doi:10.1136/bmjqs-2016-005570
66. Fergusson D, Monfaredi Z, Pussegoda K, et al. The prevalence of patient engagement in published trials: A systematic review. *Res Involv Engagem*. 2018;4:17. doi:10.1186/s40900-018-0099-x

67. Bartlett JE, Kotrlik JWKJW, Higgins C. Organizational research: Determining appropriate sample size in survey research appropriate sample size in survey research. *Inf Technol Learn Perform J*. 2001;19(1):43-50.
