# Supplementary material for: Effectiveness of Iodophor vs Chlorhexidine Solutions for Surgical Site Infections and Unplanned Reoperations for Patients Who Underwent Fracture Repair: The PREP-IT Master Protocol
Source: JAMA Netw Open. 2020 Apr 7;3(4):e202215. doi: 10.1001/jamanetworkopen.2020.2215 (PMC7139274; doi:10.1001/jamanetworkopen.2020.2215)
Supplement: Supplement 3. — Statistical Analysis Plan [file jamanetwopen-3-e202215-s003.pdf]

Statistical Analysis Plan

Section 5.0 of the Aqueous-PREP and PREPARE trial protocols outline detailed preliminary statistical analysis plans for each trial. These sections include descriptions of the primary comparison, the secondary comparison, subgroup analyses, and potential sensitivity analyses. The final statistical analysis plan will be completed prior to study close out.
